# Efficient syntheses of 25,26-dihydrodictyostatin and 25,26-dihydro-6-*epi*-dictyostatin, two potent new microtubule-stabilizing agents

**DOI:** 10.3762/bjoc.7.161

**Published:** 2011-10-05

**Authors:** María Jiménez, Wei Zhu, Andreas Vogt, Billy W Day, Dennis P Curran

**Affiliations:** 1 Department of Chemistry, University of Pittsburgh, 219 Parkman Avenue, Pittsburgh, PA 15260 USA; 2 Department of Computational and Systems Biology, and Drug Discovery Institute, University of Pittsburgh, 3501 Fifth Avenue, Pittsburgh, PA 15260, USA; 3 Department of Pharmaceutical Sciences, School of Pharmacy, 3501 Terrace Street, University of Pittsburgh, Pittsburgh, PA 15261 USA

**Keywords:** anticancer agents, dictyostatin, microtubules, NHK

## Abstract

The dictyostatins are powerful microtubule-stabilizing agents that have shown antiproliferative activity against a variety of human cancer cell lines. Two highly active analogs of dictyostatin, 25,26-dihydrodictyostatin and 25,26-dihydro-6-*epi*-dictyostatin, were prepared by a new streamlined total synthesis route. Three complete carbon fragments were prepared to achieve maximum convergency. These were coupled by a Horner–Wadsworth–Emmons reaction sequence and an esterification. A late stage Nozaki–Hiyama–Kishi reaction was then used to form the 22-membered macrolide. The stereoselectivity of this reaction depended on the configurations of the nearby stereocenter at C6.

## Introduction

The discovery of compounds that function as anticancer agents by altering the dynamics of microtubules continues to be an important goal in medicinal chemistry. Such agents can force the cell to exit mitosis aberrantly, leading to apoptosis [1–2]. Important classes of microtubule-stabilizing agents include taxanes, epothilones, and discodermolides, among others [3–4]. Dictyostatin (**1a**) is an exceptionally potent microtubule-stabilizing agent that has shown antiproliferative activity in a variety of human cancer cell lines in the low nanomolar range. Isolated first in 1994 by Pettit and coworkers [5–6], its complete stereostructure was proposed by Paterson and Wright in 2004 [7]. The structure assignment phase was finalized in 2004 when total syntheses by Paterson and our group confirmed the assignment [8–9].

The two synthetic samples of dictyostatin provided exciting biological results [10], which in turn spurred structure–activity studies in both Paterson’s group [11–17] and ours [18–23]. These studies, founded on total synthesis, were largely complementary and together provide a solid if still evolving [24] picture of dictyostatin SAR. Phillips [25], Ramachandran [26] and Gennari [27] have also developed efficient synthetic routes to the natural product or fully functionalized analogs.

**Figure 1:**
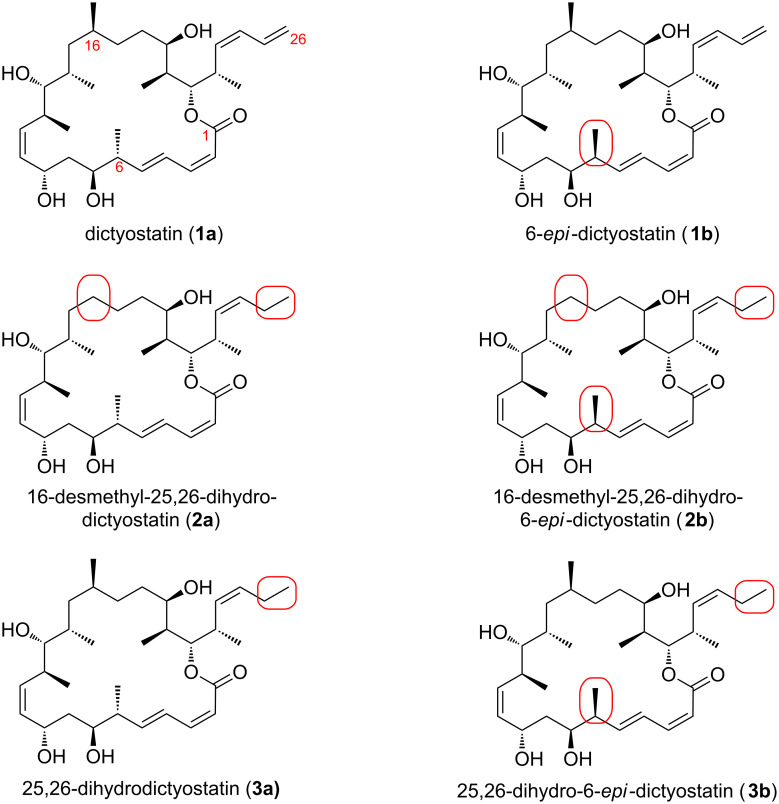
Structures of dictyostatin and selected analogs varying at C6, C16, and C25–C26.

Based on the biological profile of over 30 analogs of dictyostatin synthesized in Pittsburgh, we selected 6-*epi*-dictyostatin (**1b**) for scale-up and in vivo testing [28]. Indeed, **1b** proved to be more effective than paclitaxel in treating mice bearing human breast cancer xenografts. Encouraged by these results, we have pursued both new analogs and improved synthetic routes. We recently reported a streamlined synthesis of dictyostatin (**1a**) and used it to prepare two new analogs: 16-desmethyl-25,26-dihydrodictyostatin (**2a**) and its C6 epimer **2b (**Figure 1) [29]. The terminal C25–C26 alkene of the C23–C26 diene is a synthetic liability, so it was welcome news when the biological data revealed that the analogs lacking this alkene retained significant activity. Prior results suggested that most if not all of the lost activity could be regained by reinstating the missing C16-methyl group [11,19].

We thus set out to synthesize and test two new analogs of dictyostatin: 25,26-dihydrodictyostatin (**3a**) and 25,26-dihydro-6-*epi*-dictyostatin (**3b**) (Figure 1). The later parts of this synthesis have been briefly communicated in a recent paper whose primary focus was biological evaluation [30]. Indeed, **3a** and **3b** prove to be promising anticancer agents with in vitro and cellular testing data superior to those of the 16-desmethyl analogs **2a** and **2b**, and roughly comparable to those of dictyostatin (**1a**) and 6-*epi*-dictyostatin (**1b**). Here we report the full details of the synthesis of **3a** and **3b**.

## Results and Discussion

**Figure 2:**
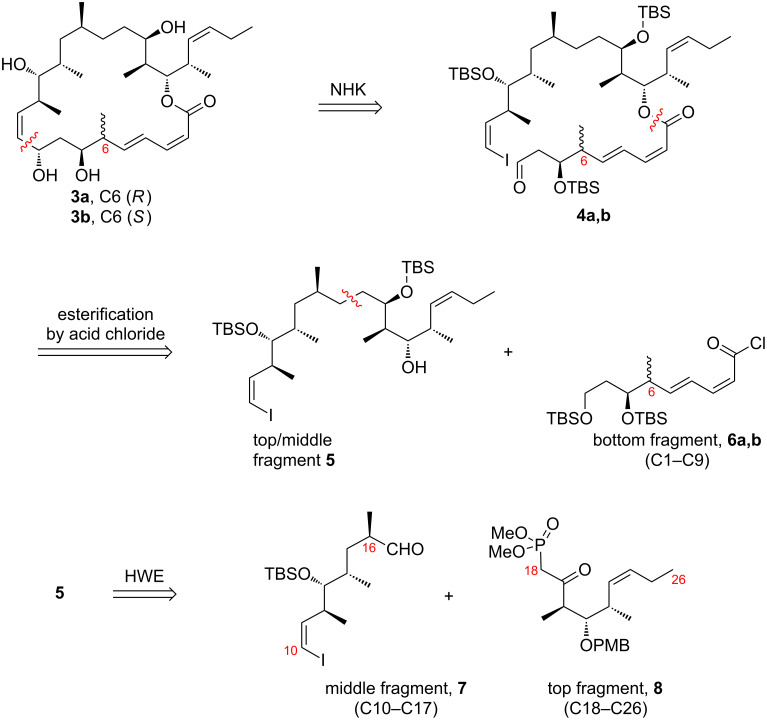
Retrosynthetic analysis for **3a** and **3b**.

Figure 2 shows key aspects of the retrosynthetic analysis to make analogs **3a** and **3b**, which follows after the successful streamlined route to make **1a** [29]. For high convergence, the analogs were dissected strategically into three complete fragments called top (**8**, C18–C26), middle (**7**, C10–C17), and bottom (**6a,b**, C1–C9), respectively. The top **8** and middle **7** fragments were first combined through an established Horner–Wadsworth–Emmons (HWE) reaction sequence to give **5** [8–9]. The bottom fragment **6a** or **6b** was then attached to the top/middle fragment **5** through an esterification reaction. Finally, an intramolecular Nozaki–Hiyama–Kishi (NHK) reaction [31–32] of compounds **4a** and **4b** was used to form the macrolactone at C9–C10.

**Scheme 1:**
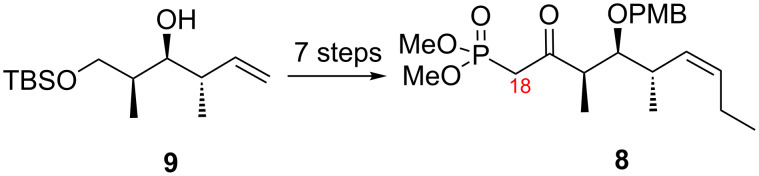
Synthesis of top fragment **8** (C18–C26).

We have previously described in detail the synthesis of the top fragment **8**, which is shared by both analogs **3a**,**b** [29,33]. As summarized in Scheme 1, this fragment was made on multigram scale in seven steps starting from the well-known intermediate **9**.

**Scheme 2:**
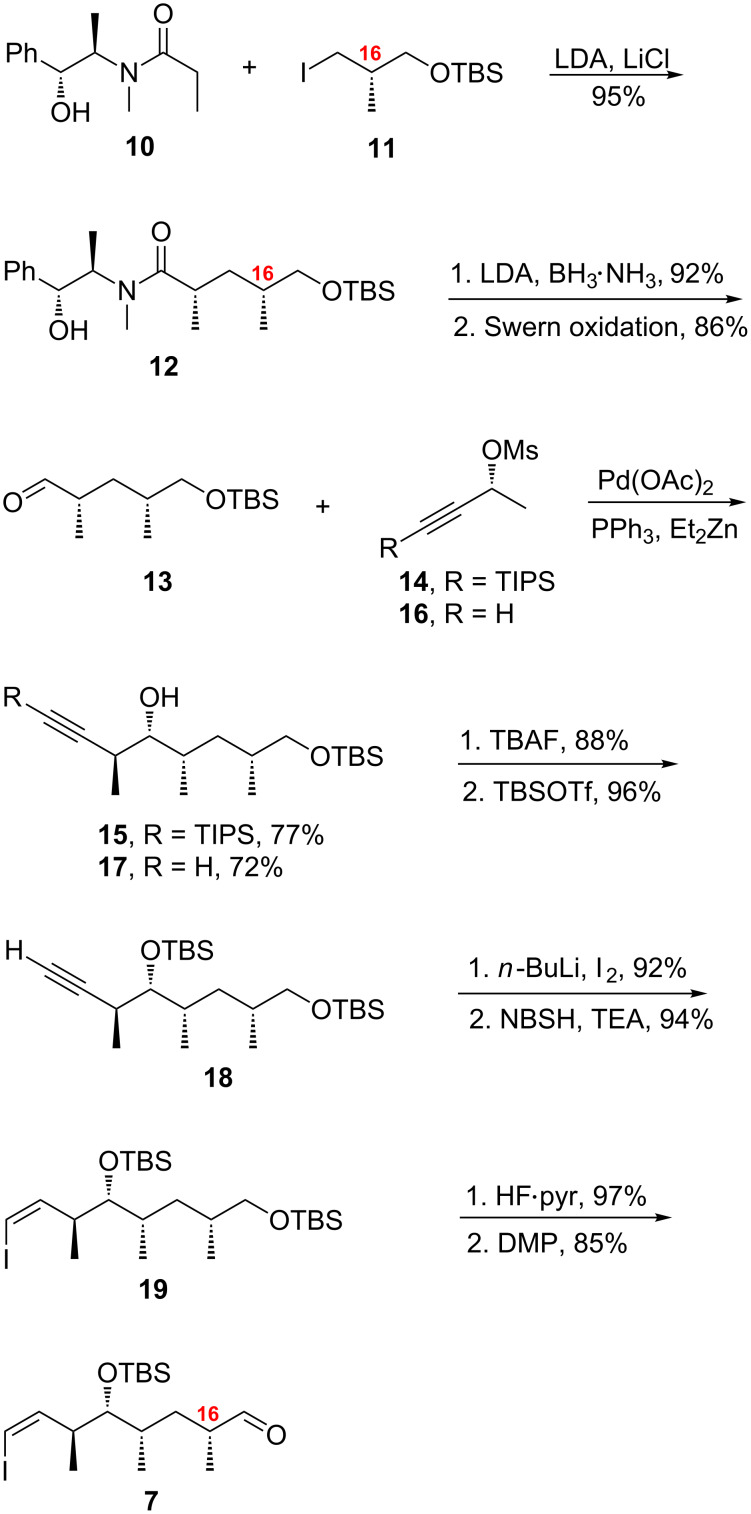
Synthesis of middle fragment **7** (C10–C17).

The shared middle fragment **7** was conveniently made by the pathway shown in Scheme 2, which is a streamlined version of prior routes in the 16-desmethyl series [14]. (However, some material **7** was also made by an extension of a prior middle fragment route, as described in Supporting Information File 1, Scheme S1). Myers alkylation [17] of commercially available pseudoephedrine amide **10** and alkyl iodide **11** afforded amide **12** in 95% yield. The chiral auxiliary was then removed with BH_3_·NH_3_ (92%) [34], then the resulting primary alcohol was oxidized under Swern conditions to provide aldehyde **13** (86%). A Marshall palladium-catalyzed addition reaction [35] between aldehyde **13** and mesylate **14** [36] gave alcohol **15** in 77% yield. Interestingly, the Marshall reaction works well even with mesylates containing a terminal alkyne; reaction of **13** with **16** provided **17** in 72% yield.

The TIPS and primary TBS groups of **15** were simultaneously cleaved under basic conditions with TBAF [37] (88%), followed by protection of both primary and secondary hydroxy groups with TBSOTf to afford **18** in 96% yield. The terminal alkyne of **18** was then converted to the iodoalkyne with BuLi and I_2_ (92%), followed by diimide reduction with *o*-nitrobenzene sulfonylhydrazide (NBSH) [38] to the *cis*-vinyl iodide **19** in 94% yield. The primary TBS group of **19** was selectively removed with HF·pyridine in pyridine/THF to afford a primary alcohol (97%), which was then oxidized to aldehyde **7** with the aid of the Dess–Martin reagent (85%). Overall, the synthesis of the middle fragment was accomplished in 10 steps in over 40% yield starting from commercially available pseudoephedrine amide **10**.

**Scheme 3:**
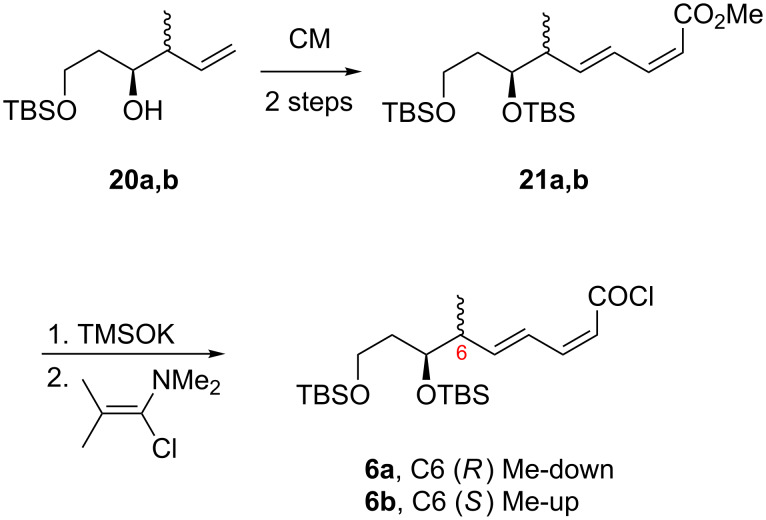
Synthesis of bottom fragments **6a,b** (C1–C9).

The syntheses of the bottom fragments **6a** and **6b** were achieved by applying the previously reported cross-metathesis reactions of readily available **20a**,**b** with (2*Z*,4*E*)-methyl hexa-2,4-dienoate followed by silylation to provide **21a**,**b** [39]. These dienoates were converted to the carboxylic acids by using TMSOK [40], followed by transformation into the acid chloride **6a** and **6b** with the Ghosez reagent [41] (Scheme 3). This acid chloride was then used directly in the subsequent esterification reaction.

**Scheme 4:**
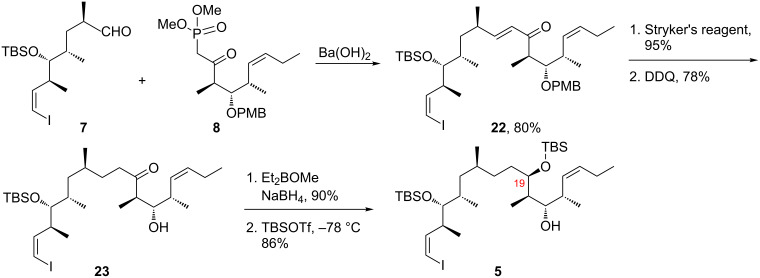
Coupling of the top and middle fragments.

With the top, middle and bottom fragments in hand, we turned to the initial fragment couplings as summarized in Scheme 4. Fragments **7** and **8** were first combined by a proven sequence starting with an HWE reaction mediated by Ba(OH)_2_ [42] to provide enone **22** in good yield (80%). This enone was then treated with Stryker’s reagent [43] to selectively reduce the conjugated alkene, followed directly by removal of the PMB group with DDQ. This sequence afforded alcohol **23** in 76% yield over two steps. A stereoselective 1,3-*syn* reduction was then performed under Prasad conditions [44] to give the target diol (90%) as a single isomer. The stereochemistry at C19 was confirmed by NMR analysis of the corresponding acetonide [45] (Supporting Information File 1). This diol was then selectively protected at the less hindered hydroxy group (C19), with TBSOTf at −78 °C [46], to provide **5** in 86% yield.

**Scheme 5:**
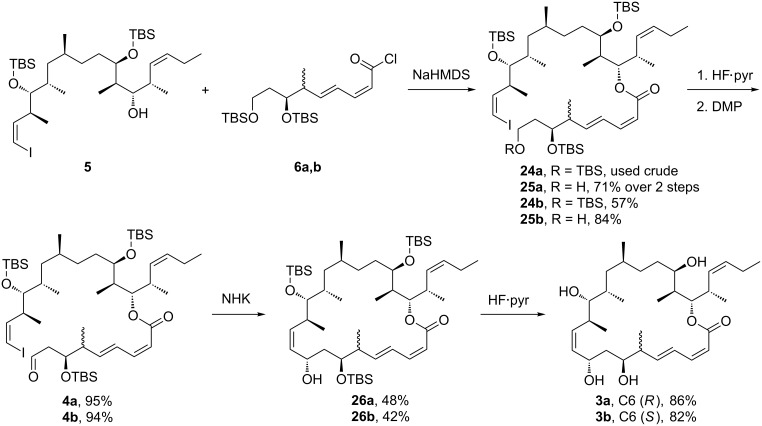
Coupling with the bottom fragment and end game.

The synthetic route to **3a** and **3b** continues in Scheme 5 with the coupling of alcohol **5** with the bottom fragments **6a** and **6b**. The hydroxy group in **5** was deprotonated with NaHMDS followed by addition of the crude reaction mixture containing acid chloride **6a** or **6b**. The coupled products **24a** and **24b** proved difficult to purify by flash chromatography. In the C6 (*S*)-series, **24b** was successfully isolated in 57% yield (82% BRSM). In the C6 (*R*)-series, the crude coupled product **24a** could not be separated from **5**, and was immediately subjected to HF·pyridine deprotection. The resulting primary alcohol **25a** was isolated in 71% yield (90% BRSM) after careful flash chromatography. Selective deprotection of purified **24b** with HF·pyridine afforded the respective primary alcohol **25b** in 84% yield.

Next, treatment of each primary alcohol **25a**,**b** with Dess–Martin reagent provided aldehydes **4a** and **4b** in comparable yields (95% and 94%). Initial NHK reactions were performed in THF with 15 equiv of CrCl_2_, 0.2 equiv of NiCl_2_(dppf) and 15 equiv of 4,4'-di-*tert*-butyl-2,2'-dipyridine [47]. The target product **26b** for the C6 (*S*)-series was obtained in respectable yield (42%). Only the target C9-β epimer was detected in the ^1^H NMR spectrum of the crude product, though we cannot rule out the presence of small amounts of the α-epimer. However, in the C6 (*R*)-series, the C9 β/α ratio was only about 75/25 and the isolated yield of pure β-epimer **26a** was only 22%. After surveying several other conditions and solvents, we found that treatment of 4**a** with 15 equiv of CrCl_2_ and 0.2 equiv of NiCl_2_ in DMF/THF [48] improved the β/α ratio to about 85/15 and improved the isolated yield of pure **26a** to 48%. Again, formation of acetonides confirmed the stereochemical assignment (Supporting Information File 1) [45]. Finally, treatment of **26a** and **26b** with HF·pyridine afforded the desired analogs **3a** and **3b** in good yields (86% and 82%, respectively). These compounds were fully characterized by the usual spectroscopic means.

The target compounds 25,26-dihydrodictyostatin (**3a**) and its C6-epimer **3b** were tested in comparison to dictyostatin (**1a**) and 6-*epi*-dictyostatin (**1b**), epothilone B, and paclitaxel, and these data have recently been reported in detail [30]. Briefly, both compounds were potent microtubule-perturbing agents that induced mitotic arrest and microtubule assembly in vitro and in intact cells. Each displaced [^3^H]paclitaxel and [^14^C]epothilone B from microtubules with potencies comparable to (−)-dictyostatin and discodermolide. Each compound also inhibited the growth of cell lines resistant to paclitaxel and epothilone B at low nanomolar concentrations, synergized with paclitaxel in MDA-MB-231 human breast cancer cells, and had anti-angiogenic activity in transgenic zebra fish larvae.

## Conclusion

We have successfully used a streamlined synthesis to access two new analogs, **3a** and **3b**, in the dictyostatin class of natural products. This synthesis based on three large fragments is highly convergent, requiring minimal functional group transformations once the coupling events take place. The synthesis of each fragment is amenable to scale-up and takes ten steps or less. Ten more steps are needed from the start of fragment coupling to the end of the synthesis, providing the target compounds in about 7–8% overall yield.

The intramolecular NHK reaction was successful for the formation of the macrolactone. As with the prior 16-desmethyl series compounds, the stereoselectivity of this reaction depended on the configuration of the nearby stereocenter at C6 with the (6*S*,7*S*)-epimer giving almost exclusively the target isomer at C9, whereas the (6*R*,7*S*)-epimer gave about 25% of a minor isomer along with about 75% of the target isomer. The subsequent testing data identify 25,26-dihydrodictyostatin and 25,26-dihydro-6-*epi*-dictyostatin as candidates for scale-up synthesis and further preclinical development.

## Supporting Information

File 1 Experimental details, characterization data and copies of NMR spectra of all new compounds.
