# Supplementary material for: Efficient syntheses of 25,26-dihydrodictyostatin and 25,26-dihydro-6-epi-dictyostatin, two potent new microtubule-stabilizing agents
Source: Beilstein J Org Chem. 2011 Oct 5;7:1372–8. doi: 10.3762/bjoc.7.161 (PMC3201051; doi:10.3762/bjoc.7.161)
Supplement: File 1 — Experimental details, characterization data and copies of NMR spectra of all new compounds. [file Beilstein_J_Org_Chem-07-1372-s001.pdf]

Supporting Information

for

Efficient syntheses of 25,26-dihydrodictyostatin and 25,26-dihydro-6-*epi*-dictyostatin, two potent new microtubule-stabilizing agents

María Jiménez[1], Wei Zhu[1], Andreas Vogt[2], Billy W. Day[1,2,3] and Dennis P. Curran[1]*

Address: [1]Department of Chemistry, University of Pittsburgh, 219 Parkman Avenue, Pittsburgh, PA 15260 USA, [2]Department of Computational and Systems Biology, and Drug Discovery Institute, University of Pittsburgh, 3501 Fifth Avenue, Pittsburgh, PA 15260, USA and [3]Department of Pharmaceutical Sciences, School of Pharmacy, 3501 Terrace Street, University of Pittsburgh, Pittsburgh, PA 15261 USA

Email: Dennis P. Curran - curran@pitt.edu

* Corresponding author

Experimental details, characterization data and copies of NMR spectra of all new compounds

Table of Contents

General Information S2

Synthesis of the middle fragment **7** S3

Scheme S1: Alternate route to the synthesis of middle fragment **7** S9

Coupling of the top and middle fragments S11

Coupling with the bottom fragment: S16

Intramolecular NHK reactions S22

25,26-Dihydrodictyostatin S24

6-*Epi*-25,26-dihydrodictyostatin S26

Copies of NMR spectra S27

General information: All reactions were run under argon unless otherwise noted. Toluene, THF, dichloromethane, and diethyl ether were purified by filtration through activated alumina under a nitrogen atmosphere. THF used for the NHK reaction was distilled from sodium/benzophenone. DMF used for the NHK reaction was distilled over CaH_2 and dried over 4Å molecular sieves prior to use. All commercial reagents were used as received. 4Å Molecular sieves were oven-dried for at least 24 h before use. All new compounds were fully characterized by ^1H and ^{13}C NMR, IR, optical rotation, and mass spectrometry. ^1H and ^{13}C NMR spectra were recorded on Bruker Avance DRX (300 MHz), Bruker Avance III (300 MHz), Bruker Avance III (400 MHz), Bruker Avance III (500 MHz), Bruker Avance III (600 MHz) and Bruker Avance III (700 MHz) spectrometers. Chemical shifts were reported in ppm. $CDCl_3$ was used as the NMR solvent unless otherwise noted. In reporting spectral data, the following abbreviations were used: s = singlet, d = doublet, t = triplet, q = quartet, m = multiplet, bs = broad singlet. Coupling constants were measured in Hertz (Hz). Infrared spectra were taken on a Mattson Genesis Series FTIR by means of thin-film deposition on NaCl plates unless otherwise noted. Peaks are reported in wavenumbers (cm^{-1}). Low and high resolution mass spectra were obtained on Waters LC/Q-Tof and are reported in *m/z* units. High resolution mass spectra were obtained on a VG Autospec double focusing instrument and are reported in units of *m*/*z*. Optical rotations were measured on a Perkin-Elmer 241 polarimeter at the Na D-line (λ = 589 nm) with a 1 dm cell at 20 °C. HPLC analyses were conducted by using Waters 600 controller and Waters 2487 dual λ absorbance detector or polymer laboratory PL-ELS 1000 detector controlled with the Millennium™ program. Thin layer chromatography (TLC) was performed on silica gel 60 F_{254} glass-backed plates with a layer thickness of 0.25 mm manufactured by E. Merck. TLC visualization was performed by illumination with a 254 nm UV lamp or by staining with $KMnO_4$ and subsequent heating. Silica gel chromatography was performed on silica gel (230–400 mesh ASTM) purchased from Sorbtech or Bodman or with the automated Teledyne Isco equipment.

I. Synthesis of the middle fragment 7

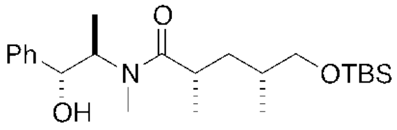

(2*S*,4*R*)-5-((*tert*-Butyldimethylsilyl)oxy)-*N*-((1*R*,2*R*)-1-hydroxy-1-phenylpropan-2-yl)-*N*,2,4-trimethylpentanamide (12). LiCl (5.66 g, 133.6 mmol) was flame dried under vacuum for 10 min using a Bunsen burner and the flask was allowed to cool down to rt under Ar. Diisopropylamine (98.0 mmol, 13.8 mL) was added followed by dry THF (200 mL). The solution was cooled to –78 °C followed by the dropwise addition of *n*-BuLi (89.1 mmol, 56 mL) through an addition funnel. After warming to 0 °C and stirring for 15 min, the solution was recooled to –78 °C and treated with a solution of propionamide **10** (9.86 g, 44.5 mmol) in THF (111 mL) dropwise through an addition funnel. After stirring at –78 °C for 1 h, the reaction mixture was warmed slowly to rt, stirred for 5 min and cooled to 0 °C. A solution of alkyl iodide **11** (7.0 g, 22.3 mmol) in THF (111 mL) was added dropwise through an addition funnel and the resulting reaction mixture stirred at 0 °C for 12 h. The reaction mixture was poured over an ice-cold solution of saturated NH_4Cl (200 mL) and stirring was continued for 30 min. The organic layer was collected and the water layer extracted with DCM (4 × 150 mL). The organic layers were combined, dried over $MgSO_4$ and filtered, and the solvent was removed. Purification by means of flash chromatography (SiO_2, 2:1 hexanes/EtOAc) afforded amide **12** (8.56 g, 95%) as a yellow oil, which turned into an ivory solid when placed under vacuum overnight. TLC R_f = 0.52 (1:1 hexanes/EtOAc); mp = 66–68 °C; $[\alpha]_D^{25}$ –58.1 (*c* 1.18, CH_2Cl_2); ^{1}H NMR (400 MHz, $CDCl_3$, ~6:1 rotamer ratio, only major rotamer shown) δ 7.37–7.29 (m, 5H), 4.62 (t, *J* = 7.2 Hz, 1H), 4.35 (bs, 1H), 3.44 (dd, *J* = 9.8, 4.7 Hz, 1H), 3.37 (dd, *J* = 9.8, 5.8 Hz, 1H), 2.84 (s, 3H), 2.78–2.70 (m, 1H), 1.69–1.63 (m, 1H), 1.58–1.52 (m, 1H), 1.14 (d, *J* = 7.0 Hz, 3H), 1.12–1.10 (m, 1H), 1.08 (d, *J* = 6.6 Hz, 3H), 0.87 (s, 9H), 0.82 (d, *J* = 6.6 Hz, 3H), 0.02 (s, 6H) ppm; ^{13}C NMR(150 MHz, C_6D_6) δ 177.9, 143.7, 128.2, 127.2, 126.8, 76.1, 68.3, 58.2, 37.9, 34.2, 33.6, 26.3, 26.2, 18.5, 18.0, 17.4, 14.3, –5.2, –5.3 ppm; IR (neat) 3371, 2956, 2930, 2856, 1620, 1467, 1409, 1254, 1086, 1054 cm^{-1}.

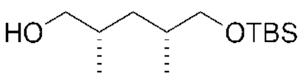

(2*S*,4*R*)-5-((*tert*-Butyldimethylsilyl)oxy)-2,4-dimethylpentan-1-ol. A solution of diisopropylamine (8.9 mL, 63.0 mmol) in THF (63 mL) at –78 °C was treated with *n*-BuLi (38.4 mL, 61.5 mmol, 1.6 M in hexanes) dropwise through an addition funnel. This solution was allowed to warm up to 0 °C and stirred for 15 min, then treated with BH_3-NH_3 (2.0 g, 60.0 mmol) in one portion and stirred for an additional 15 min. The reaction mixture was warmed to rt, stirred for 15 min and recooled to 0 °C. A solution of amide **12** (6.11 g, 15.0 mmol) in THF (150 mL) was then added dropwise through an addition funnel and the reaction mixture stirred at rt until the reaction was judged to be complete by TLC analysis (~4 h). The reaction mixture was poured into a flask containing a cold (0 °C) solution of saturated NH_4Cl (100 mL), MeOH (50 mL) and ether (50 mL) and stirring continued for 30 min. The organic layer was collected and the water layer extracted with ether (4 × 150 mL). The organic layers were combined, dried over $MgSO_4$ and filtered, and the solvent removed. Purification by flash chromatography (SiO_2, 10:1, then 5:1 hexanes/EtOAc) afforded the desired alcohol (3.4 g, 92%) as a yellow oil. TLC R_f = 0.33 (5:1 hexanes/EtOAc); $[\alpha]_D^{25}$ –3.13 (*c* 1.12, $CHCl_3$); ^1H NMR (500 MHz, $CDCl_3$) δ 3.50 (dd, *J* = 10.5, 5.5 Hz, 1H), 3.42 (dd, *J* = 9.5, 6.0 Hz, 1H), 3.40 (dd, *J* = 10.5, 6.5 Hz, 1H), 3.36 (dd, *J* = 9.5, 6.5 Hz, 1H), 1.75–1.66 (m, 2H), 1.57 (bs, 1H), 1.43 (ddd, *J* = 13.5, 6.5, 6.5 Hz, 1H), 0.94 (d, *J* = 6.5 Hz, 3H), 0.89 (d, *J* = 6.5 Hz, 3H), 0.89–0.88 (m, 1H), 0.88 (s, 9H), 0.03 (s, 6H) ppm; ^{13}C NMR (75 MHz, $CDCl_3$) δ 68.2, 67.9, 37.2, 33.2, 33.2, 25.9, 18.2, 17.7, 17.6, –5.4, –5.4 ppm; IR (neat) 3342, 2955, 2929, 3858, 1468, 1388, 1362, 1253 cm^{-1}; HRMS (EI) calcd for $C_{13}H_{29}OSi$ [M^+ – OH] 229.1988, found 229.1988.

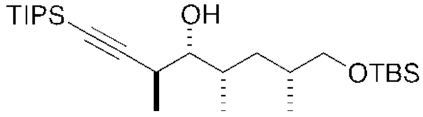

(3*S*,4*R*,5*S*,7*R*)-8-((*tert*-Butyldimethylsilyl)oxy)-3,5,7-trimethyl-1-(triisopropylsilyl)oct-1-yn-4-ol (15).

Swern Oxidation: DMSO (1.53 mL, 21.54 mmol) was added dropwise with a syringe to a solution of oxalyl chloride (1.23 mL, 14.36 mmol) in DCM (57.4 mL) at –78 °C. After stirring for 20 min a solution of the alcohol prepared above (1.77 g, 7.18 mmol) in DCM (14.4 mL) was added dropwise through an addition funnel and stirred for another 20 min. DIPEA (6.25 mL, 35.9 mmol) was then added dropwise through an addition funnel and after stirring for 30 min at

–78 °C, the reaction mixture was allowed to warm up to rt and stirred for 2 h. The solvent was removed and the yellow residue was diluted with pentane (100 mL) and water (100 mL). The organic layer was collected and the water layer was extracted with ether (4 × 100 mL). The organic layers were combined, dried over $MgSO_4$ and filtered, and the solvent was removed. The crude product was filtered through a plug of silica and celite with ether as eluent, and the resulting aldehyde **13** (1.53 g, 86%) was used immediately for the next step without further purification. ^1H NMR (300 MHz, $CDCl_3$) δ 9.57 (d, J = 2.4 Hz, 1H), 3.42 (d, J = 5.8 Hz, 2H), 2.46 (dddd, J = 13.7, 13.7, 7.0, 2.5 Hz, 1H), 1.86 (ddd, J = 13.8, 7.8, 6.1 Hz, 1H), 1.73–1.62 (m, 1H), 1.14-1.07 (m, 1H), 1.09 (d, J = 6.7 Hz, 3H), 0.89 (d, J = 6.9 Hz, 3H), 0.88 (s, 9H), 0.03 (s, 6H) ppm.

Marshall alkylation: A solution of $Pd(OAc)_2$ (70 mg, 0.31 mmol) in THF (55 mL) at –78 °C was treated with pulverized PPh_3 (81 mg, 0.31 mmol). Once completely dissolved, a solution of the mesylate **14** (2.27g, 7.46 mmol) in THF (3.6 mL) and a solution of the crude aldehyde **13** prepared above (1.53 g, 6.25 mmol) in THF (3.6 mL) were added dropwise by syringe. Et_2Zn (18.7 mL, 18.7 mmol, 1 M solution in hexanes) was then added dropwise by syringe over 10 min. The reaction mixture was warmed to –20 °C and stirred for 14 h. The cold reaction mixture was poured over saturated NH_4Cl (200 mL) and stirred for 20 min. The solution was transferred to a separatory funnel and the organic layer was collected. The water layer was extracted with DCM (4 × 125 mL), the combined organic layers were dried over $MgSO_4$ and filtered, and the solvent was removed. Purification by flash chromatography (SiO_2, 25:1 then 15:1 hexanes/EtOAc) afforded alkyne **15** (2.5 g, 77% over 2 steps) as a yellow oil. TLC R_f = 0.58 (12:1 hexanes/EtOAc); $[\alpha]_D^{25}$ –10.97 (*c* 1.13, $CHCl_3$); ^1H NMR (300 MHz, $CDCl_3$) δ 3.49 (dd, J = 9.6, 5.1 Hz, 1H), 3.31 (dd, J = 9.6, 6.9 Hz, 1H), 3.18 (dd, J = 10.8, 6.0 Hz, 1H), 2.73 (ddd, J = 13.5, 6.9, 6.9 Hz, 1H), 1.95 (d, J = 6.0 Hz, 1H), 1.77–1.67 (m, 2H), 1.48 (ddd, J = 13.5, 6.6, 6.6 Hz, 1H), 1.19 (d, J = 6.9 Hz, 3H), 1.06 (s, 3H), 1.05 (s, 18H), 1.06–1.05 (m, 1H), 0.92 (d, J = 6.9 Hz, 3H), 0.90 (d, J = 6.9 Hz, 3H), 0.89 (s, 9H), 0.03 (s, 6H) ppm; ^{13}C NMR (75 MHz, $CDCl_3$) δ 110.0, 83.3, 77.4, 67.9, 37.8, 33.3, 33.2, 32.1, 25.9, 18.6, 18.2, 18.0, 17.8, 14.4, 11.1, –5.4, –5.4 ppm; IR (neat) 2954, 2864, 2161, 1463, 1094 cm^{-1}; HRMS (EI) calcd for $C_{26}H_{54}O_2Si_2$ 454.3659, found 454.3662.

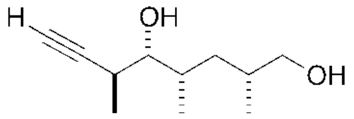

(2*R*,4*S*,5*R*,6*S*)-2,4,6-Trimethyloct-7-yne-1,5-diol. TBAF (29.8 mL, 29.8 mmol, 1 M solution in THF) was added dropwise through an addition funnel at rt to a solution of alkyne **15** (6.46g, 14.2 mmol) in THF (473 mL) under stirring. The resulting orange solution was stirred for 2 h and then quenched by the addition of saturated NH_4Cl. The organic layer was collected and the water layer extracted with DCM (3× 150 mL). The organic layers were combined, dried over $MgSO_4$ and filtered, and the solvent was removed. Purification by flash chromatography (SiO_2, 2:1 hexanes/EtOAc, then 100% EtOAc) afforded the desired diol (2.3 g, 88%) as a clear oil. TLC R_f = 0.23 (1:1 hexanes/EtOAc); $[\alpha]_D^{25}$ –4.88 (*c* 1.74, $CHCl_3$); ^{1}H NMR (300 MHz, $CDCl_3$) δ 3.51 (dd, *J* = 10.8, 5.1 Hz, 1H), 3.44 (dd, *J* = 10.5, 6.0 Hz, 1H), 3.26 (t, *J* = 5.1 Hz, 1H), 2.68 (dddd, *J* = 13.5, 6.9, 6.9, 2.4 Hz, 1H), 2.14 (d, *J* = 2.4 Hz, 1H), 2.00 (bs, 1H), 1.80–1.67 (m, 3H), 1.55 (ddd, *J* = 13.5, 6.9, 6.6 Hz, 1H), 1.20 (d, *J* = 6.9 Hz, 3H), 0.94 (d, *J* = 6.6 Hz, 3H), 0.93 (d, *J* = 6.6 Hz, 3H) ppm; ^{13}C NMR (75 MHz, $CDCl_3$) δ 86.2, 76.0, 70.5, 67.5, 37.1, 32.6, 32.5, 30.3, 17.4, 17.2, 14.1 ppm; IR (neat) 3350, 3308, 2963, 2932, 2875, 1460, 1378, 1241, 1031 cm^{-1}.

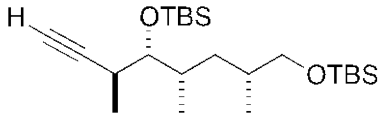

(5*R*,6*S*,8*R*)-5-((*S*)-But-3-yn-2-yl)-2,2,3,3,6,8,11,11,12,12-decamethyl-4,10-dioxa-3,11-disilatridecane (18). A solution of the diol prepared above (1.30 g, 7.05 mmol) in CH_2Cl_2 (35 mL) at –78 °C was treated with 2,6-lutidine (4.11 mL, 35.3 mmol) and TBSOTf (4.86 mL, 21.1 mmol) dropwise by syringe. After stirring for 10 min. the reaction mixture was allowed to warm up to rt and stirred for 2 h. The reaction was quenched with saturated NH_4Cl (1 mL) and stirring was continued overnight. The solvent was removed and the product purified by flash chromatography (SiO_2, 25:1 hexanes/EtOAc) to afford alkyne **18** (2.81 g, 96%) as a yellow oil. TLC R_f = 0.71 (10:1 hexanes/EtOAc); $[\alpha]_D^{25}$ –8.10 (*c* 2.13, CH_2Cl_2); ^{1}H NMR (300 MHz, $CDCl_3$) δ 3.49 (d, *J* = 5.0 Hz, 1H), 3.48 (dd, *J* = 9.6, 5.0 Hz, 1H), 3.31 (dd, *J* = 9.7, 6.8 Hz, 1H), 2.60 (ddd, *J* = 7.3, 5.0, 2.4 Hz, 1H), 2.03 (d, *J* = 2.4 Hz, 1H), 1.88–1.80 (m, 1H), 1.72–1.62 (m, 1H), 1.46 (ddd, *J* = 13.5, 6.6, 6.6 Hz, 1H), 1.17 (d, *J* = 7.1 Hz, 3H), 0.98–0.92 (m, 1H), 0.91 (s, 9H), 0.89 (s, 9H), 0.89 (d, *J* = 7.5 Hz, 3H), 0.88 (d, *J* = 6.5 Hz, 3H), 0.08 (s, 3H), 0.06 (s, 3H), 0.03 (s, 6H) ppm; ^{13}C NMR (75 MHz, $CDCl_3$) δ 87.3, 77.5, 70.1, 68.1, 38.9, 33.5, 33.3, 32.0, 26.1, 26.0, 18.4, 18.3, 17.8, 17.3, 15.6, –3.8, –3.9, –5.4, –5.4 ppm; IR (neat) 3313, 2955, 2931, 2886, 2857, 1468, 1387, 1253 cm^{-1}; HRMS (ES+) calcd for $C_{23}H_{48}O_2Si_2Na$ $[M + Na]^+$ 435.3091, found 435.3108.

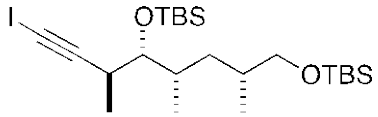

(5*R*,6*S*,8*R*)-5-((*S*)-4-Iodobut-3-yn-2-yl)-2,2,3,3,6,8,11,11,12,12-decamethyl-4,10-dioxa-3,11-disilatridecane. A solution of alkyne **18** (2.24 g, 5.42 mmol) in THF (18 mL) at –78 °C was treated with *t*-BuLi (3.84 mL, 6.51 mmol, 1.7 M in pentane) dropwise by syringe and the resulting bright orange solution was stirred for 1 h. A solution of I_2 (1.65 g, 6.51 mmol) in THF (9.3 mL) was added dropwise by syringe and the solution was allowed to warm up to rt and stirred for 1 h. The reaction was quenched with saturated $Na_2S_2O_3$ and stirring was continued until the solution turned colorless. The organic layer was collected and the water layer extracted with CH_2Cl_2 (4 × 100 mL). The organic layers were combined, dried over $MgSO_4$ and filtered, and the solvent removed. Purification by means of flash chromatography (SiO_2, 40:1, then 20:1 hexanes/EtOAc) afforded the respective iodoalkyne (2.67 g, 92%) as a yellow oil. TLC R_f = 0.69 (10:1 hexanes/EtOAc); $[\alpha]_D^{25}$ –10.7 (*c* 1.06, $CHCl_3$); ^{1}H NMR (300 MHz, $CDCl_3$) δ 3.49 (d, *J* = 6.5 Hz, 1H), 3.48 (dd, *J* = 9.4, 5.4 Hz, 1H), 3.30 (dd, *J* = 9.7, 6.8 Hz, 1H), 2.75 (ddd, *J* = 12.7, 7.1, 7.1 Hz, 1H), 1.79 (ddd, *J* = 13.9, 6.9, 2.7 Hz, 1H), 1.71–1.60 (m, 1H), 1.42 (ddd, *J* = 13.5, 6.6, 6.6 Hz, 1H), 1.15 (d, *J* = 7.1 Hz, 3H), 0.97–0.93 (m, 1H), 0.91 (s, 9H), 0.90 (s, 9H), 0.87 (d, *J* = 7.2 Hz, 3H), 0.85 (d, *J* = 6.7 Hz, 3H), 0.09 (s, 3H), 0.06 (s, 3H), 0.04 (s, 6H) ppm; ^{13}C NMR (75 MHz, $CDCl_3$) δ 97.9, 77.5, 68.2, 38.7, 34.2, 33.3, 33.2, 26.0, 26.0, 18.4, 18.4, 17.7, 17.3, 15.1, –3.9, –4.0, –5.3, –5.3 ppm; IR (film) 2955, 2930, 2857, 1468 cm^{-1}; HRMS (ES+) calcd for $C_{23}H_{47}O_2Si_2NaI$ $[M + Na]^+$ 561.2057, found 561.2069.

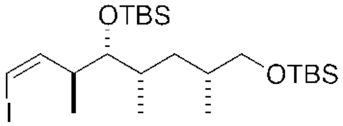

(5*R*,6*S*,8*R*)-5-((*S*,*Z*)-4-Iodobut-3-en-2-yl)-2,2,3,3,6,8,11,11,12,12-decamethyl-4,10-dioxa-3,11-disilatridecane (19). A solution of the iodoalkyne prepared above (2.26 g, 4.19 mmol) in THF (14 mL) and iPrOH (14 mL) was treated with TEA (2.92 mL, 20.95 mmol) and NBSH (4.55 g, 20.95 mmol) at rt. The resulting orange mixture was stirred for 16 h, then quenched with water and diluted with ether. The organic layer was collected and the water layer extracted with CH_2Cl_2 (4 × 50 mL). The organic layers were combined, dried over $MgSO_4$ and filtered, and the solvent removed. Purification by flash chromatography (SiO_2, 35:1 hexanes/EtOAC) afforded

cis-iodoalkene **19** (2.12 g, 94%) as a yellow oil. TLC R_f = 0.69 (20:1 hexanes/EtOAc); $[\alpha]_D^{25}$ + 0.60 (*c* 0.99, $CHCl_3$); ^1H NMR (500 MHz, $CDCl_3$) δ 6.27 (dd, *J* = 8.7, 7.6 Hz, 1H), 6.11 (d, *J* = 7.3 Hz, 1H), 3.50 (dd, *J* = 9.8, 4.8 Hz, 1H), 3.47 (t, *J* = 3.6 Hz, 1H), 3.23 (dd, *J* = 9.6, 7.6 Hz, 1H), 2.67 (dddd, *J* = 16.5, 7.0, 7.0, 3.3 Hz, 1H), 1.65–1.61 (m, 2H), 1.38 (ddd, *J* = 13.3, 7.8, 5.0 Hz, 1H), 0.97 (d, *J* = 7.0 Hz, 3H), 0.91 (s, 9H), 0.90 (d, *J* = 6.8 Hz, 3H), 0.89 (s, 9H), 0.88 (d, *J* = 6.7 Hz, 3H), 0.88–0.85 (m, 1H), 0.07 (s, 3H), 0.06 (s, 3H), 0.03 (s, 6H) ppm; ^{13}C NMR (125 MHz, $CDCl_3$) δ 144.2, 81.1, 79.0, 68.3, 43.3, 37.6, 35.8, 33.6, 26.1, 26.0, 18.4, 18.3, 17.8, 16.3, –3.6, –3.8, –5.3, –5.3 ppm; IR (neat) 2955, 2929, 2886, 2856, 1463, 1254 cm^{-1}; HRMS (EI) calcd for $C_{19}H_{40}O_2Si_2I$ [M – *t*-Bu]$^+$ 483.1611, found 483.1604.

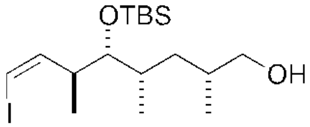

(2*R*,4*S*,5*R*,6*S*,*Z*)-5-((*tert*-Butyldimethylsilyl)oxy)-8-iodo-2,4,6-trimethyloct-7-en-1-ol. A solution of *cis*-iodoalkene **19** (2.11 g, 3.90 mmol) in THF (22 mL) at 0 °C was treated with a solution of HF-pyridine in pyridine/THF (15 mL, prepared in a polyethylene container by mixing 3 mL of HF-pyridine complex with 12 mL of pyridine, followed by dilution with 25 mL of THF) dropwise through an addition funnel. The reaction mixture was allowed to warm up slowly to rt and stirred for 24 h. The reaction mixture was recooled to 0 °C and quenched by the dropwise addition of saturated $NaHCO_3$ until no gas evolution was observed. The organic layer was collected and the water layer was extracted with CH_2Cl_2 (4 × 75 mL). The organic layers were combined, dried over $MgSO_4$ and filtered, and the solvent was removed. Purification by flash chromatography (SiO_2, 10:1, then 5:1 hexanes/EtOAc) afforded the respective primary alcohol (1.62 g, 97%) as a light yellow oil. TLC R_f = 0.30 (5:1 hexanes/EtOAc); $[\alpha]_D^{25}$ +32.5 (*c* 0.47, $CHCl_3$); ^1H NMR (300 MHz, $CDCl_3$) δ 6.29 (dd, *J* = 8.8, 7.4 Hz, 1H), 6.12 (d, *J* = 7.3 Hz, 1H), 3.53 (ddd, *J* = 10.4, 5.5, 5.5 Hz, 1H), 3.47 (t, *J* = 4.0 Hz, 1H), 3.33 (ddd, *J* = 10.6, 6.8, 5.6 Hz, 1H), 2.70 (dddd, *J* = 16.4, 7.0, 7.0, 3.0 Hz, 1H), 1.73–1.60 (m, 2H), 1.44 (ddd, *J* = 13.4, 8.0, 4.8 Hz, 1H), 1.22 (t, *J* = 5.8 Hz, 1H), 0.98 (d, *J* = 7.0 Hz, 3H), 0.94 (d, *J* = 6.7 Hz, 3H), 0.91 (d, *J* = 6.7 Hz, 3H), 0.91 (s, 9H), 0.07 (s, 3H), 0.06 (s, 3H) ppm; ^{13}C NMR (75 MHz, $CDCl_3$) δ 144.0, 81.2, 78.9, 67.6, 42.9, 36.9, 35.8, 33.3, 26.0, 18.3, 18.0, 17.9, 16.3, –3.6, –3.9 ppm; IR (film) 3360, 2955, 2930, 2857, 1774, 1461 cm^{-1}; HRMS (ESI) calcd for $C_{17}H_{35}O_2SiINa$ [M + Na]$^+$ 449.1343, found 449.1361.

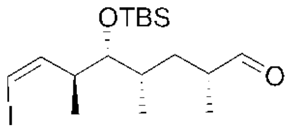

(2*R*,4*S*,5*R*,6*S*,*Z*)-5-((*tert*-Butyldimethylsilyl)oxy)-8-iodo-2,4,6-trimethyloct-7-enal (7). A solution of the primary alcohol prepared above (0.70 g, 1.65 mmol) in CH_2Cl_2 (7 mL) was treated with Dess-Martin periodinane (1.05 g, 2.48 mmol) and $NaHCO_3$ (278 mg, 3.30 mmol) at rt. After 1 h, the mixture was quenched with a saturated solution of $Na_2S_2O_3$ (7 mL). The organic layer was collected and the aqueous layer extracted with Et_2O (3 × 10 mL). The combined organic layers were dried over $MgSO_4$ and filtered, and the solvent was removed. Purification by short column chromatography (SiO_2, 20:1 hexanes/EtOAc) provided aldehyde **7** (0.57 g, 85%) as a light yellow oil. ^{1}H NMR (300 MHz, $CDCl_3$) δ 9.50 (d, *J* = 3.0 Hz, 1H), 6.32 (dd, *J* = 8.7, 7.2 Hz, 1H), 6.13 (d, *J* = 7.5 Hz, 1H), 3.52–3.49 (m, 1H), 2.74–2.66 (m, 1H), 2.43–2.35 (m, 1H), 1.94–1.83 (m, 1H), 1.68–1.52 (m, 1H), 1.20–1.16 (m, 1H), 1.08 (d, *J* = 6.9 Hz, 3H), 0.98 (d, *J* = 7.2 Hz, 3H), 0.92 (d, *J* = 6.6 Hz, 3H), 0.91 (s, 9H), 0.07 (s, 3H), 0.05 (s, 3H) ppm.

II. Scheme S1: Alternate route to the synthesis of middle fragment 7

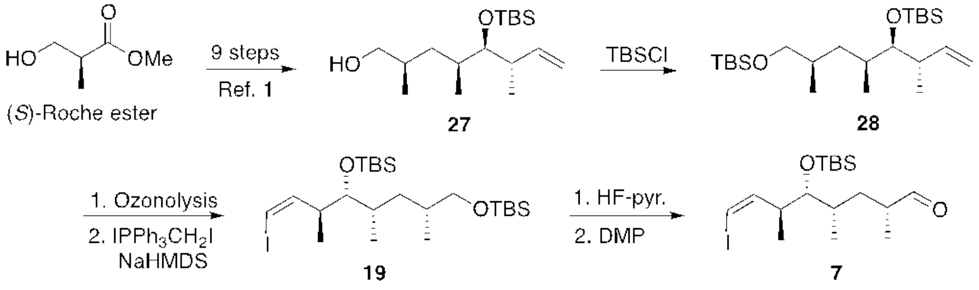

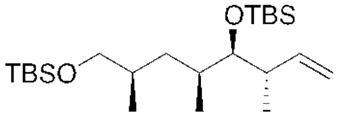

(5*R*,6*S*,8*R*)-5-((*S*)-But-3-en-2-yl)-2,2,3,3,6,8,11,11,12,12-decamethyl-4,10-dioxa-3,11-disilatridecane (28). A solution of alcohol **27**[1] (1.71 g, 5.71 mmol) in CH_2Cl_2 (10 mL) was treated successively with triethylamine (2.0 mL, 14.28 mmol), DMAP (70 mg, 0.57 mmol) and TBSCl (1.30 g, 8.57 mmol) at 0 °C. After 1 h, the reaction was quenched with saturated $NaHCO_3$ (10 mL). The mixture was extracted with CH_2Cl_2 (3 × 10 mL), the combined organic layers were washed with brine, dried over $MgSO_4$ and filtered, and the solvent was removed. Purification by

1) Ph.D. thesis of W.-H. Jung, University of Pittsburgh, 2008: http://etd.library.pitt.edu/ETD/available/etd-03252008-125826/

column chromatography (SiO_2, 30:1 hexanes/EtOAc) provided olefin **28** (2.06 g, 87%) as a colorless oil. ^1H NMR (300 MHz, $CDCl_3$) δ 5.85 (ddd, *J* = 17.1, 10.2, 7.5 Hz, 1H), 5.00–4.94 (m, 2H), 3.48 (dd, *J* = 9.6, 4.8 Hz, 1H), 3.36 (t, *J* = 4.1 Hz, 1H), 3.27 (dd, *J* = 9.6, 6.9 Hz, 1H), 2.34 (ddd, *J* = 18.9, 6.9, 6.9 Hz, 1H), 1.72–1.57 (m, 2H), 1.42–1.33 (m, 1H), 1.00 (d, *J* = 6.9 Hz, 3H), 0.90 (s, 9H), 0.89 (s, 9H), 0.90–0.89 (m, 1H), 0.88 (d, *J* = 6.9 Hz, 3H), 0.85 (d, *J* = 6.6 Hz, 3H), 0.05 (s, 6H), 0.04 (s, 3H), 0.04 (s, 3H) ppm; ^{13}C NMR (75 MHz, $CDCl_3$) δ 142.3, 113.8, 79.3, 68.2, 42.9, 38.7, 34.1, 33.5, 26.2, 26.0, 18.5, 18.4, 18.0, 17.6, 15.7, –3.7, –5.3 ppm.

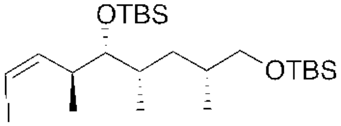

(5*R*,6*S*,8*R*)-5-((*S*,*Z*)-4-Iodobut-3-en-2-yl)-2,2,3,3,6,8,11,11,12,12-decamethyl-4,10-dioxa-3,11-disilatridecane (19).

Ozonolysis: Through a solution of olefin **28** (1.00 g, 2.41 mmol) and one crystal of Sudan III in 30 mL of 4:1 CH_2Cl_2/MeOH, a stream of ozone was bubbled at –78 °C until the pink solution became colorless. The solution was treated with Ph_3P (0.78 g) and then warmed slowly to rt over 2 h. The solvent was removed and the residue was purified by flash chromatography (SiO_2, 30:1 hexanes/EtOAc) to provide the aldehyde containing some inseparable Ph_3P.

Wittig reaction: A suspension of (iodomethyl)triphenylphosphonium iodide (2.00 g, 3.70 mmol) in dry THF (8 mL) was treated with NaHMDS (1.0 M, 3.70 mL, 3.70 mmol), and the slurry was stirred at rt for 30 min. After the dark red mixture was cooled to –78 ^oC, dry HMPA (1.30 mL) was added, followed by a solution of the aldehyde prepared as above in THF (4 mL). After the reaction mixture was warmed to rt over 1 h, it was stirred for another 30 min before being diluted with hexanes, filtered through silica gel, and concentrated in vacuo. Purification by means of flash chromatography (SiO_2, 40:1 hexanes/EtOAc) provided vinyl iodide **19** (1.10 g, 85% over two steps). Analytical data was consistent with the previously reported method.

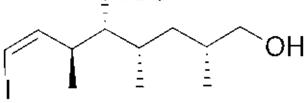

(2*R*,4*S*,5*R*,6*S*,*Z*)-5-((*tert*-Butyldimethylsilyl)oxy)-8-iodo-2,4,6-trimethyloct-7-en-1-ol. A solution of vinyl iodide **19** (1.05 g, 1.94 mmol) in THF (10 mL) at 0 ^oC was treated with a solution of HF-pyridine (3.3 mL) in pyridine/THF (12 mL/24 mL) dropwise by syringe. The reaction mixture was warmed to rt and stirred for 8 h. After quenching at 0 °C by the dropwise

addition of a saturated solution of $NaHCO_3$ until no CO_2 evolved, the mixture was extracted with EtOAc (3 × 20 mL). The combined organic layers were washed successively with a saturated solution of $CuSO_4$ (2 × 20 mL) and brine. The organic layer was dried over $MgSO_4$, filtered and the solvent was removed. Purification by column chromatography (SiO_2, 5:1 hexanes/EtOAc) provided the desired primary alcohol (0.70 g, 85%) as a colorless oil. Analytical data was consistent with the previously reported method.

III. Coupling of the top and middle fragments

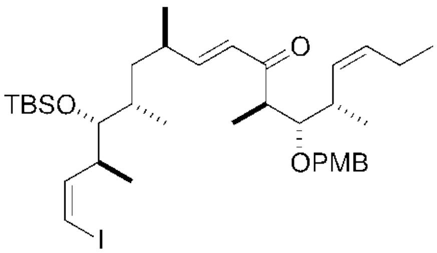

(3*Z*,5*S*,6*S*,7*R*,9*E*,11*R*,13*S*,14*R*,15*S*,16*Z*)-14-((*tert*-Butyldimethylsilyl)oxy)-17-iodo-6-((4-methoxybenzyl)oxy)-5,7,11,13,15-pentamethylheptadeca-3,9,16-trien-8-one (22). A solution of the ketophosphonate **8** (1.11 g, 2.70 mmol) in THF (6.75 mL) was treated with $Ba(OH)_2$ (852 mg, 2.70 mmol; activated by heating in the oven at 140 °C for 24 h prior to use) and the resulting reaction mixture was stirred at rt for 30 min. A solution of aldehyde **7** (1.15 g, 2.70 mmol) in wet THF (6.75 mL, 40:1 THF/water) was added dropwise by pipet and the resulting light yellow mixture was stirred for 24 h. After dissolving with brine and ether, the organic layer was collected. The water layer was extracted with CH_2Cl_2 (4 × 50 mL), the combined organic layers were dried over $MgSO_4$ and filtered, and the solvent was removed. Purification by flash chromatography (SiO_2, 15:1 hexanes/EtOAc) afforded enone **22** (1.55 g, 80%) as a yellow oil. TLC R_f = 0.29 (10:1 hexanes/EtOAc); $[\alpha]_D^{25}$ +23.3 (*c* 1.24, $CHCl_3$); ^{1}H NMR (300 MHz, $CDCl_3$) δ 7.27 (d, *J* = 8.0 Hz, 2H), 6.86 (d, *J* = 8.6 Hz, 2H), 6.65 (dd, *J* = 15.6, 8.6 Hz, 1H), 6.29 (dd, *J* = 8.8, 7.4 Hz, 1H), 6.11 (d, *J* = 7.3 Hz, 1H), 6.06 (d, *J* = 15.8 Hz, 1H), 5.43–5.31 (m, 2H), 4.55 (d, *J* = 10.7 Hz, 1H), 4.51 (d, *J* = 10.6 Hz, 1H), 3.80 (s, 3H), 3.64 (dd, *J* = 8.1, 3.4 Hz, 1H), 3.45 (dd, *J* = 4.0, 2.7 Hz, 1H), 2.90 (t, *J* = 7.4 Hz, 1H), 2.72–2.66 (m, 1H), 2.64–2.58 (m, 1H), 2.34 (bs, 1H), 1.98–1.81 (m, 2H), 1.51 (t, *J* = 10.2 Hz, 2H), 1.18 (d, *J* = 7.0 Hz, 3H), 1.15–1.11 (m, 1H), 1.04 (d, *J* = 6.7 Hz, 3H), 1.02 (d, *J* = 6.5 Hz, 3H), 0.96 (d, *J* = 7.0 Hz, 3H), 0.91 (s, 9H), 0.88 (t, *J* = 7.0 Hz, 3H), 0.82 (d, *J* = 6.7 Hz, 3H), 0.05 (s, 3H), 0.01 (s, 3H) ppm; ^{13}C NMR (75 MHz, $CDCl_3$) δ 202.8, 159.0, 152.4, 143.9, 131.9, 130.9, 130.5, 129.2, 127.8, 113.6, 84.0, 81.1, 79.1,

75.0, 55.4, 48.2, 42.4, 39.0, 36.4, 35.7, 34.6, 26.0, 20.8, 20.7, 19.0, 18.3, 18.1, 15.6, 14.4, 14.2, –3.7, –4.0 ppm; IR (film) 2529, 2929, 2856, 1622, 1461, 1250, 1072 cm^{-1}; HRMS (ESI) calcd for $C_{36}H_{59}O_4SiINa$ [M + Na]$^+$ 733.3120, found 733.3138.

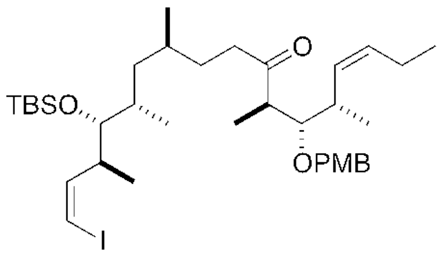

(3*Z*,5*S*,6*S*,7*R*,11*S*,13*S*,14*R*,15*S*,16*Z*)-14-((*tert*-Butyldimethylsilyl)oxy)-17-iodo-6-((4-methoxybenzyl)oxy)-5,7,11,13,15-pentamethylheptadeca-3,16-dien-8-one. Stryker's reagent (431.7 mg, 0.21 mmol) was transferred into a round-bottom flask inside a glove box followed by the addition of degassed toluene (14 mL). The solution was cooled to 0 °C and a solution of enone **22** (253 mg, 0.36 mmol) in degassed toluene (4 mL) was added dropwise by syringe. The resulting dark orange solution was stirred at 0 °C for 1 h, then allowed to warm up to rt and stirred for another hour. A few drops of water were added, the reaction mixture was exposed to air and stirring was continued overnight. After filtering through celite using ether as eluent, the solvent was removed and the product purified by flash chromatography (SiO_2, 90:1, 80:1, 60:1 and 10:1 hexanes/EtOAc) to afford the desired ketone (242 mg, 95%) as a colorless oil. TLC R_f = 0.51 (10:1 hexanes/EtOAc); $[\alpha]_D^{25}$ + 25.9 (*c* 0.86, $CHCl_3$); ^1H NMR (400 MHz, $CDCl_3$) δ 7.25 (d, *J* = 8.4 Hz, 2H), 6.86 (d, *J* = 8.6 Hz, 2H), 6.28 (dd, *J* = 8.7, 7.4 Hz, 1H), 6.11 (d, *J* = 7.2 Hz, 1H), 5.43–5.33 (m, 2H), 4.54 (d, *J* = 10.6 Hz, 1H), 4.47 (d, *J* = 10.6 Hz, 1H), 3.79 (s, 3H), 3.59 (dd, *J* = 7.7, 3.8 Hz, 1H), 3.46 (t, *J* = 3.5 Hz, 1H), 2.73–2.66 (m, 2H), 2.62–2.57 (m, 1H), 2.43–2.38 (m, 2H), 2.00–1.95 (m, 1H), 1.93–1.83 (m, 1H), 1.72–1.63 (m, 2H), 1.45–1.37 (m, 1H), 1.31 (ddd, *J* = 13.2, 8.4, 4.6 Hz, 1H), 1.16 (d, *J* = 7.1 Hz, 3H), 1.14–1.06 (m, 2H), 1.02 (d, *J* = 6.9 Hz, 3H), 0.96 (d, *J* = 6.9 Hz, 3H), 0.92 (t, *J* = 7.4 Hz, 3H), 0.91 (s, 9H), 0.87 (d, *J* = 6.7 Hz, 3H), 0.82 (d, *J* = 6.5 Hz, 3H), 0.07 (s, 3H), 0.06 (s, 3H) ppm; ^{13}C NMR (100 MHz, $CDCl_3$) δ 214.1, 159.1, 144.1, 132.1, 130.9, 130.5, 129.2, 113.6, 83.6, 81.1, 79.1, 74.8, 55.2, 50.1, 43.0, 40.8, 39.9, 35.6, 35.6, 29.8, 29.3, 26.1, 20.8, 20.4, 19.0, 18.3, 17.9, 16.1, 14.3, 13.6, –3.6, –3.9 ppm; IR (film) 2959, 2930, 2857, 1710, 1514, 1460, 1250, 1068 cm^{-1}; HRMS (EI) calcd for $C_{36}H_{61}O_4SiI$ [M]$^+$ 712.3384, found 712.3370.

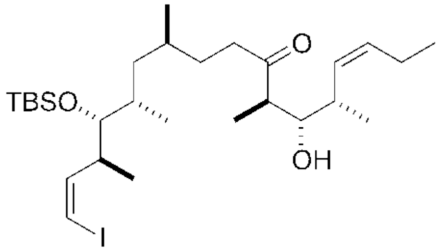

(3*Z*,5*S*,6*S*,7*R*,11*S*,13*S*,14*R*,15*S*,16*Z*)-14-((*tert*-Butyldimethylsilyl)oxy)-6-hydroxy-17-iodo-5,7,11,13,15-pentamethylheptadeca-3,16-dien-8-one (23). A solution of the ketone obtained above (247 mg, 0.34 mmol) in CH_2Cl_2 (34.6 mL) and buffer 7 (0.3 mL) was treated with DDQ (120 mg, 0.52 mmol) at 0 °C in one portion. The resulting brown-orange solution was stirred until the reaction was judged to be complete by TLC analysis (~4 h). The solution was quenched with saturated $NaHCO_3$ and stirring was continued for 30 min. After extraction with CH_2Cl_2 (4 × 30 mL) the combined organic layers were dried over $MgSO_4$and filtered, and the solvent was removed. Purification by means of flash chromatography (SiO_2, 10:1 hexanes/EtOAc) afforded alcohol **23** (159 mg, 78%) as a clear oil containing some inseparable PMB-aldehyde. This material was repurified for complete characterization. TLC R_f = 0.16 (10:1 hexanes/EtOAc); $[\alpha]_D^{25}$ +21.4 (*c* 0.705, CH_2Cl_2); ^{1}H NMR (500 MHz, $CDCl_3$) δ 6.29 (dd, *J* = 8.9, 7.4Hz, 1H), 6.11 (d, *J* = 7.3 Hz, 1H), 5.53 (ddd, *J* = 10.8, 7.4, 7.4 Hz, 1H), 5.26 (ddd, *J* = 11.1, 11.1, 1.3 Hz, 1H), 3.68 (ddd, *J* = 7.8, 3.3, 2.9 Hz, 1H), 3.47 (t, *J* = 4.0 Hz, 1H), 2.70 (dd, *J* = 8.7, 3.3 Hz, 1H), 2.67 (dd, *J* = 7.2, 3.5 Hz, 1H), 2.60–2.55 (m, 1H), 2.49 (dd, *J* = 5.8, 3.3 Hz, 1H), 2.47 (dd, *J* = 5.2, 3.6 Hz, 1H), 2.28 (d, *J* = 2.5 Hz, 1H), 2.10–2.02 (m, 2H), 1.71–1.61 (m, 2H), 1.49–1.43 (m, 1H), 1.33 (ddd, *J* = 13.4, 8.4, 4.7 Hz, 1H), 1.24–1.18 (m, 1H), 1.15 (d, *J* = 7.1 Hz, 3H), 0.98 (d, *J* = 7.5 Hz, 6H), 0.95–0.90 (m, 1H), 0.94 (d, *J* = 6.8 Hz, 3H), 0.91 (s, 9H), 0.88 (d, *J* = 6.8 Hz, 3H), 0.85 (d, *J* = 6.5 Hz, 3H), 0.07 (s, 3H), 0.06 (s, 3H) ppm; ^{13}C NMR (75 MHz, $CDCl_3$) δ 214.7, 144.0, 133.6, 130.9, 81.1, 79.1, 74.8, 47.9, 42.9, 40.6, 38.7, 35.6, 35.0, 29.7, 29.3, 26.1, 20.9, 20.3, 18.3, 17.9, 17.4, 16.0, 14.3, 9.6, –3.7, –3.9 ppm; IR (film) 3503, 2959, 1706, 1605, 1459, 1405, 1255 cm^{-1}; HRMS (ES+) calcd for $C_{28}H_{53}O_3NaSiI$ $[M + Na]^+$ 615.2706, found 615.2711.

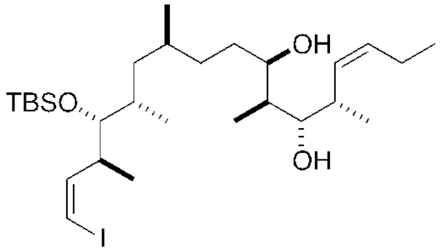

(3*Z*,5*S*,6*S*,7*S*,8*R*,11*S*,13*S*,14*R*,15*S*,16*Z*)-14-((*tert*-Butyldimethylsilyl)oxy)-17-iodo-5,7,11,13,15-pentamethylheptadeca-3,16-diene-6,8-diol. A solution of ketone **23** (40.6 mg, 0.069 mmol) in THF (1.0 mL) and MeOH (0.2 mL) at –78 °C was treated with Et_2BOMe (0.21 mL, 0.21 mmol, 1.0 M solution in THF) dropwise over 2 min. After 1 h $NaBH_4$ (6 mg, 0.16 mmol) was added. The mixture was stirred at –78 °C for 6 h and then quenched by the dropwise addition of acetic acid (0.3 mL). Water (5 mL) was added and the mixture was extracted with diethyl ether (4 × 5 mL). The combined organic layers were washed with brine, dried over $MgSO_4$ and filtered, and the solvent was removed. The residue was taken up in a 1.0 M solution of NaOAc in MeOH (5 mL) and H_2O (0.55 mL), to which 30% H_2O_2 (0.41 mL) was added. After stirring at rt for 2 h, the mixture was diluted with H_2O (5 mL) and extracted with diethyl ether (4 × 5 mL), dried over $MgSO_4$, and concentrated. Purification by column chromatography (SiO_2, 4:1 hexanes/EtOAc) provided the desired diol (37 mg, 90%) as a colorless oil. TLC R_f = 0.25 (5:1 hexanes/EtOAc); $[\alpha]_D^{25}$ +14.3 (*c* 1.5, $CHCl_3$). ^1H NMR (500 MHz, $CDCl_3$) δ 6.29 (dd, *J* = 9.0, 7.5 Hz, 1H), 6.10 (d, *J* = 7.0 Hz, 1H), 5.61 (dt, *J* = 11.5, 7.0 Hz, 1H), 5.13 (t, *J* = 10.2 Hz, 1H), 3.76 (t, *J* = 5.7 Hz, 1H), 3.46 (t, *J* = 3.7 Hz, 1H), 3.43 (bs, 1H), 3.39 (d, *J* = 10.0 Hz, 1H), 2.71–2.62 (m, 2H), 2.22 (bs, 1H), 2.14–2.06 (m, 2H), 1.70 (q, *J* = 7.0 Hz, 1H), 1.66–1.62 (m, 1H), 1.52–1.45 (m, 4H), 1.33 (ddd, *J* = 13.5, 8.5, 4.5 Hz, 1H), 0.99 (d, *J* = 7.5 Hz, 3H), 0.98 (d, *J* = 7.0 Hz, 3H), 0.92 (t, *J* = 6.5 Hz, 3H), 0.92 (s, 9H), 0.90 (d, *J* = 6.0 Hz, 3H), 0.89 (d, *J* = 6.5 Hz, 3H), 0.87 (d, *J* = 7.0 Hz, 3H), 0.93–0.87 (m, 1H), 0.84–0.82 (m, 1H), 0.07 (s, 3H), 0.06 (s, 3H) ppm; ^{13}C NMR (150 MHz, $CDCl_3$) δ 144.2, 135.3, 131.4, 81.1, 80.7, 79.3, 77.3, 43.1, 41.1, 36.7, 35.8, 35.7, 32.3, 32.2, 30.4, 26.2, 21.1, 20.7, 18.4, 18.0, 16.8, 16.2, 14.4, 4.1, –3.5, –3.8 ppm; IR (neat) 3381, 2960, 2930, 1461, 1073 cm^{-1}; HRMS (ESI) calcd for $C_{28}H_{55}O_3SiINa$ $[M + Na]^+$ 617.2857, found 617.2842.

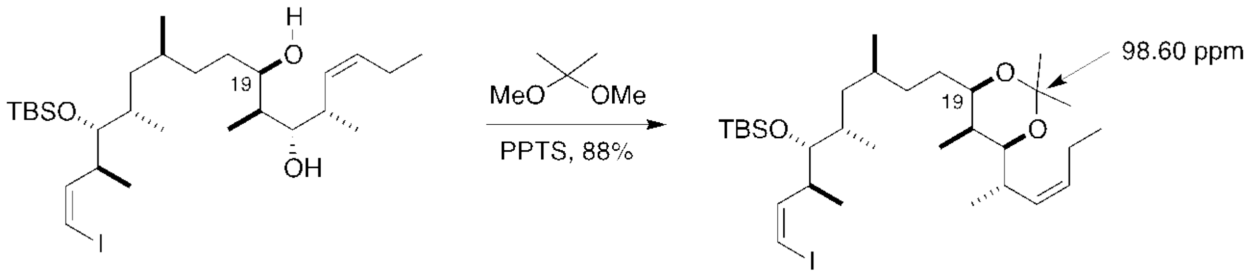

Acetonide: The diol prepared above (22.5 mg, 0.037 mmol) was treated with 2,2-dimethoxypropane (1.6 mL) and once completely dissolved the solution was cooled to 0 °C. PPTS (1.9 mg, 7.56 μmol) was added in one portion and the resulting reaction mixture was allowed to warm up slowly to rt and stirred until the reaction was judged to be complete by means of TLC analysis. The reaction mixture was diluted with ether and quenched with a saturated solution of $NaHCO_3$. The organic layer was collected and the water layer extracted with DCM (4 × 20 mL). The organic layers were combined, dried over $MgSO_4$ and filtered, and the solvent was removed. Purification by means of flash chromatography (SiO_2, 30:1 hexanes/EtOAc) afforded the desired acetonide (21.1 mg, 88%) as a clear oil. ^{1}H NMR (400 MHz, $CDCl_3$) δ 6.30 (dd, *J* = 8.4, 7.3 Hz, 1H), 6.10 (d, *J* = 7.3 Hz, 1H), 5.37–5.31 (m, 1H), 5.13 (dd, *J* = 10.5, 8.8 Hz, 1H), 3.75 (ddd, *J* = 6.1, 6.1, 1.7 Hz, 1H), 3.46–3.43 (m, 2H), 2.69 (dddd, *J* = 16.3, 7.0, 7.0, 2.8 Hz, 1H), 2.54 (dddd, *J* = 15.7, 7.0, 7.0, 7.0 Hz, 1H), 2.15–1.96 (m, 2H), 1.66–1.60 (m, 1H), 1.50–1.40 (m, 6H), 1.39–1.33 (m, 1H), 1.36 (s, 3H), 1.33 (s, 3H), 1.31–1.28 (m, 1H), 0.98 (d, *J* = 7.0 Hz, 3H), 0.95 (t, *J* = 7.5 Hz, 3H), 0.91 (s, 9H), 0.86 (d, *J* = 6.7 Hz, 6H), 0.84 (d, *J* = 6.7 Hz, 6H), 0.07 (s, 3H), 0.06 (s, 3H) ppm; ^{13}C NMR (75 MHz, $CDCl_3$) δ 144.2, 133.1, 131.7, 98.6, 81.0, 79.2, 77.5, 74.0, 42.8, 41.3, 35.8, 33.2, 32.2, 31.4, 30.4, 30.2, 30.0, 26.1, 21.0, 20.7, 19.5, 18.4, 18.1, 16.3, 16.1, 14.3, 4.5, –3.6, –3.8 ppm.

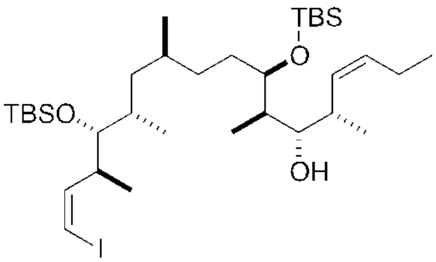

(3*Z*,5*S*,6*S*,7*R*,8*R*,11*S*,13*S*,14*R*,15*S*,16*Z*)-8,14-Bis((*tert*-butyldimethylsilyl)oxy)-17-iodo-5,7,11,13,15-pentamethylheptadeca-3,16-dien-6-ol (5). To a solution of the diol prepared above (36 mg, 0.061 mmol) in CH_2Cl_2 (1 mL) was added successively under stirring 2,6-lutidine (0.021 mL, 0.183 mmol) and TBSOTf (0.014 mL, 0.061 mmol) at –78 ^{o}C. After stirring for 1 h

the reaction mixture was quenched with a saturated solution of $NaHCO_3$ and extracted with CH_2Cl_2 (4 × 10 mL). The combined organic layers were washed with brine, dried over $MgSO_4$ and filtered, and the solvent was removed. The crude product was purified by flash chromatography (SiO_2, 40:1 hexanes/EtOAc) to give silyl ether **5** (37.2 mg, 86%) as a colorless oil. TLC R_f = 0.38 (5:1 hexanes/EtOAc); $[\alpha]_D^{25}$ +6.5 (*c* 1.23, $CHCl_3$); ^1H NMR (600 MHz, $CDCl_3$) δ 6.28 (dd, *J* = 8.4, 7.8 Hz, 1H), 6.11 (d, *J* = 7.2 Hz, 1H), 5.50 (dt, *J* = 10.8, 7.2 Hz, 1H), 5.28 (t, *J* = 10.5 Hz, 1H), 3.76–3.73 (m, 1H), 3.47 (t, *J* = 3.3 Hz, 1H), 3.40 (d, *J* = 8.4 Hz, 1H), 2.71–2.67 (m, 1H), 2.62 (dd, *J* = 16.8, 7.2 Hz, 1H), 2.28 (d, *J* = 1.8 Hz, 1H), 2.09 (td, *J* = 14.4, 7.2 Hz, 2H), 1.72–1.70 (m, 1H), 1.65–1.58 (m, 2H), 1.44–1.39 (m, 2H), 1.33–1.26 (m, 2H), 1.00 (d, *J* = 6.0 Hz, 3H), 0.98 (d, *J* = 6.5 Hz, 3H), 0.97–0.94 (m, 1H), 0.92 (t, *J* = 7.2 Hz, 3H), 0.92 (s, 9H), 0.90 (d, *J* = 7.0 Hz, 3H), 0.89 (s, 9H), 0.83–0.81 (m, 1H), 0.86 (d, *J* = 6.6 Hz, 6H), 0.08 (s, 6H), 0.06 (s, 3H), 0.05 (s, 3H) ppm; ^{13}C NMR (150 MHz, $CDCl_3$) δ 144.1, 132.9, 132.1, 81.2, 79.1, 77.3, 76.6, 43.2, 41.4, 37.5, 35.6, 35.5, 32.0, 31.1, 30.7, 26.2, 26.0, 21.0, 20.6, 18.4, 18.1, 17.9, 17.7, 16.0, 14.5, 7.1, –3.6, –3.7, –3.7, –4.4 ppm; IR (neat) 2957, 2930, 2857, 1461, 1254, 1075, 853 cm^{-1}; HRMS (ESI) calcd for $C_{34}H_{69}O_3Si_2INa$ $[M + Na]^+$ 731.3722, found 731.3750.

IV. Coupling with the bottom fragment:

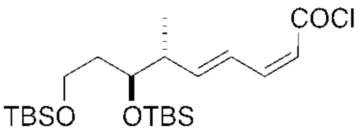

Synthesis of acid chloride 6a: The precursor carboxylic acid (150 mg, 0.35 mmol) was dissolved in toluene (3.5 mL) and the solution was treated at 0° C with Ghosez reagent (0.52 mmol, 72 μL). The resulting mixture was stirred at rt for 1.5 h and used as a crude for the coupling reaction.

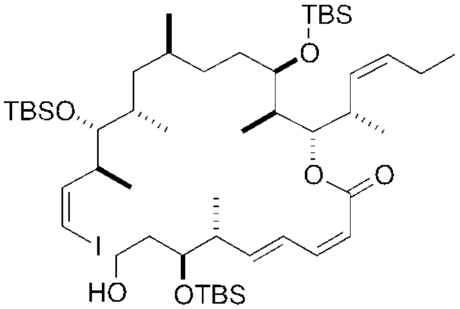

(2*Z*,4*E*,6*R*,7*S*)-(3*Z*,5*S*,6*S*,7*R*,8*R*,11*S*,13*S*,14*R*,15*S*,16*Z*)-8,14-Bis((*tert*-butyldimethylsilyl)oxy)-17-iodo-5,7,11,13,15-pentamethylheptadeca-3,16-dien-6-yl 7-((*tert*-butyldimethylsilyl)oxy)-9-hydroxy-6-methylnona-2,4-dienoate (25a).

Coupling of alcohol 5 with acid chloride 6a: A solution of alcohol **5** (92 mg, 0.129 mmol) in THF (1.3 mL) at –78 °C was treated with NaHMDS (0.258 mmol, 0.26 mL, 1 M solution in THF) under stirring and the resulting clear reaction mixture was stirred for 45 min. The crude acid chloride solution **6a** prepared above was added dropwise by syringe. The resulting orange solution was allowed to warm up slowly to rt and stirred for 2 h. The yellow reaction mixture was quenched with NH_4Cl and the organic layer was collected. The water layer was extracted with DCM (4 × 40 mL), the combined organic layers were dried over $MgSO_4$ and filtered, and the solvent was removed. This crude reaction mixture containing **24a** was immediately utilized for the next step without further purification.

Reaction with HF-pyridine: The crude product obtained above was dissolved in THF (1.3 mL) the solution was transferred to a polyethylene tube and cooled to 0 °C. HF-pyridine in pyridine and THF (0.75 mL, prepared by slowly adding 3 mL of HF-pyridine to 12 mL of pyridine and diluting with 25 mL of THF) was added dropwise by syringe and the resulting reaction mixture allowed to warm up slowly to rt and stirred overnight. The solution was recooled to 0°C followed by the addition of 0.5 mL of HF-pyridine in pyridine/THF and stirring was continued for 6 h. The reaction mixture was quenched at 0 °C by the dropwise addition of saturated $NaHCO_3$ until no more gas evolution was observed. The organic layer was collected and the water layer was extracted with DCM (4 × 30 mL). The organic layers were combined, dried over $MgSO_4$ and filtered, and the solvent was removed. Purification by means of flash chromatography (SiO_2, 20:1, 10:1 and 5:1 hexanes/EtOAc) afforded the titled compound **25a** (92 mg, 71% over 2 steps) as a yellow oil and recovered alcohol **5** (18 mg). TLC R_f = 0.15 (8:1 hexanes/EtOAc); $[\alpha]_D^{25}$ +36.6 (*c* 0.06, CH_2Cl_2); ^1H (300 MHz, $CDCl_3$) δ 7.36 (dd, *J* = 15.3, 11.3 Hz, 1H), 6.51 (t, *J* = 11.3 Hz, 1H), 6.26 (dd, *J* = 8.8, 7.4 Hz, 1H), 6.11 (d, *J* = 7.4 Hz, 1H), 5.96 (dd, *J* = 15.3, 8.0 Hz, 1H), 5.60 (d, *J* = 11.4 Hz, 1H), 5.41–5.28 (m, 2H), 5.03 (dd, *J* = 7.0, 4.6 Hz, 1H), 3.84 (ddd, *J* = 6.8, 4.7, 4.7 Hz, 1H), 3.75–3.68 (m, 2H), 3.60–3.56 (m, 1H), 3.45 (t, *J* = 3.7 Hz, 1H), 2.87–2.80 (m, 1H), 2.71–2.64 (m, 1H), 2.59–2.49 (m, 1H), 2.10–1.95 (m, 2H), 1.89–1.80 (m, 1H), 1.78–1.70 (m, 1H), 1.69–1.62 (m, 2H), 1.61–1.54 (m, 2H), 1.43–1.33 (m, 2H), 1.31–1.25 (m, 3H), 1.06 (d, *J* = 6.8 Hz, 3H), 0.97 (d, *J* = 6.7 Hz, 3H), 0.95 (t, *J* = 7.1 Hz, 3H), 0.92 (d, *J* = 6.9 Hz, 3H), 0.90 (s, 18H), 0.89 (s, 9H), 0.85 (d, *J* = 6.9 Hz, 3H), 0.84 (d, *J* = 7.0 Hz, 3H), 0.82 (d, *J* = 6.6 Hz, 3H), 0.09 (s, 3H), 0.07 (s, 9H), 0.04 (s, 6H) ppm; ^{13}C (75 MHz, $CDCl_3$) δ 166.0, 146.3, 144.6, 144.1, 131.9, 129.8, 127.1, 116.7, 81.1, 79.1, 77.2, 73.8, 72.6, 60.1, 43.3, 42.6, 41.4, 38.6, 35.4, 35.3, 33.8, 32.1, 31.1, 30.5, 26.1, 25.9, 25.8, 20.8, 20.4, 18.4, 18.2, 18.0, 17.9, 17.7, 15.8, 14.9, 14.4, 9.3, –3.6, –3.8, –3.9, –4.4, –4.5, –4.6 ppm; IR (neat) 3449,

2957, 2930, 2857, 1715, 1637, 1610, 1461, 1378, 1254, 1180 cm^{-1}; HRMS (ES+) calcd for $C_{50}H_{97}O_6NaSi_3I$ [M + Na]$^+$ 1027.5535, found 1027.5497.

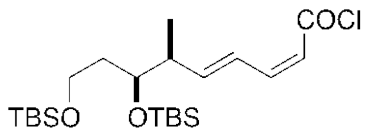

Synthesis of acid chloride 6b: Following the same procedure as explained before for the synthesis of **6a**, the acid (53 mg, 0.123 mmol) in dry toluene (0.5 mL) was treated with dimethyl(1-chloro-2-methyl-1-propenyl)-amine (Ghosez reagent) (0.020 mL, 0.148 mmol). The reaction mixture was stirred at rt for 1.5 h and the so-obtained solution of crude acid chloride **6b** was used directly in the next step.

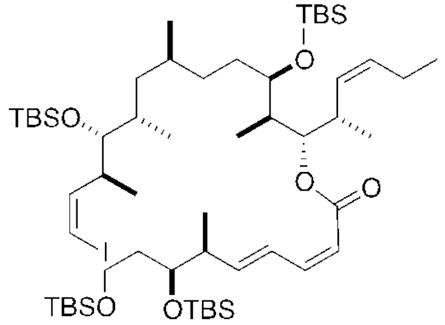

(2*Z*,4*E*,6*S*,7*S*)-(3*Z*,5*S*,6*S*,7*R*,8*R*,11*S*,13*S*,14*R*,15*S*,16*Z*)-8,14-bis((*tert*-butyldimethylsilyl)oxy)-17-iodo-5,7,11,13,15-pentamethylheptadeca-3,16-dien-6-yl 7,9-bis((*tert*-butyldimethylsilyl)-oxy)-6-methylnona-2,4-dienoate (24b). Alcohol **5** (67 mg, 0.095 mmol) in dry THF (1.2 mL) was treated with NaHMDS (0.104 mL, 0.104 mmol, 1.0 M in THF) at –78 °C. After the reaction mixture was stirred for 30 min, the crude acid chloride solution **6b** as prepared above was added dropwise by syringe. The resulting reaction mixture was stirred at rt for 1 h. The reaction was quenched with a saturated solution of $NaHCO_3$ (3 mL) and extracted with Et_2O (3 × 5 mL). The organic layers were combined, dried over $MgSO_4$ and filtered, and the solvent was removed. Purification by flash chromatography (40:1 hexanes/EtOAc) provided the ester **24b** (60 mg, 57%, 82% based on recovered alcohol) and the recovered alcohol **5** (20.0 mg). $[\alpha]_D^{25}$ +3.02 (*c* 0.53, $CHCl_3$); ^{1}H NMR (600 MHz, $CDCl_3$) δ 7.36 (dd, *J* = 15.0, 11.4 Hz, 1H), 6.54 (t, *J* = 11.1 Hz, 1H), 6.27 (t, *J* = 8.1 Hz, 1H), 6.13 (dd, *J* = 16.2, 7.2 Hz, 2H), 5.58 (d, *J* = 11.4 Hz, 1H), 5.36–5.33 (m, 2H), 5.03 (dd, *J* = 7.2, 4.2 Hz, 1H), 3.78–3.76 (m, 1H), 3.67–3.57 (m, 3H), 3.45 (t, *J* = 3.3 Hz, 1H), 2.87–2.83 (m, 1H), 2.71–2.66 (m, 1H), 2.50–2.45 (m, 1H), 2.09–1.97 (m, 2H), 1.74 (td, *J* = 6.6, 3.6 Hz, 1H), 1.66–1.60 (m, 2H), 1.55–1.51 (m, 2H), 1.45–1.33 (m, 2H), 1.31–1.21 (m, 3H), 1.03 (d, *J* = 7.2 Hz, 3H), 0.97 (d, *J* = 7.2 Hz, 3H), 0.96 (d, *J* = 7.8 Hz, 3H), 0.92 (d, *J* =

6.6 Hz, 3H), 0.92 (s, 18H), 0.89 (s, 18H), 0.93-0.89 (m, 1H), 0.85 (t, *J* = 6.6 Hz, 6H), 0.83 (d, *J* = 7.2 Hz, 3H), 0.08 (s, 3H), 0.07 (s, 3H), 0.06 (s, 3H), 0.05 (s, 3H), 0.04 (s, 12H) ppm; ^{13}C NMR (75 MHz, $CDCl_3$) δ 166.1, 147.4, 145.1, 144.2, 132.0, 130.0, 126.6, 116.2, 81.1, 79.1, 72.7, 72.6, 59.8, 53.4, 43.4, 42.4, 41.5, 38.7, 36.9, 35.4, 33.5, 32.2, 31.1, 30.5, 26.1, 25.9, 25.9, 25.9, 20.8, 20.4, 18.4, 18.2, 18.1, 18.1, 18.1, 17.7, 15.8, 14.5, 14.4, 9.4, –3.6, –3.7, –3.7, –4.4, –4.5, –4.5, –5.2, –5.3 ppm; IR (neat) 2956, 2930, 2857, 1715, 1637, 1462, 1254 cm^{-1}; HRMS (ESI) calcd for $C_{56}H_{111}IO_6Si_4Na$ $[M + Na]^+$ 1141.6400, found 1141.6404.

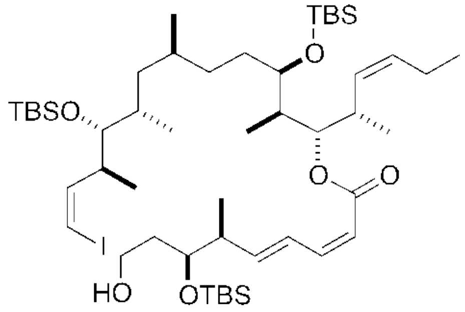

(2*Z*,4*E*,6*S*,7*S*)-(3*Z*,5*S*,6*S*,7*R*,8*R*,11*S*,13*S*,14*R*,15*S*,16*Z*)-8,14-Bis((*tert*-butyldimethylsilyl)oxy)-17-iodo-5,7,11,13,15-pentamethylheptadeca-3,16-dien-6-yl 7-((*tert*-butyldimethylsilyl)oxy)-9-hydroxy-6-methylnona-2,4-dienoate (25b). A solution of the *tetra*-substituted TBS-ether **24b** (57 mg, 0.051 mmol) in THF (0.36 mL) at 0 °C was treated with a solution of HF-pyridine (0.088 mL) in pyridine/THF (0.36 mL/0.72 mL). The reaction mixture was warmed to rt and stirred for 5 h. After quenching at 0 °C by slow addition of saturated $NaHCO_3$ until no CO_2 evolved, the mixture was extracted with EtOAc (3 × 4 mL). The combined organic layers were washed successively with a saturated solution of $CuSO_4$ (2 × 6 mL) and brine, then dried over $MgSO_4$ and filtered, and the solvent was removed. Purification by column chromatography (SiO_2, 15:1 hexanes/EtOAc) provided the desired primary alcohol **25b** (43 mg, 84%) as a colorless oil. $[\alpha]_D^{25}$ +0.28 (*c* 2.135, $CHCl_3$); ^{1}H NMR (600 MHz, $CDCl_3$) δ 7.38 (dd, *J* = 15.6, 10.8 Hz, 1H), 6.54 (t, *J* = 11.4 Hz, 1H), 6.26 (t, *J* = 8.1 Hz, 1H), 6.12–6.07 (m, 2H), 5.59 (d, *J* = 11.4 Hz, 1H), 5.37–5.31 (m, 2H), 5.03 (dd, *J* = 7.2, 4.8 Hz, 1H), 3.83–3.80 (m, 1H), 3.76–3.69 (m, 2H), 3.60–3.57 (m, 1H), 3.45 (t, *J* = 3.3 Hz, 1H), 2.85–2.82 (m, 1H), 2.69–2.66 (m, 1H), 2.57 (dd, *J* = 12.6, 6.0 Hz, 1H), 2.07–1.96 (m, 3H), 1.76–1.71 (m, 2H), 1.66–1.60 (m, 3H), 1.55–1.51 (m, 1H), 1.40–1.33 (m, 2H), 1.29–1.22 (m, 2H), 1.06 (d, *J* = 7.2 Hz, 3H), 0.97 (d, *J* = 6.6 Hz, 3H), 0.96 (d, *J* =7.8 Hz, 3H), 0.92 (t, *J* = 6.6 Hz, 3H), 0.91 (s, 18H), 0.90 (s, 9H), 0.86 (d, *J* = 6.6 Hz, 3H), 0.84 (d, *J* = 6.6 Hz, 3H), 0.83 (d, *J* = 6.6 Hz, 3H), 0.81-0.77 (m, 1H), 0.11 (s, 3H), 0.09 (s, 3H), 0.07 (s, 6H), 0.06 (s, 6H) ppm; ^{13}C NMR (75 MHz, $CDCl_3$) δ 166.1, 146.2, 144.8, 144.2, 132.0,

130.0, 127.0, 116.6, 81.2, 79.2, 74.5, 72.6, 60.2, 43.3, 42.4, 41.5, 38.7, 35.4, 35.4, 33.6, 32.2, 31.1, 30.5, 26.2, 26.0, 25.9, 20.8, 20.4, 18.4, 18.3, 18.1, 18.1, 18.0, 17.8, 15.8, 15.4, 14.5, 9.4, –3.6, –3.7, –3.7, –4.3, –4.5, –4.5 ppm; IR (neat) 2957, 2929, 2856, 1713, 1636, 1462, 1254 cm^{-1}.

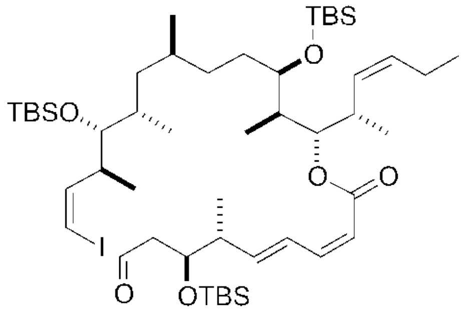

(2*Z*,4*E*,6*R*,7*S*)-(3*Z*,5*S*,6*S*,7*R*,8*R*,11*S*,13*S*,14*R*,15*S*,16*Z*)-8,14-Bis((*tert*-butyldimethylsilyl)oxy)-17-iodo-5,7,11,13,15-pentamethylheptadeca-3,16-dien-6-yl 7-((*tert*-butyldimethylsilyl)oxy)-6-methyl-9-oxonona-2,4-dienoate (4a). Alcohol **25a** (73 mg, 0.072 mmol) was dissolved in DCM (2.4 mL) and the solution treated with $NaHCO_3$ (9.1 mg, 0.11 mmol) and Dess-Martin periodinane (46 mg, 0.11 mmol) in one portion at rt. The reaction mixture was stirred for 1 h, then dissolved with water and quenched with a saturated solution of $Na_2S_2O_3$. This mixture was stirred until two layers were clearly visible. After extraction with DCM (4 × 25 mL), the combined organic layers were dried over $MgSO_4$ and filtered, and the solvent removed. Purification by means of flash chromatography (SiO_2, 10:1 hexanes/EtOAc) afforded aldehyde **4a** (69 mg, 95%) as a clear oil. TLC R_f = 0.47 (8:1 hexanes/EtOAc); ^{1}H NMR (400 MHz, $CDCl_3$) δ 9.77 (dd, *J* = 2.5, 1.7 Hz, 1H), 7.37 (dd, *J* = 15.4, 11.2 Hz, 1H), 6.51 (t, *J* = 11.4 Hz, 1H), 6.26 (dd, *J* = 8.8, 7.3 Hz, 1H), 6.11 (d, *J* = 7.3 Hz, 1H), 5.94 (dd, *J* = 15.4, 8.1 Hz, 1H), 5.62 (d, *J* = 11.4 Hz, 1H), 5.40–5.29 (m, 2H), 5.03 (dd, *J* = 7.1, 4.7 Hz, 1H), 4.21 (ddd, *J* = 6.8, 4.5, 4.5 Hz, 1H), 3.60–3.56 (m, 1H), 3.45 (t, *J* = 3.7 Hz, 1H), 2.84 (ddd, *J* = 8.8, 6.7, 4.7 Hz, 1H), 2.67 (ddd, *J* = 9.0, 7.0, 3.2 Hz, 1H), 2.54 (ddd, *J* = 16.3, 6.9, 2.6 Hz, 2H), 2.45 (ddd, *J* = 16.2, 4.7, 1.6 Hz, 1H), 2.11–1.95 (m, 2H), 1.74 (ddd, *J* = 6.8, 6.8, 3.8 Hz, 1H), 1.65–1.49 (m, 3H), 1.43–1.33 (m, 2H), 1.31–1.21 (m, 3H), 1.09 (d, *J* = 6.8 Hz, 3H), 0.97 (d, *J* = 7.1 Hz, 3H), 0.96 (t, *J* = 7.5 Hz, 3H), 0.92 (d, *J* = 7.3 Hz, 3H), 0.90 (s, 18H), 0.87 (s, 9H), 0.85 (d, *J* = 6.7 Hz, 3H), 0.84 (d, *J* = 7.1 Hz, 3H), 0.82 (d, *J* = 7.0 Hz, 3H), 0.08 (s, 3H), 0.07 (s, 6H), 0.05 (s, 6H), 0.04 (s, 3H) ppm; ^{13}C NMR (100 MHz, $CDCl_3$) δ 201.6, 166.0, 144.9, 144.4, 144.1, 132.0, 129.8, 127.8, 117.2, 81.1, 79.1, 77.2, 72.6, 70.8, 48.3, 43.3, 43.3, 41.4, 38.6, 35.4, 33.5, 32.2, 31.1, 30.5, 26.2, 25.9, 25.7, 20.8, 20.4, 18.3, 18.2, 18.1, 18.0, 17.8, 15.8, 15.1, 14.4, 9.3, –3.6, –3.7, –3.7, –4.5, –4.5, –4.6 ppm.

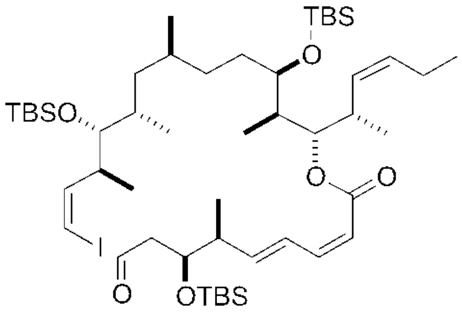

(2*Z*,4*E*,6*S*,7*S*)-(3*Z*,5*S*,6*S*,7*R*,8*R*,11*S*,13*S*,14*R*,15*S*,16*Z*)-8,14-Bis((*tert*-butyldimethylsilyl)oxy)-17-iodo-5,7,11,13,15-pentamethylheptadeca-3,16-dien-6-yl 7-((*tert*-butyldimethylsilyl)oxy)-6-methyl-9-oxonona-2,4-dienoate (4b). Following the same procedure utilized for aldehyde **4a,** a solution of alcohol **25b** (41 mg, 0.041 mmol) in CH_2Cl_2 (3 mL) was treated with DMP (0.026 g, 0.061 mmol) and $NaHCO_3$ (0.007 g, 0.082 mmol) at rt. After workup and purification by flash chromatography aldehyde **4b** (38.5 mg, 94%) was obtained as a light yellow oil. ^{1}H NMR (600 MHz, $CDCl_3$) δ 9.78 (t, *J* = 2.1 Hz, 1H), 7.40 (dd, *J* = 15.0, 10.8 Hz, 1H), 6.53 (t, *J* = 11.1 Hz, 1H), 6.26 (dd, *J* = 9.0, 7.2 Hz, 1H), 6.11 (d, *J* = 7.2 Hz, 1H), 6.04 (dd, *J* = 15.6, 7.2 Hz, 1H), 5.62 (d, *J* = 11.4 Hz, 1H), 5.38–5.31 (m, 2H), 5.03 (dd, *J* =7.8, 4.2 Hz, 1H), 4.18–4.16 (m, 1H), 3.59–3.57 (m, 1H), 3.45 (t, *J* = 3.6 Hz, 1H), 2.85–2.84 (m, 1H), 2.69–2.67 (m, 1H), 2.56–2.47 (m, 3H), 2.07–1.99 (m, 2H), 1.76–1.73 (m, 1H), 1.66 (d, *J* =6.0 Hz, 1H), 1.62 (dd, *J* = 7.2, 1.8 Hz, 1H), 1.54–1.52 (m, 1H), 1.42–1.33 (m, 2H), 1.29–1.22 (m, 3H), 1.07 (d, *J* = 6.6 Hz, 3H), 0.99–0.97 (m, 6H), 0.92 (s, 18H), 0.92 (d, *J* = 6.6 Hz, 3H), 0.88 (d, *J* = 6.6 Hz, 3H), 0.87 (s, 9H), 0.85 (t, *J* = 7.2 Hz, 3H), 0.83 (d, *J* = 6.6 Hz, 3H), 0.09 (s, 3H), 0.08 (s, 3H), 0.07 (s, 3H), 0.06 (s, 3H), 0.06 (s, 3H), 0.05 (s, 3H) ppm.

V. Intramolecular NHK reactions:

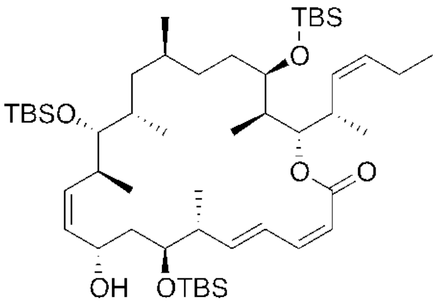

(3*Z*,5*E*,7*R*,8*S*,10*S*,11*Z*,13*S*,14*R*,15*S*,17*S*,20*R*,21*R*,22*S*)-8,14,20-Tris((*tert*-butyldimethylsilyl)oxy)-22-((*S*,*Z*)-hex-3-en-2-yl)-10-hydroxy-7,13,15,17,21-pentamethyloxacyclodocosa-3,5,11-trien-2-one (26a).

Procedure a: $CrCl_2$ (90 mg, 0.73 mmol), $NiCl_2$(dppf) (6.7 mg, 0.01 mmol) and 4,4'-di-*tert*-butyl-2,2'-dipyridyl (196 mg, 0.73 mmol) were transferred to a flask inside a glove box and dissolved in dry and degassed THF (24 mL). The resulting green solution was stirred at rt until completely homogeneous (~2 h). A solution of aldehyde **4a** (48.9 mg, 0.048 mmol) in THF (25 mL) was added dropwise by syringe and stirring was continued at rt for 3 days. The reaction was quenched with sodium serinate (5 mL, 1 M) and the resulting brown solution was stirred at rt for 2 h. The reaction mixture was extracted with EtOAc (4 × 20 mL), the organic layers were combined, dried over $MgSO_4$ and filtered, and the solvent was removed. After filtration through a plug of silica gel with 1:1 hexanes/EtOAc as eluent, the solvent was removed and the residue was purified by means of preparative TLC (8:1 hexanes/EtOAc, 2×) to afford macrolactone **26a** (9.2 mg, 22%) as a light yellow oil.

Procedure b (optimized conditions): $CrCl_2$ (136 mg, 1.11 mmol) and $NiCl_2$ (2.0 mg, 0.015 mmol) were transferred to a flask inside a glove box and dissolved in freshly distilled, dry and degassed DMF (6.5 mL). The resulting green solution was stirred until completely homogeneous (~30 min). A solution of aldehyde **4a** (74 mg, 0.073 mmol) in freshly distilled and degassed THF (8.5 mL) was added dropwise by syringe and the resulting reaction mixture was stirred at rt overnight. The reaction was quenched with sodium serinate (2 mL, 1 M) and the resulting solution was stirred for 1 h. The product was extracted with ether (2 × 20 mL) and EtOAc (2 × 20 mL). The organic layers were combined, dried over $MgSO_4$ and filtered, and the solvent was removed. Purification by flash chromatography (SiO_2, 25:1, 20:1 and 15:1 hexanes/EtOAc) afforded macrolactone **26a** (31 mg, 48%) as a yellow oil. TLC R_f = 0.39 (5:1 hexanes/EtOAc); ^{1}H NMR (400 MHz, $CDCl_3$) δ 7.10 (t, *J* = 13.0 Hz, 1H), 6.51 (t, *J* = 11.2 Hz, 1H), 6.05 (dd, *J* = 15.6, 6.4 Hz, 1H), 5.58 (d, *J* = 11.2 Hz, 2H), 5.39–5.33 (m, 2H), 5.27 (t, *J* = 10.0 Hz, 1H), 5.09 (t,

J = 5.4 Hz, 1H), 4.56 (dd, J = 12.8, 8.0 Hz, 1H), 4.09–4.05 (m, 1H), 3.48 (bs, 1H), 3.26 (d, J = 2.6 Hz, 1H), 2.86–2.80 (m, 1H), 2.65 (t, J = 7.0 Hz, 1H), 2.54 (bs, 1H), 2.07–2.00 (m, 2H), 1.86–1.82 (m, 1H), 1.59–1.54 (m, 5H), 1.43–1.40 (m, 3H), 1.28–1.21 (m, 2H), 1.07 (d, J = 6.7 Hz, 3H), 1.00 (d, J = 7.1 Hz, 3H), 0.96 (d, J = 6.4 Hz, 3H), 0.94 (t, J = 7.6 Hz, 3H), 0.92 (s, 9H), 0.92 (s, 9H), 0.90 (s, 9H), 0.90 (d, J = 7.2 Hz, 3H), 0.80 (d, J = 6.6 Hz, 6H), 0.14 (s, 3H), 0.11 (s, 3H), 0.05 (s, 6H), 0.04 (s, 3H), 0.03 (s, 3H) ppm; IR (neat) 3477, 2957, 2930, 2857, 1712, 1640, 1596, 1462, 1254 cm^{-1}; LRMS (EI+) 900.65 $[M + Na]^+$.

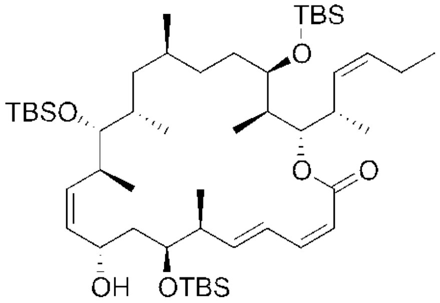

(3*Z*,5*E*,7*S*,8*S*,10*S*,11*Z*,13*S*,14*R*,15*S*,17*S*,20*R*,21*R*,22*S*)-8,14,20-Tris((*tert*-butyldimethylsilyl)oxy)-22-((*S*,*Z*)-hex-3-en-2-yl)-10-hydroxy-7,13,15,17,21-pentamethyloxacyclodocosa-3,5,11-trien-2-one (26b). Following procedure a, $CrCl_2$ (70 mg, 0.568 mmol), $NiCl_2$(dppf) (3.9 mg, 0.0057 mmol) and 4,4'-Di-*tert*-butyl-2,2'-dipyridyl (152 mg, 0.568 mmol) were combined inside a glove. Anhydrous, deoxygenated THF (28 mL) was added and the mixture was stirred at rt until a dark green homogeneous solution was formed (~2 h). The azeotropically dried (3 × 3 mL of dry toluene) aldehyde **4b** (38 mg, 0.038 mmol) was dissolved in anhydrous, deoxygenated THF (10 mL) and this solution was transferred into the flask containing the catalysts. After the reaction mixture was stirred at rt under argon atmosphere for 5 days, it was quenched with sodium serinate (30 mL, 1 M, prepared from sodium bicarbonate and *d,l*-serine) and stirred for another hour. The aqueous layer was extracted with EtOAc (3 × 25 mL), and the combined organic layers were dried over $MgSO_4$ and filtered, and the solvent was removed. Purification by preparative TLC (8:1 hexanes/EtOAc) provided macrolactone **26b** (14 mg, 42%) as a light yellow oil containing a few inseparable impurities. ^1H NMR (600 MHz, $CDCl_3$) δ 7.17 (dd, J = 15.0, 12.0 Hz, 1H), 6.49 (t, J = 11.4 Hz, 1H), 6.06 (dd, J = 15.0, 6.6 Hz, 1H), 5.57–5.53 (m, 2H), 5.35–5.28 (m, 2H), 5.26–5.20 (m, 2H), 4.62 (t, J = 9.0 Hz, 1H), 3.87 (t, J = 5.7 Hz, 1H), 3.40 (bs, 1H), 3.31–3.29 (m, 1H), 2.82–2.77 (m, 1H), 2.64–2.60 (m, 2H), 2.09–1.97 (m, 4H), 1.90–1.88 (m, 1H), 1.54–1.50 (m, 2H), 1.41–1.35 (m, 4H), 1.29–1.22 (m, 2H), 1.16–1.12 (m, 1H), 1.06 (d, J = 6.6 Hz, 3H), 1.00 (d, J = 7.2 Hz, 3H), 0.97 (d, J = 7.8 Hz, 3H),

0.96 (d, J = 6.6 Hz, 3H), 0.94 (t, J = 7.8 Hz, 3H), 0.93 (s, 9H), 0.91 (s, 18H), 0.78 (d, J = 6.0 Hz, 6H), 0.14 (s, 3H), 0.12 (s, 3H), 0.05 (s, 3H), 0.034 (s, 3H), 0.03(s, 3H), 0.02 (s, 3H) ppm.

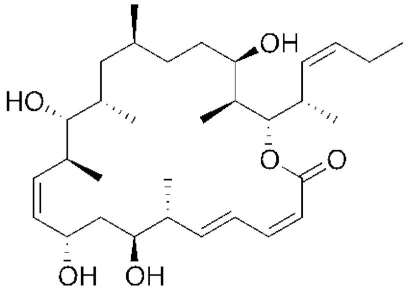

25,26-Dihydrodictyostatin (3a). A solution of macrolactone **26a** (7.6 mg, 0.008 mmol) in THF (1 mL) was transferred into a polyethylene tube and the solution was cooled to 0 °C. HF-pyridine (0.2 mL) was added and the resulting reaction mixture was allowed to warm up slowly to rt and stirred for 3 days. The reaction mixture was quenched at 0 °C by the dropwise addition of saturated $NaHCO_3$ and stirring was continued for 45 min. The reaction mixture was extracted with EtOAc (4 × 20 mL), the organic layers were combined, dried over $MgSO_4$ and filtered, and the solvent removed. Purification by means of preparative TLC (1:1 hexanes/EtOAc) afforded **3a** (4 mg, 86%) as a white solid. TLC R_f = 0.32 (1:2.5 hexanes/EtOAc); $[\alpha]_D^{25}$ –28.5 (*c* 0.2, MeOH); ^{1}H NMR (700 MHz, CD_3OD) δ 7.22 (dd, J = 15.6, 11.3 Hz, 1H), 6.38 (t, J = 11.3 Hz, 1H), 6.15 (dd, J = 15.6, 6.7 Hz, 1H), 5.54 (d, J = 11.3 Hz, 1H), 5.49 (t, J = 10.4 Hz, 1H), 5.40–5.35 (m, 2H), 5.21 (t, J = 10.2 Hz, 1H), 5.08 (dd, J = 7.1, 4.6 Hz, 1H), 4.62 (ddd, J = 9.4, 9.4, 3.0 Hz, 1H), 4.03 (ddd, J = 10.7, 3.0, 3.0 Hz, 1H), 3.32–3.28 (m, 1H), 3.07 (dd, J = 8.1, 3.0 Hz, 1H), 2.94 (ddd, J = 9.9, 6.7, 6.7 Hz, 1H), 2.75–2.70 (m, 1H), 2.56 (dd, J = 10.3, 6.5 Hz, 1H), 2.08 (dddd, J = 7.4, 7.4, 3.5, 1.3 Hz, 2H), 1.87–1.81 (m, 2H), 1.60–1.56 (m, 3H), 1.54–1.51 (m, 1H), 1.47 (ddd, J = 14.2, 10.9, 3.5 Hz, 1H), 1.39–1.35 (m, 2H), 1.25–1.21 (m, 2H), 1.12 (d, J = 6.9 Hz, 3H), 1.08 (d, J = 7.0 Hz, 3H), 1.03 (d, J = 6.9 Hz, 3H), 0.97 (t, J = 7.5 Hz, 3H), 0.94 (d, J = 6.7 Hz, 3H), 0.93 (d, J = 6.4 Hz, 3H), 0.90 (d, J = 6.7 Hz, 3H) ppm; ^{13}C (700 MHz, CD_3OD) δ 168.2, 146.7, 145.1, 135.1, 133.6, 132.0, 131.3, 128.7, 118.2, 80.5, 78.7, 74.1, 70.5, 65.6, 50.0, 44.2, 42.3, 41.0, 40.8, 35.9, 35.5, 33.0, 32.6, 31.4, 30.9, 22.0, 22.0, 19.5, 18.4, 16.2, 14.9, 10.6 ppm; IR (neat) 3397, 2924, 2855, 1705, 1634, 1458 cm^{-1}; HRMS (ES+) calcd for $C_{32}H_{54}O_6Na$ [M + Na]$^+$ 557.3818, found 557.3863.

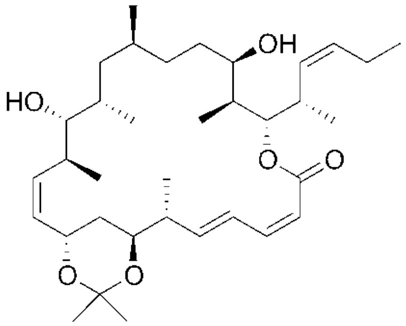

Acetonide. Macrolactone **3a** (3.2 mg, 5.9 µmol) was dissolved in acetone (3 drops) and the solution treated with 2,2-dimethoxypropane (1.4 µL, 0.012 mmol) and PPTS (1.2 mg,1.2 µmol) at rt. The resulting reaction mixture was stirred at rt for 12 h. The product was purified by preparative TLC (2:1 hexanes/EtOAc) to afford the desired acetonide (2.1 mg, 61%) as a white solid. ^{1}H NMR (700 MHz, $CDCl_3$) δ 7.38 (dd, *J* = 15.4, 11.2 Hz, 1H), 6.51 (t, *J* = 11.2 Hz, 1H), 6.02 (dd, *J* = 15.6, 6.0 Hz, 1H), 5.52 (d, *J* = 11.2 Hz, 1H), 5.44 (t, *J* = 10.7 Hz, 1H), 5.39 (dd, *J* = 10.7, 8.8 Hz, 1H), 5.34 (ddd, *J* = 10.7, 7.4, 7.2 Hz, 1H), 5.15 (t, *J* = 10.2 Hz, 1H), 4.97 (dd, *J* = 8.8, 1.9 Hz, 1H), 4.52 (ddd, *J* = 10.4, 10.4, 4.6 Hz, 1H), 4.00 (ddd, *J* = 10.7, 10.7, 3.4 Hz, 1H), 3.31 (t, *J* = 7.1 Hz, 1H), 3.17 (t, *J* = 5.7 Hz, 1H), 2.82–2.76 (m, 1H), 2.70–2.64 (m, 2H), 2.07–2.02 (m, 1H), 2.01–1.97 (m, 1H), 1.91–1.87 (m, 1H), 1.78–1.75 (m, 2H), 1.71–1.67 (m, 2H), 1.51–1.44 (m, 3H), 1.40 (s, 6H), 1.38–1.33 (m, 2H), 1.23–1.20 (m, 1H), 1.18 (d, *J* = 6.8 Hz, 3H), 1.04 (d, *J* = 6.8 Hz, 3H), 0.98 (d, *J* = 6.8 Hz, 3H), 0.94 (d, *J* = 6.8 Hz, 3H), 0.93 (d, *J* = 7.0 Hz, 3H), 0.93 (t, *J* = 7.0 Hz, 3H), 0.92 (d, *J* = 6.7 Hz, 3H) ppm; ^{13}C NMR (700 MHz, $CDCl_3$) δ 166.2, 144.7, 144.4, 133.7, 132.4, 131.2, 130.0, 127.4, 116.6, 100.1, 76.1, 75.9, 73.6, 67.4, 64.3, 40.4, 40.3, 39.6, 35.3, 34.5, 32.4, 32.2, 32.0, 31.8, 29.6, 28.9, 26.4, 24.8, 21.4, 20.7, 17.8, 17.4, 14.5, 14.4, 10.0 ppm.

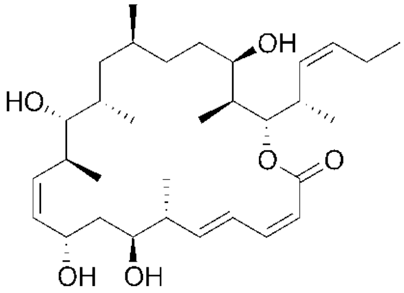

6-*Epi*-25,26-dihydrodictyostatin (3b). Following the same procedure as described above, macrolactone **26b** (8.7 mg, 0.010 mmol) in THF (1.5 mL) was treated with HF-pyridine (0.160 mL) at 0 °C and stirred at rt for 24 h. After workup and purification by means of preparative TLC (1:5 hexanes/EtOAc), the final product **3b** (4.4 mg, 82%) was obtained as a white solid, which was further subjected to HPLC (IPA/Hexane 5:95, chiral whelk column) purification. $[\alpha]_D^{25}$ –43.0 (*c* 0.165, MeOH); ^{1}H NMR (600 MHz, CD_3OD) δ 7.28 (dd, *J* = 15.0, 11.4 Hz, 1H), 6.60 (t, *J* = 11.4 Hz, 1H), 5.81 (dd, *J* = 15.0, 11.2 Hz, 1H), 5.57 (d, *J* = 10.8 Hz, 1H), 5.48 (t, *J* = 9.9 Hz, 1H), 5.38 (dt, *J* = 10.8, 7.2 Hz, 1H), 5.32 (dd, *J* = 10.8, 9.0 Hz, 1H), 5.15 (t, *J* = 10.2 Hz, 1H), 5.04 (dd, *J* = 9.6, 3.0 Hz, 1H), 4.67 (t, *J* = 8.7 Hz, 1H), 3.60–3.56 (m, 1H), 3.03 (dd, *J* = 9.6, 3.0 Hz, 1H), 2.94–2.88 (m, 2H), 2.71–2.68 (m, 1H), 2.22–2.13 (m, 2H), 2.10–2.05 (m, 2H), 1.88 (td, *J* = 11.1, 2.4 Hz, 1H), 1.73–1.70 (m, 1H), 1.62–1.60 (m, 1H), 1.58–1.53 (m, 1H), 1.49–1.47 (m, 1H), 1.41–1.31 (m, 4H), 1.26–1.21 (m, 1H), 1.19 (d, *J* = 7.2 Hz, 3H), 1.10 (d, *J* = 7.2 Hz, 3H), 1.08 (d, *J* = 6.6 Hz, 3H), 1.00 (t, *J* = 7.2 Hz, 3H), 0.97 (d, *J* = 7.8 Hz, 3H), 0.96 (t, *J* = 6.0 Hz, 3H), 0.93–0.83 (m, 2H), 0.41 (qd, *J* = 12.6, 4.8 Hz, 1H) ppm;^{13}C NMR (150 MHz, CD_3OD) δ 165.9, 146.1, 143.7, 133.6, 131.9, 131.3, 128.5, 127.1, 116.2, 79.1, 76.0, 73.6, 70.1, 63.7, 46.8, 43.3, 40.5, 40.4, 33.9, 33.8, 33.7, 32.3, 31.2, 30.1, 20.8, 20.4, 17.8, 16.8, 16.1, 14.8, 13.4, 8.5 ppm; IR (neat) 3347, 2960, 2924, 1709, 1636, 1454, 1100, 1073 cm^{-1}; HRMS (ESI) calcd for $C_{32}H_{54}O_6K$ $[M + K]^+$ 573.3557, found 573.3565.

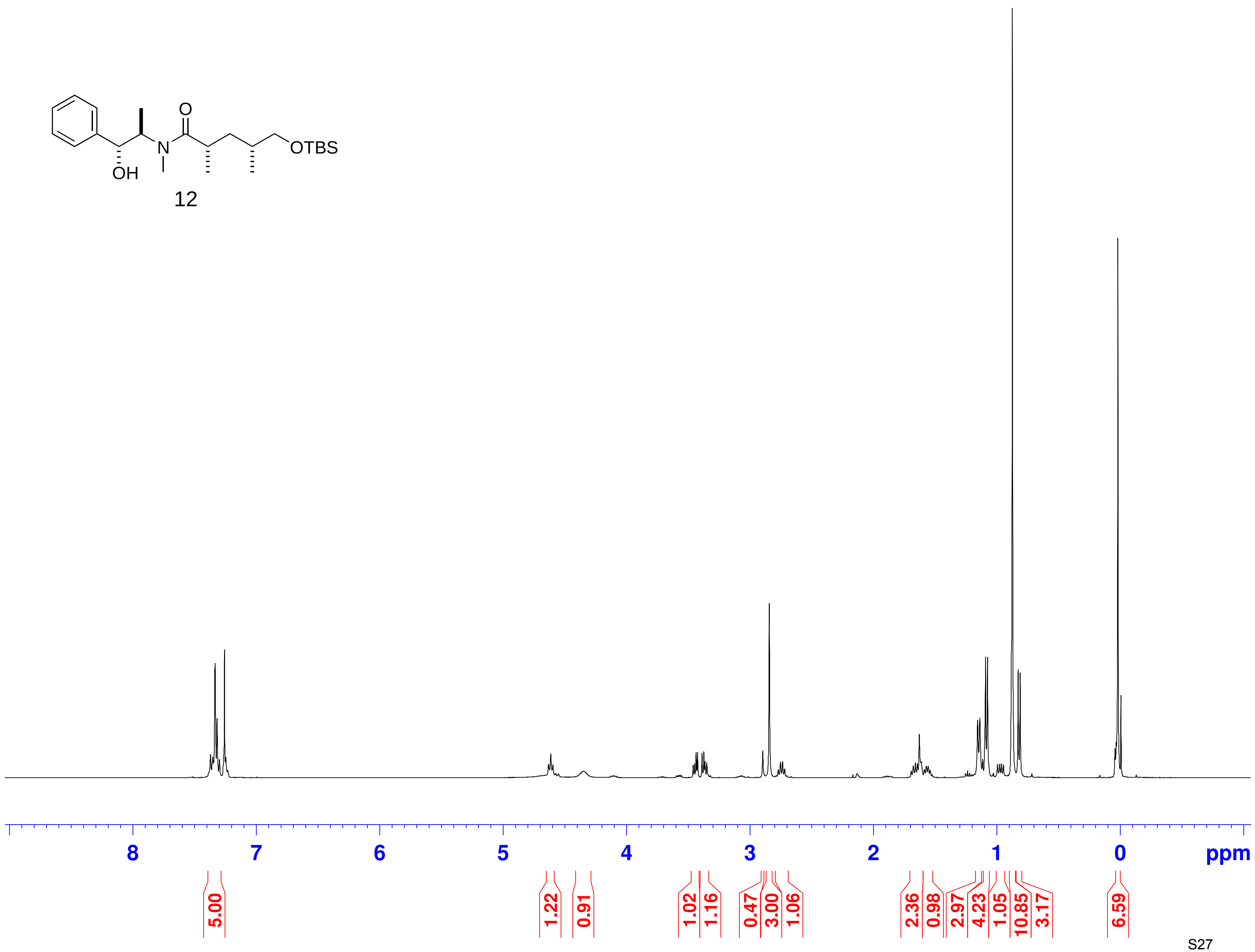

OH
N
O
OTBS
12
8
7
6
5
4
3
2
1
0
ppm
5.00
1.22
0.91
1.02
1.16
0.47
3.00
1.06
2.36
0.98
2.97
4.23
1.05
10.85
3.17
6.59

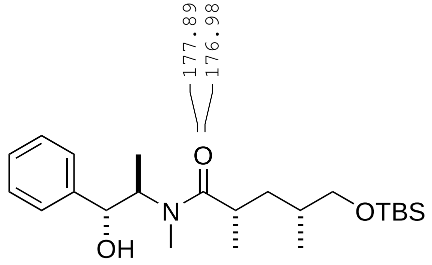
OTBS
OH
N
O

12

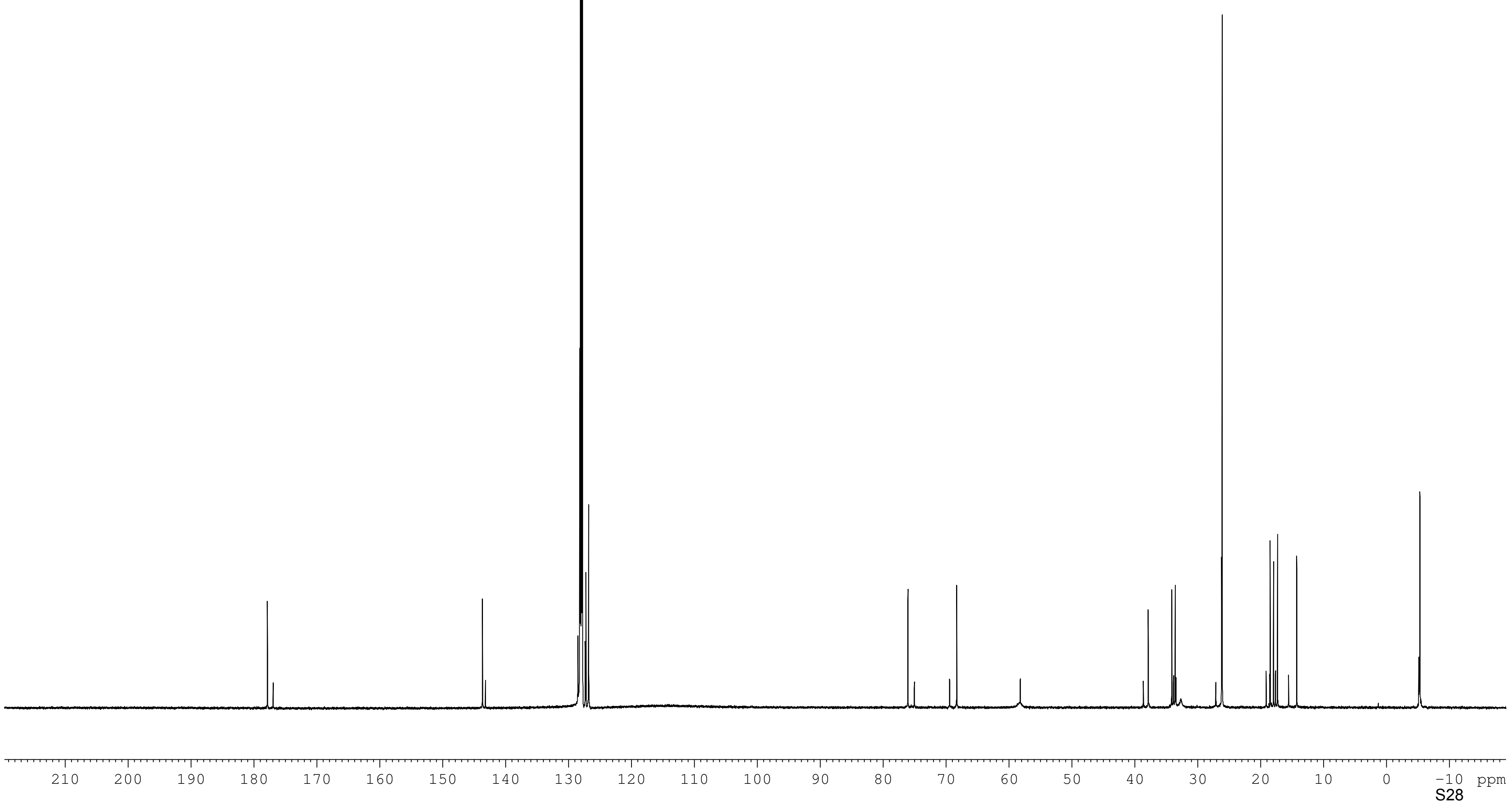
177.89
176.98
143.71
143.23
128.55
128.24
128.16
128.00
127.84
127.38
127.26
126.83
76.09
75.07
69.46
68.33
58.24
38.68
37.89
34.15
33.87
33.61
33.48
27.14
26.25
26.15
19.15
18.58
18.50
17.67
17.35
15.59
14.29
-5.11
-5.17
-5.24
-5.29
210
200
190
180
170
160
150
140
130
120
110
100
90
80
70
60
50
40
30
20
10
0
-10
ppm

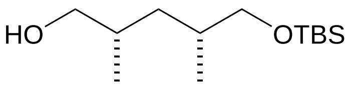
HO
OTBS

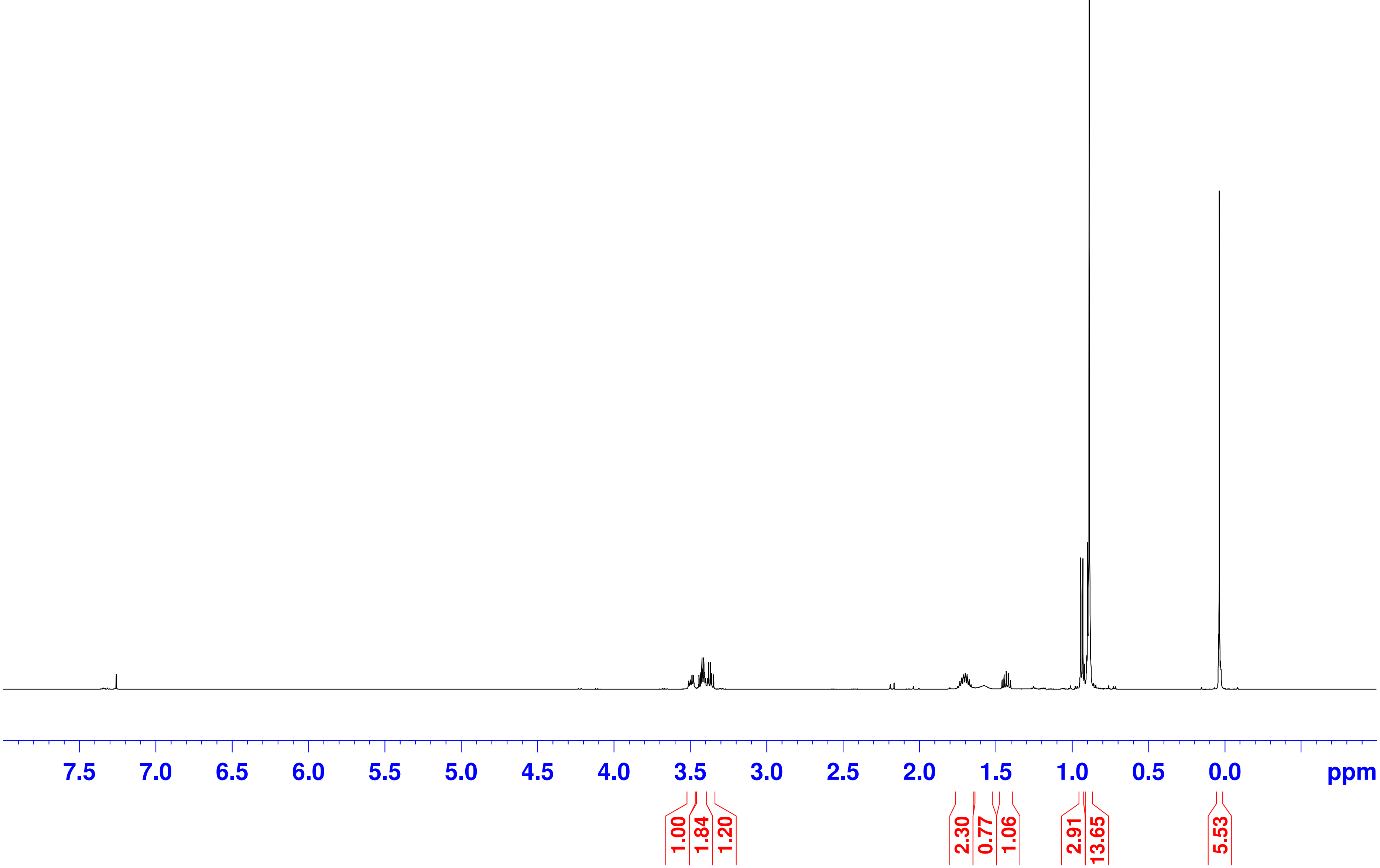
7.5
7.0
6.5
6.0
5.5
5.0
4.5
4.0
3.5
3.0
2.5
2.0
1.5
1.0
0.5
0.0
ppm
1.00
1.84
1.20
2.30
0.77
1.06
2.91
13.65
5.53

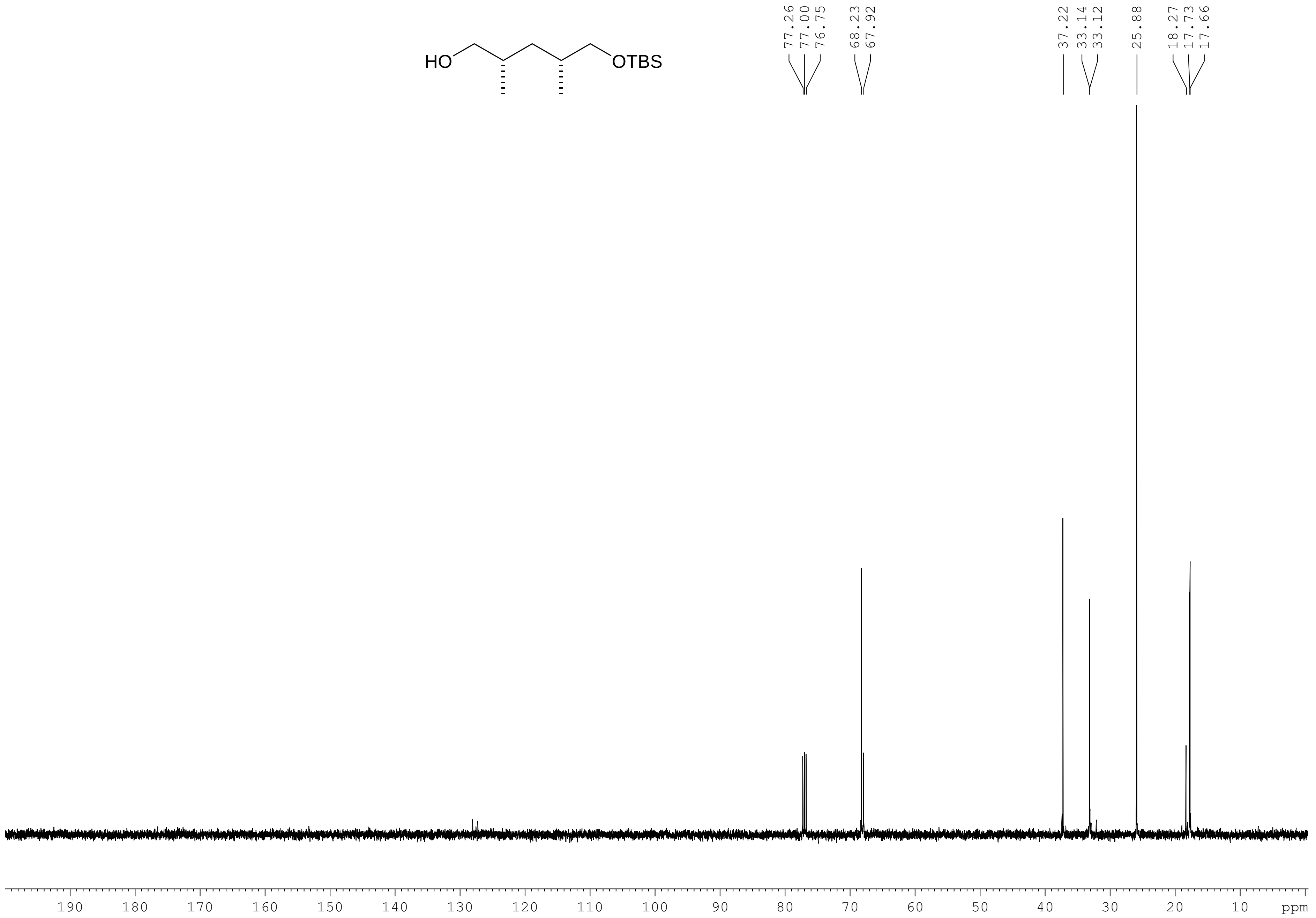

HO
OTBS
77.26
77.00
76.75
68.23
67.92
37.22
33.14
33.12
25.88
18.27
17.73
17.66
190
180
170
160
150
140
130
120
110
100
90
80
70
60
50
40
30
20
10
ppm

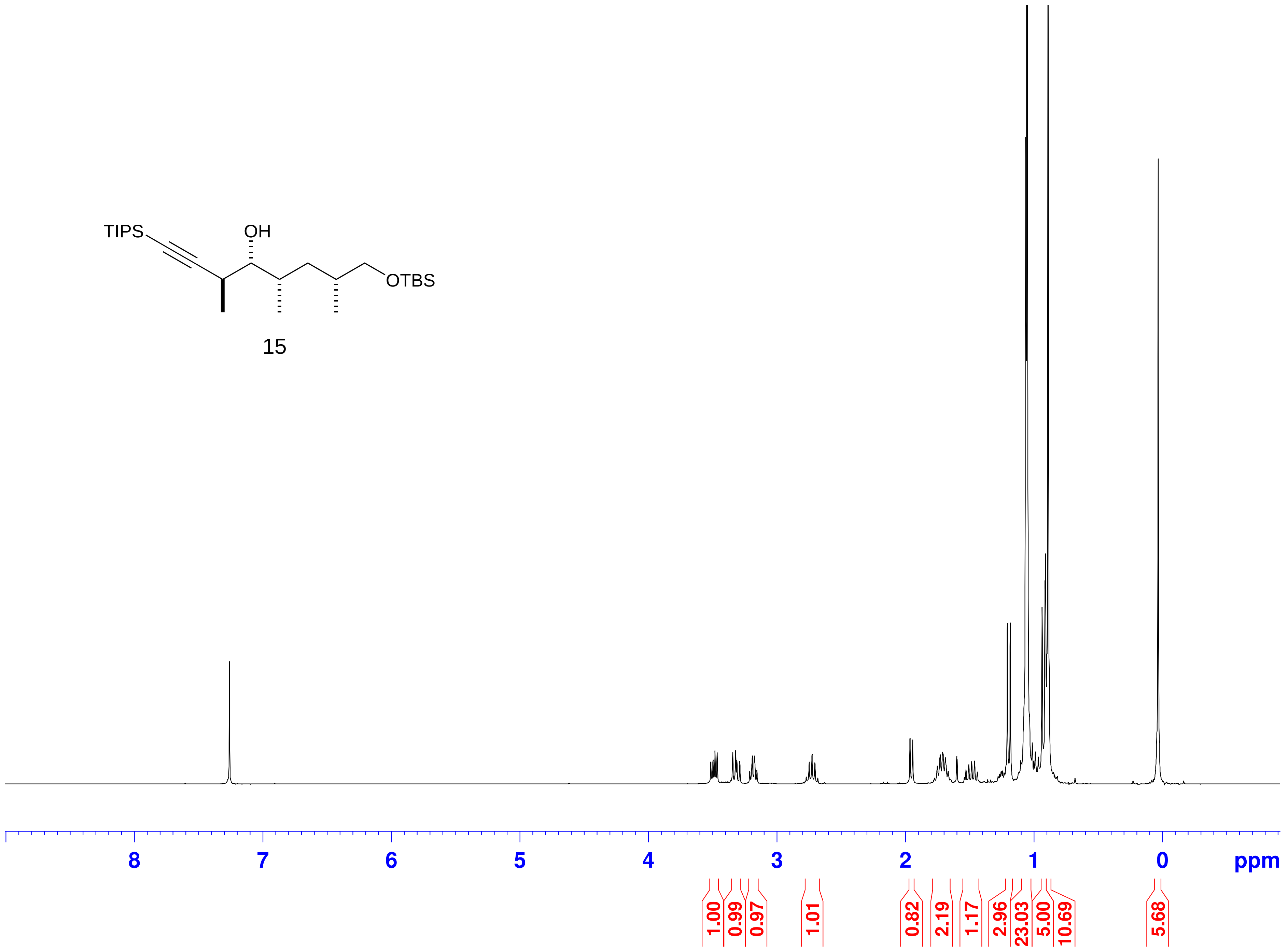
TIPS
OH
OTBS
15
8
7
6
5
4
3
2
1
0
ppm
1.00
0.99
0.97
1.01
0.82
2.19
1.17
2.96
23.03
5.00
10.69
5.68

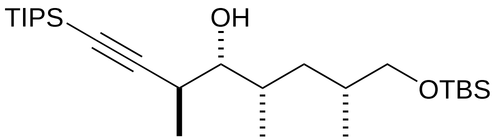
TIPS
OH
OTBS

15

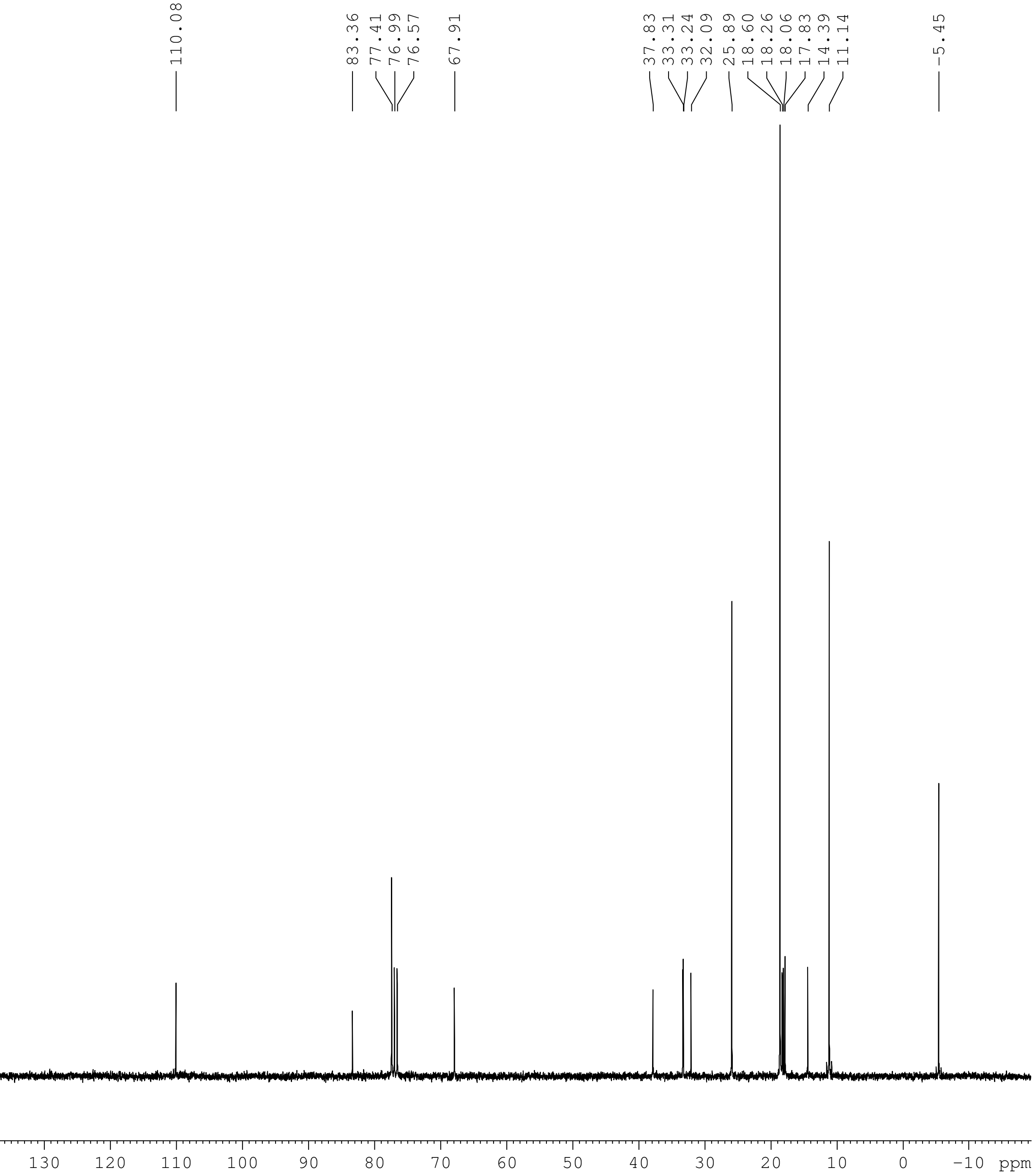
110.08
83.36
77.41
76.99
76.57
67.91
37.83
33.31
33.24
32.09
25.89
18.60
18.26
18.06
17.83
14.39
11.14
-5.45
210 200 190 180 170 160 150 140 130 120 110 100 90 80 70 60 50 40 30 20 10 0 -10 ppm

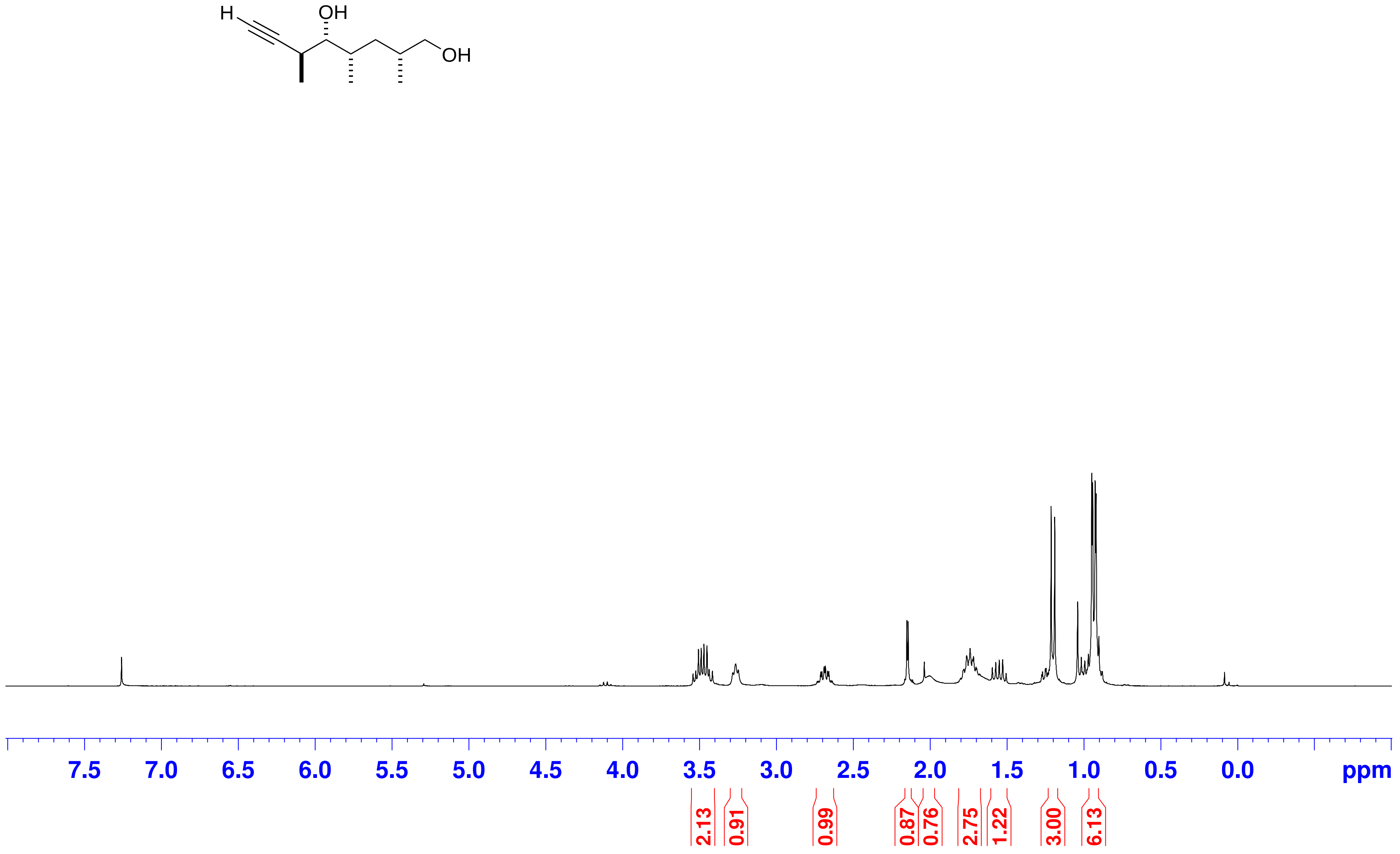

H
OH
OH
7.5
7.0
6.5
6.0
5.5
5.0
4.5
4.0
3.5
3.0
2.5
2.0
1.5
1.0
0.5
0.0
ppm
2.13
0.91
0.99
0.87
0.76
2.75
1.22
3.00
6.13

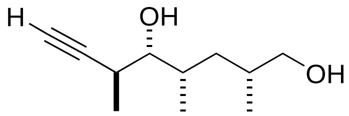

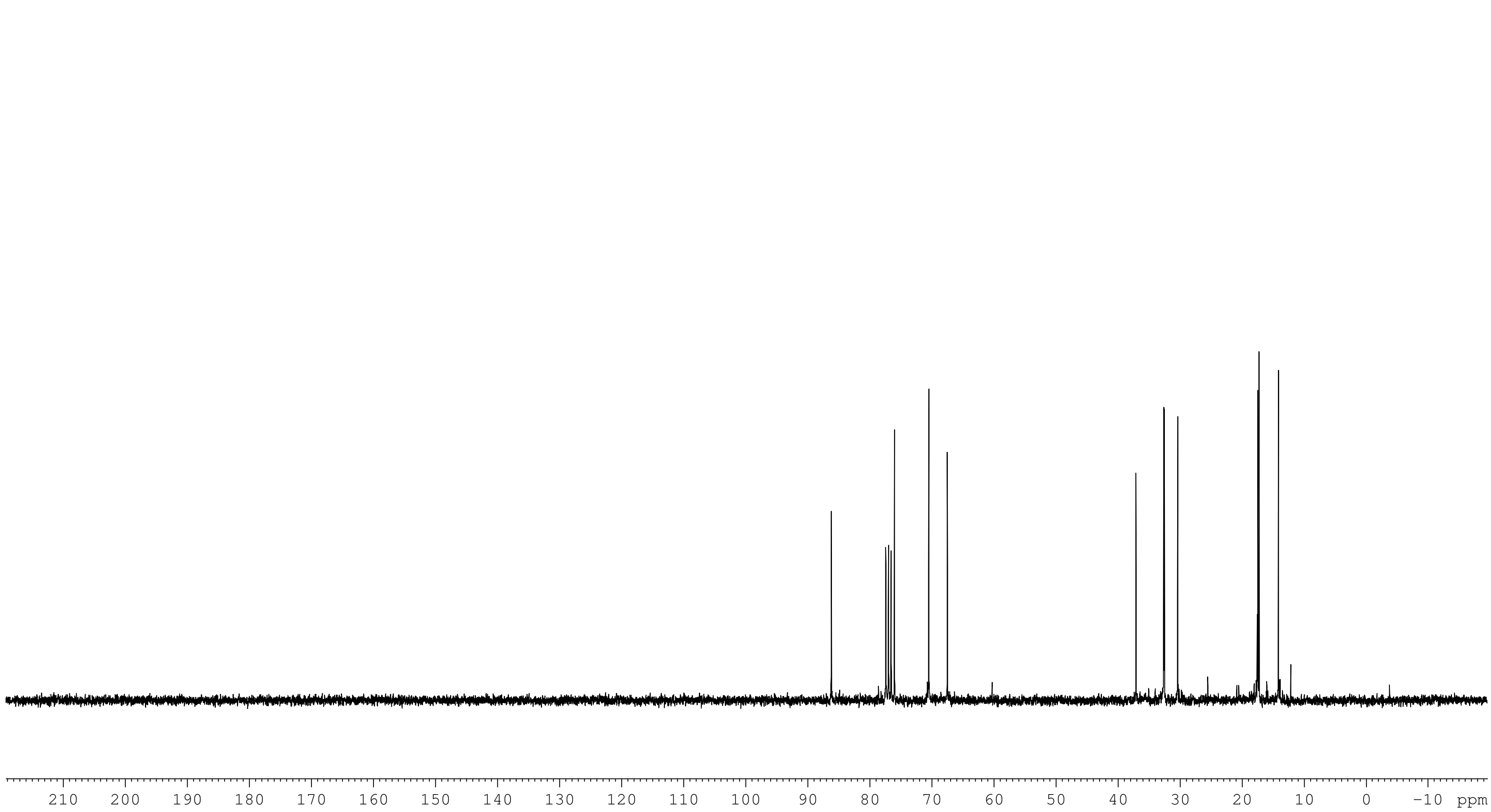

86.22
77.42
76.99
76.57
76.03
70.49
67.50
37.09
32.61
32.51
30.36
17.43
17.24
14.11
210
200
190
180
170
160
150
140
130
120
110
100
90
80
70
60
50
40
30
20
10
0
-10
ppm

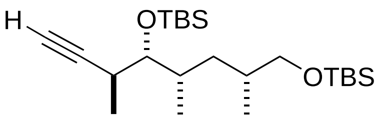

H
OTBS
OTBS

18

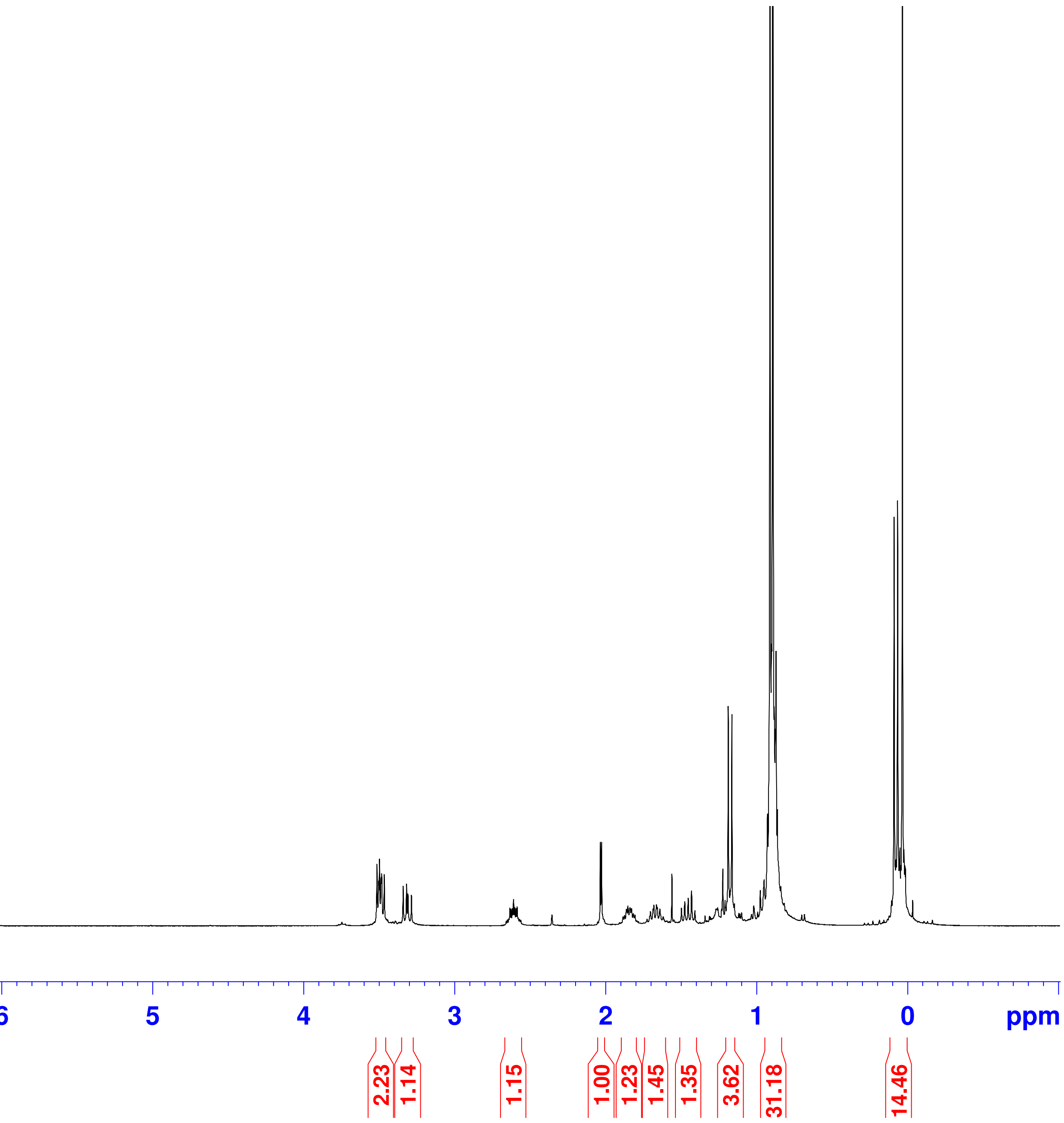

8
7
6
5
4
3
2
1
0
ppm
2.23
1.14
1.15
1.00
1.23
1.45
1.35
3.62
31.18
14.46

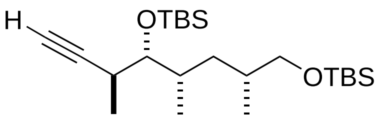
H
OTBS
OTBS

18

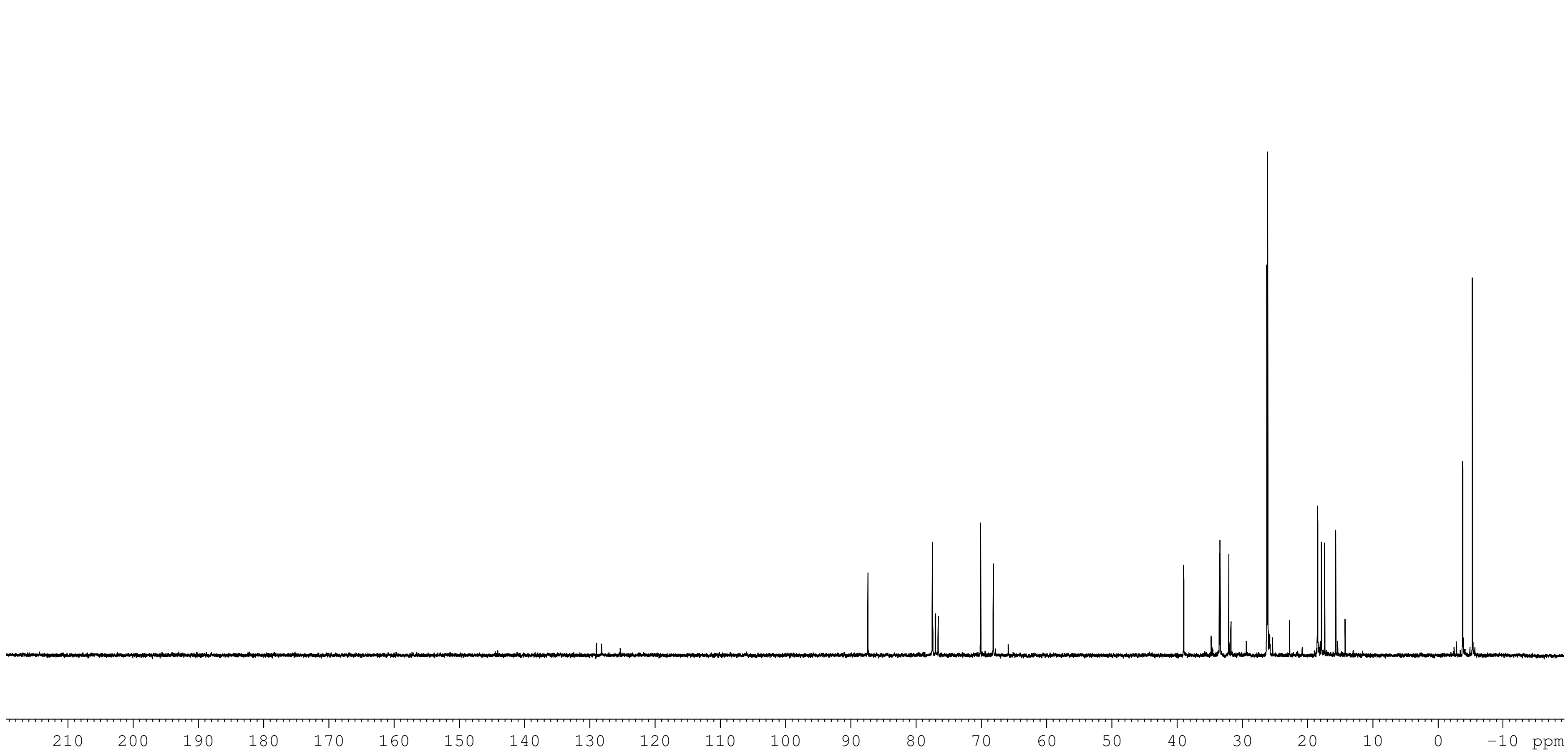
87.36
77.47
77.43
77.00
76.58
70.07
68.12
38.93
33.45
33.34
32.01
26.18
26.04
18.43
18.38
17.79
17.32
15.62
-3.84
-3.87
-5.35
210 200 190 180 170 160 150 140 130 120 110 100 90 80 70 60 50 40 30 20 10 0 -10 ppm

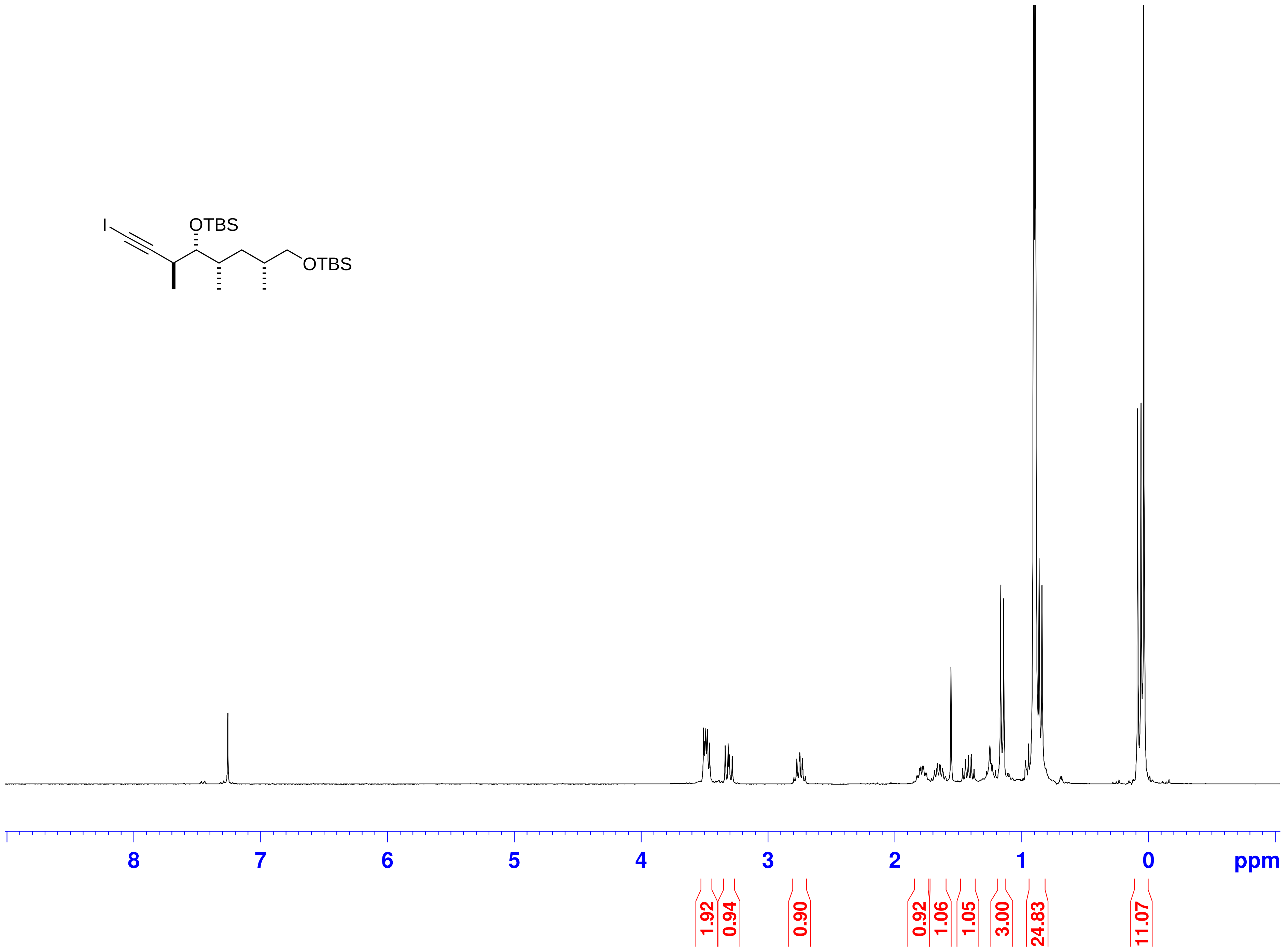

I
OTBS
OTBS
8
7
6
5
4
3
2
1
0
ppm
1.92
0.94
0.90
0.92
1.06
1.05
3.00
24.83
11.07

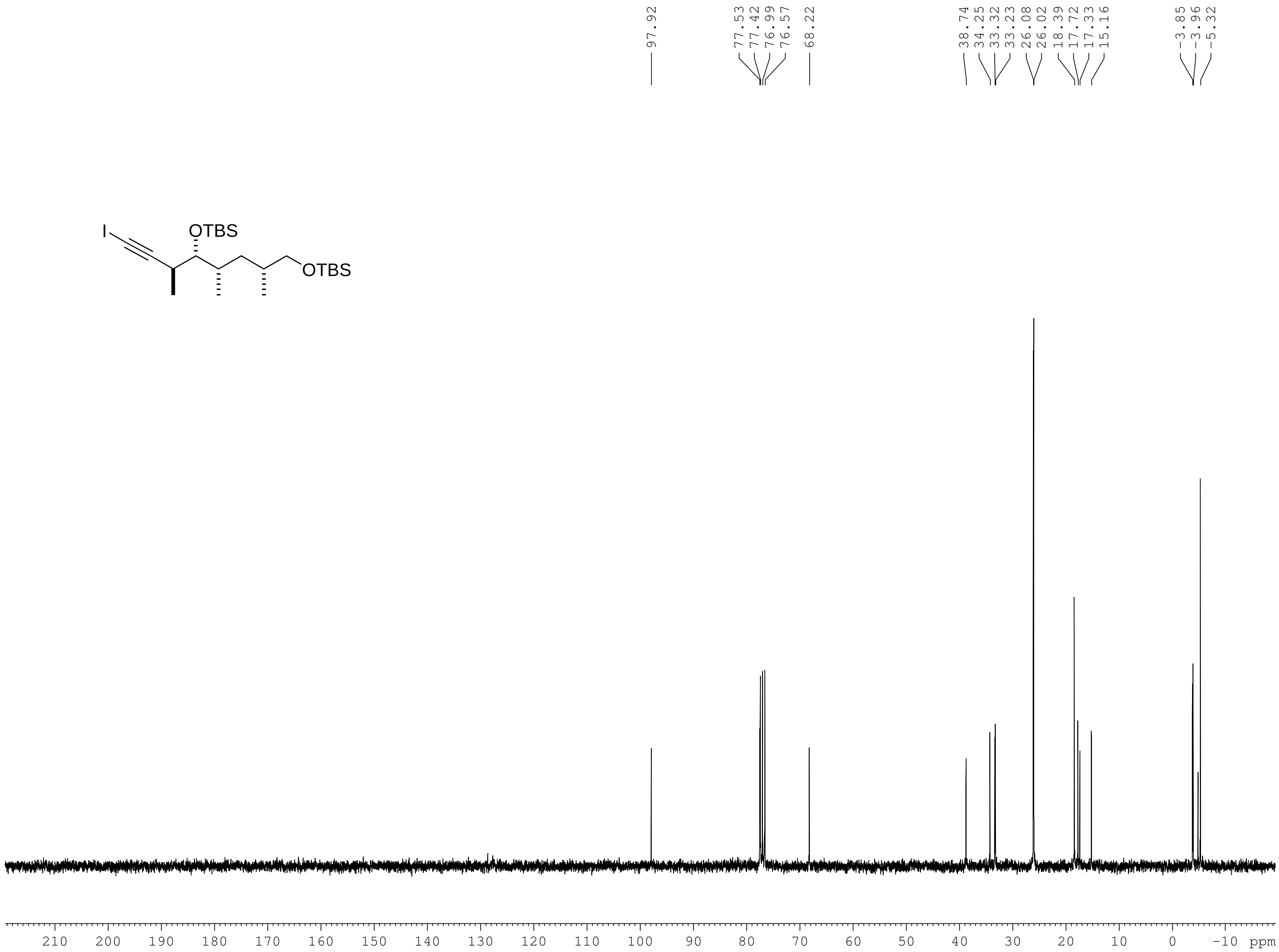
97.92
77.53
77.42
76.99
76.57
68.22
38.74
34.25
33.32
33.23
26.08
26.02
18.39
17.72
17.33
15.16
-3.85
-3.96
-5.32
I
OTBS
OTBS
210 200 190 180 170 160 150 140 130 120 110 100 90 80 70 60 50 40 30 20 10 0 -10 ppm

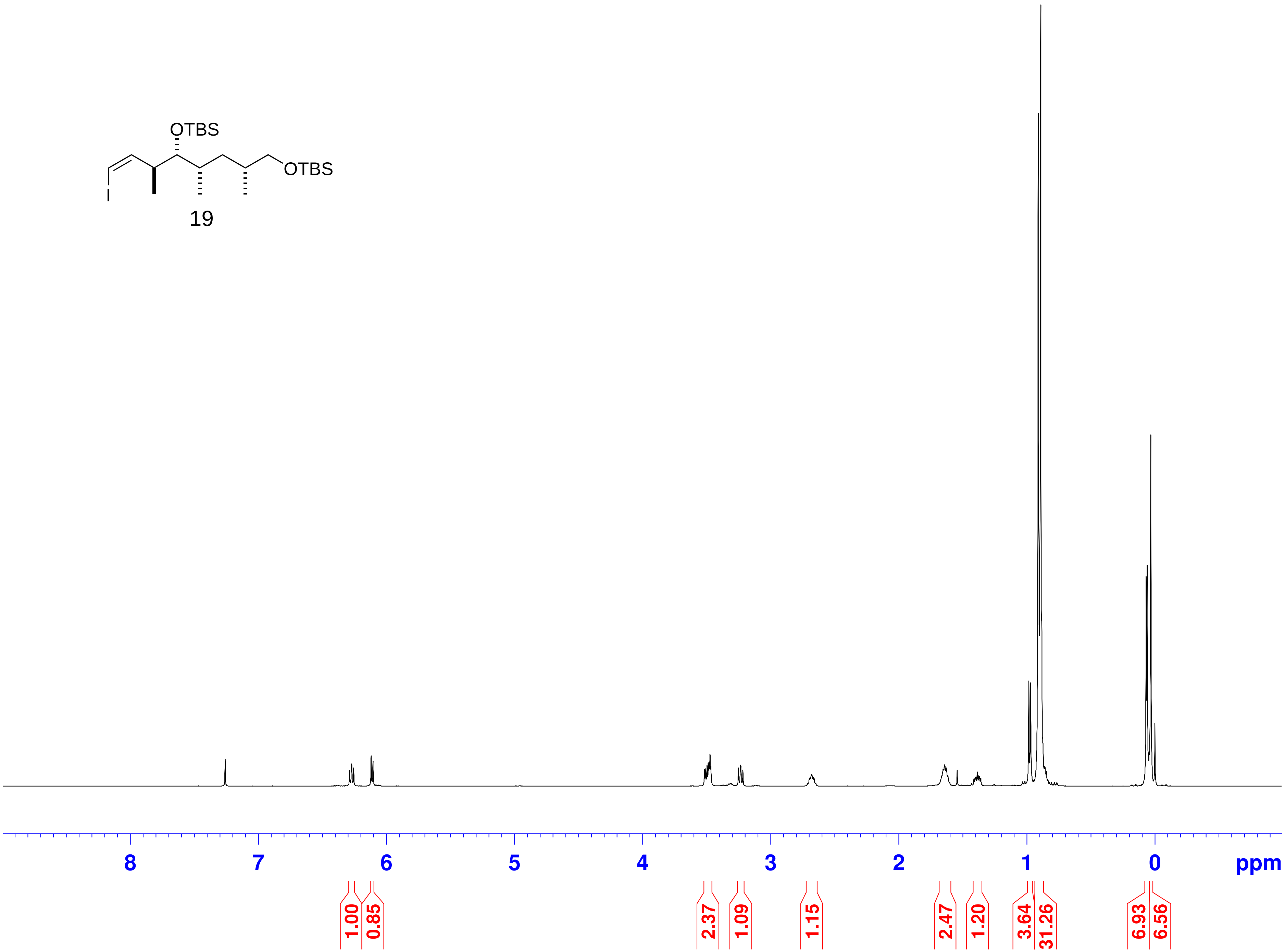
OTBS
OTBS
19
8
7
6
5
4
3
2
1
0
ppm
1.00
0.85
2.37
1.09
1.15
2.47
1.20
3.64
31.26
6.93
6.56

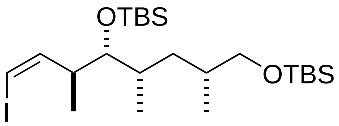
OTBS
OTBS
I

19

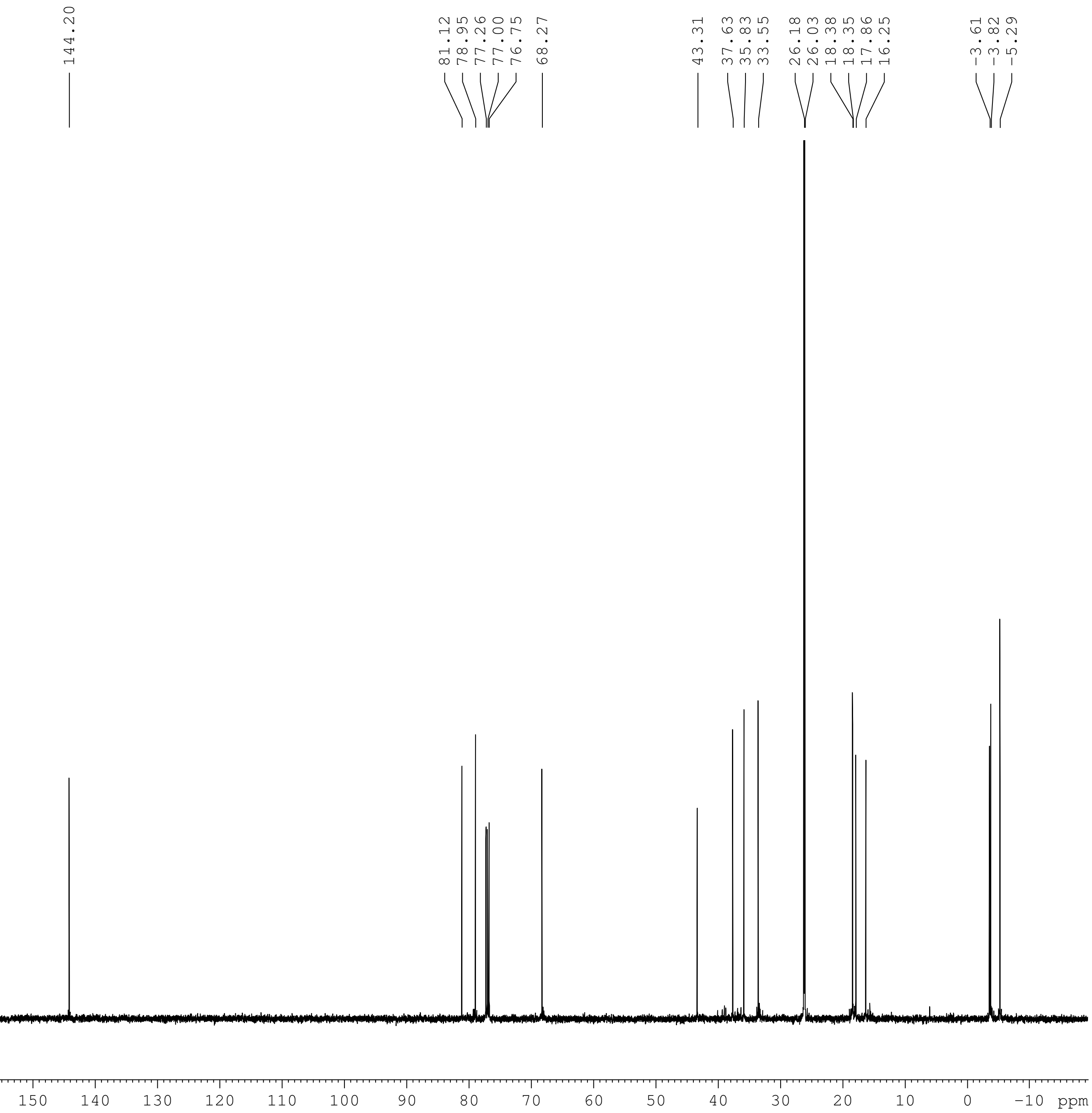
144.20
81.12
78.95
77.26
77.00
76.75
68.27
43.31
37.63
35.83
33.55
26.18
26.03
18.38
18.35
17.86
16.25
-3.61
-3.82
-5.29
210 200 190 180 170 160 150 140 130 120 110 100 90 80 70 60 50 40 30 20 10 0 -10 ppm

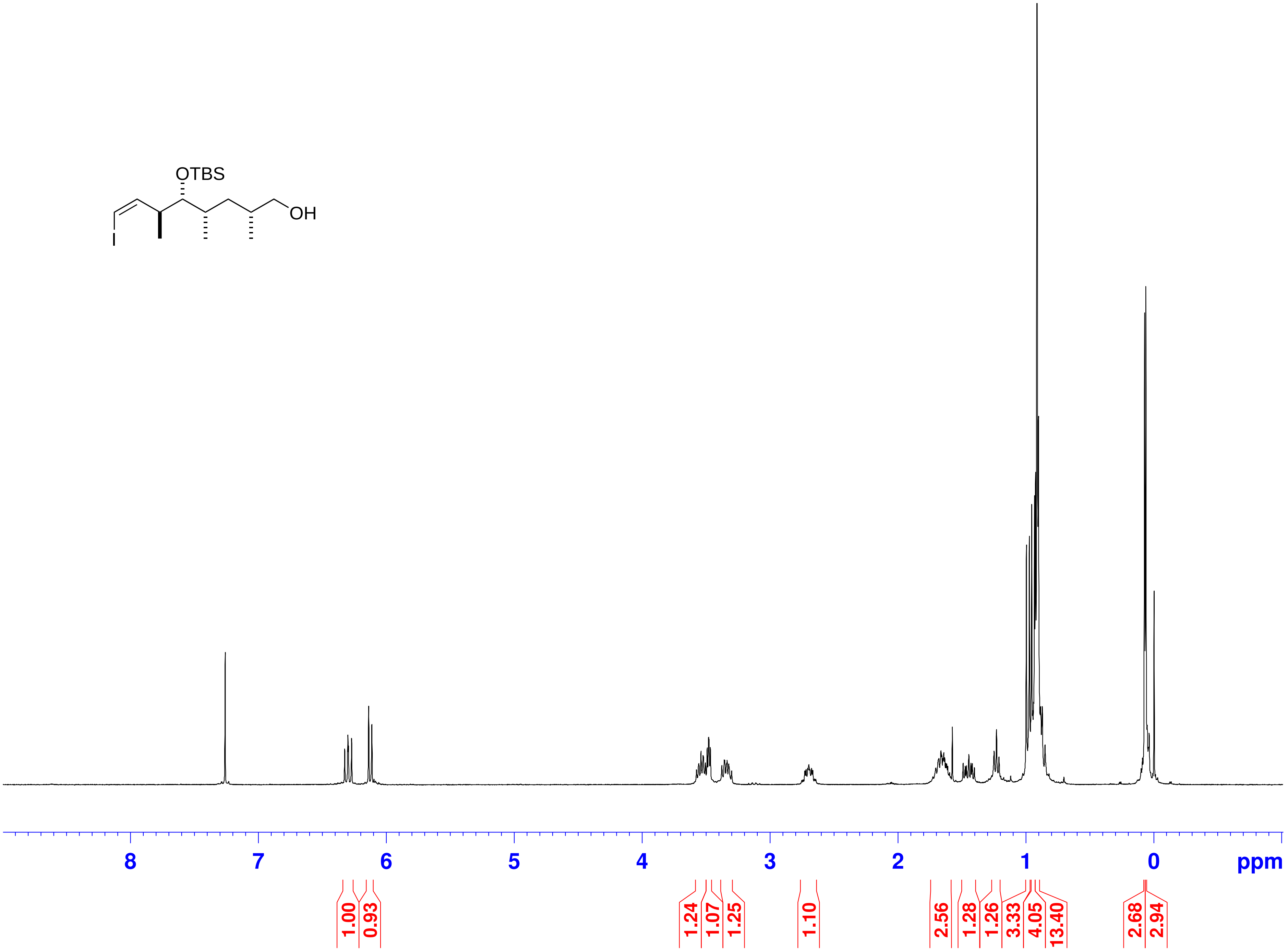

OTBS
OH
I
8
7
6
5
4
3
2
1
0
ppm
1.00
0.93
1.24
1.07
1.25
1.10
2.56
1.28
1.26
3.33
4.05
13.40
2.68
2.94

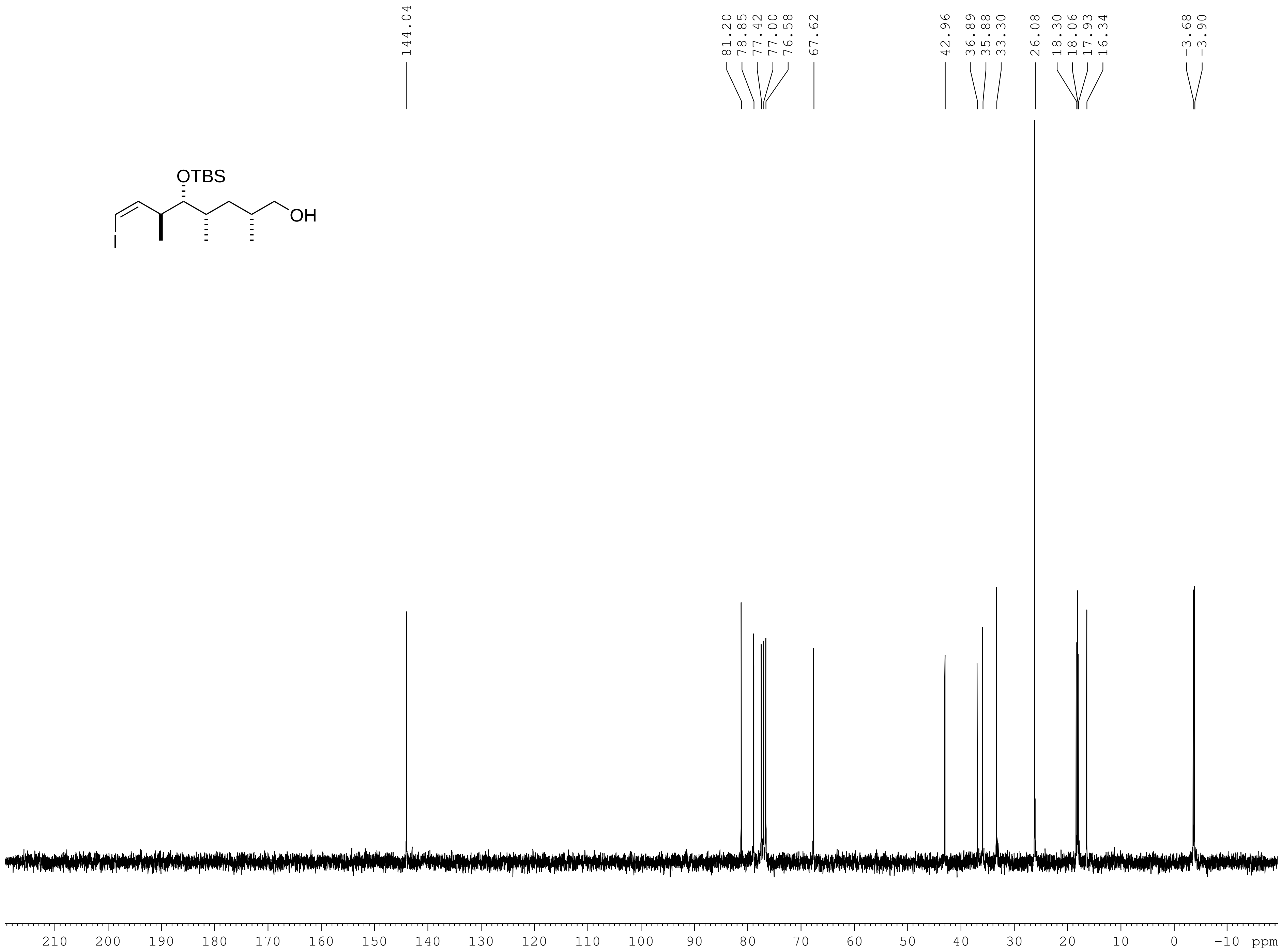
144.04
81.20
78.85
77.42
77.00
76.58
67.62
42.96
36.89
35.88
33.30
26.08
18.30
18.06
17.93
16.34
-3.68
-3.90
OTBS
OH
I
210 200 190 180 170 160 150 140 130 120 110 100 90 80 70 60 50 40 30 20 10 0 -10 ppm

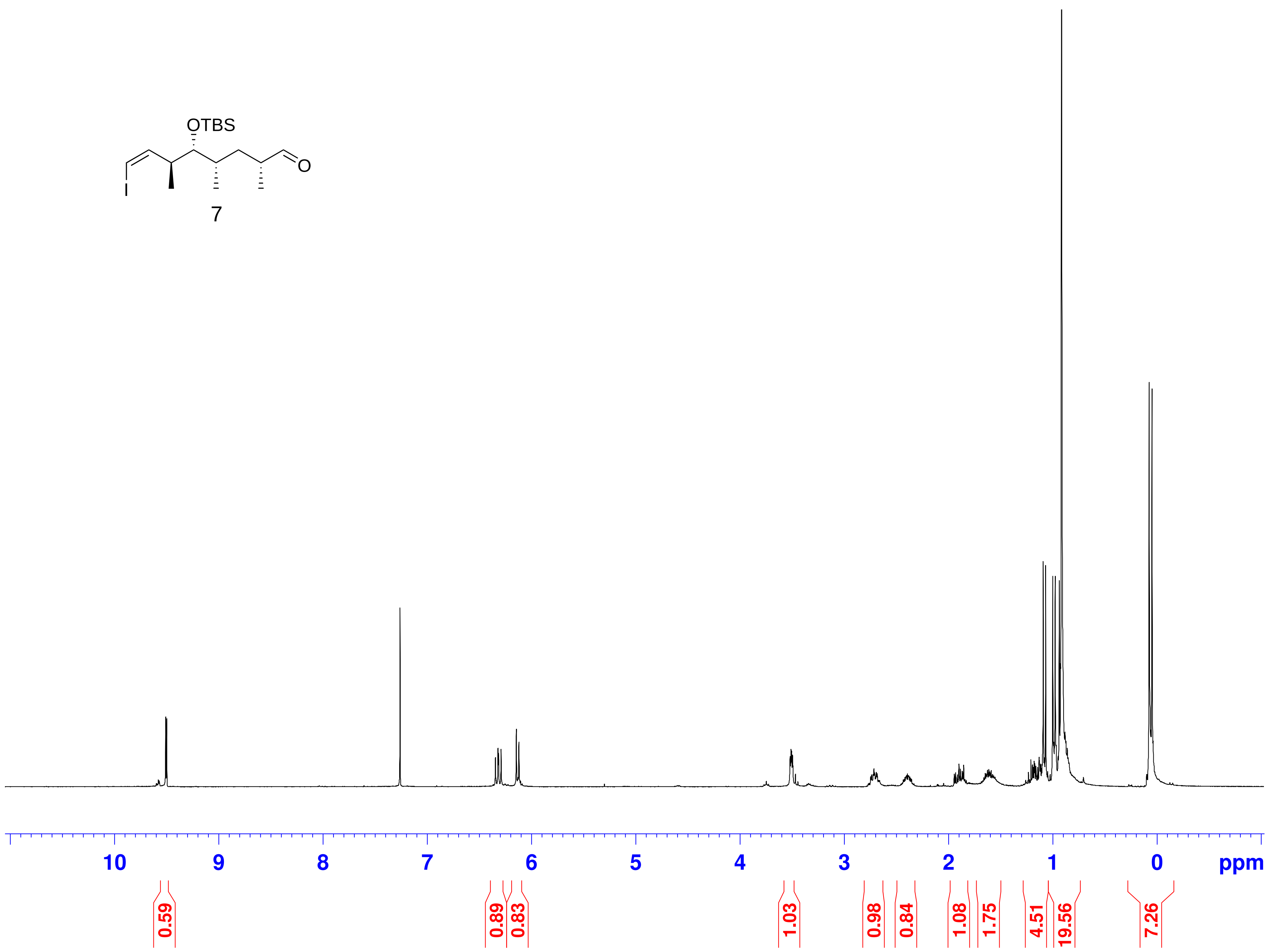
OTBS
O
I
7
10
9
8
7
6
5
4
3
2
1
0
ppm
0.59
0.89
0.83
1.03
0.98
0.84
1.08
1.75
4.51
19.56
7.26

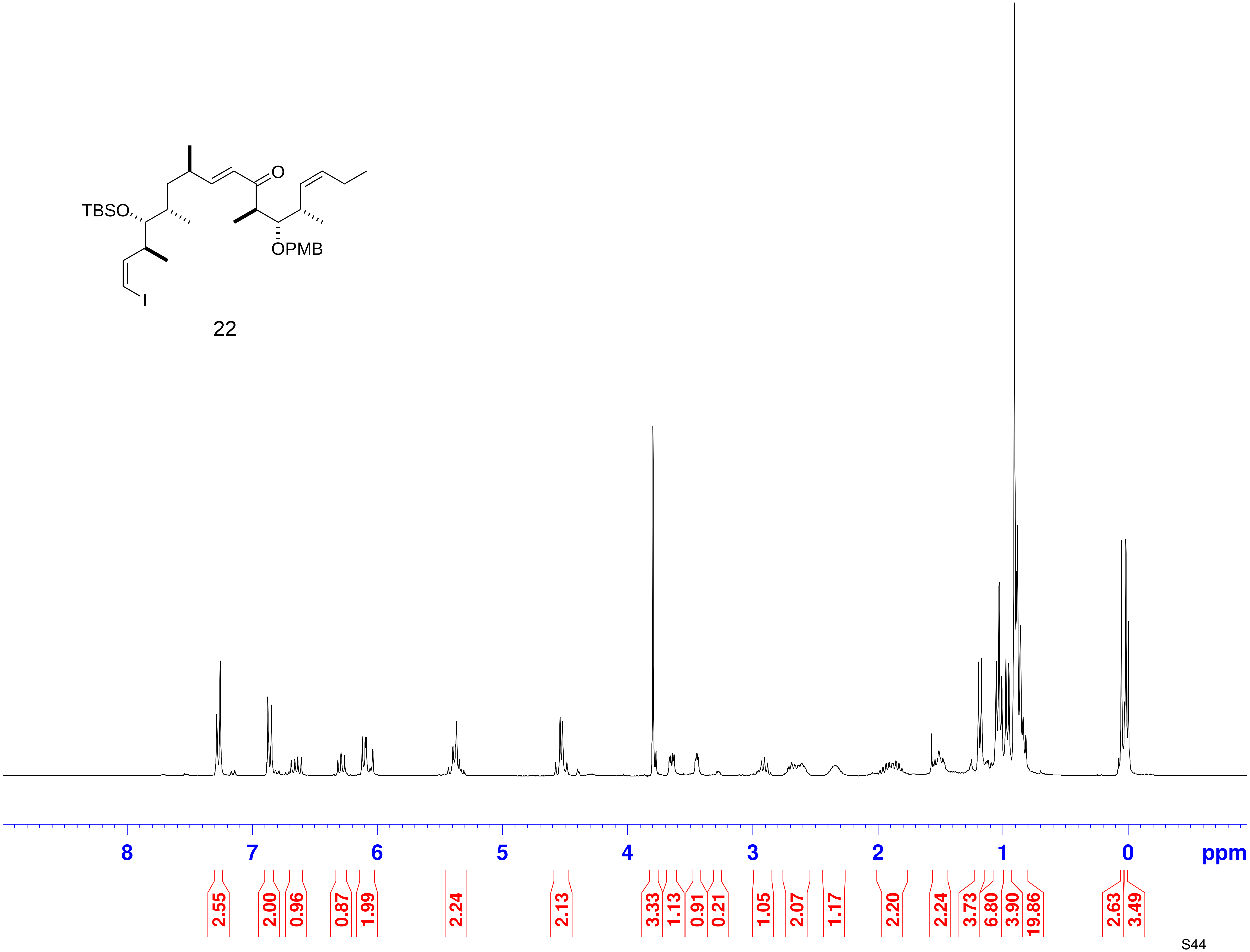
TBSO
O
OPMB
I
8
7
6
5
4
3
2
1
0
ppm
2.55
2.00
0.96
0.87
1.99
2.24
2.13
3.33
1.13
0.91
0.21
1.05
2.07
1.17
2.20
2.24
3.73
6.80
3.90
19.86
2.63
3.49

22

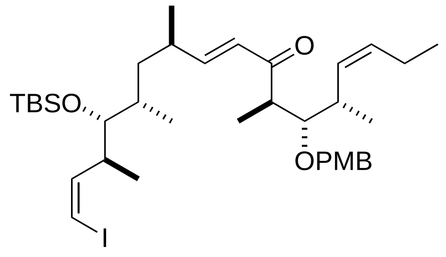
TBSO
OPMB
O
I

22

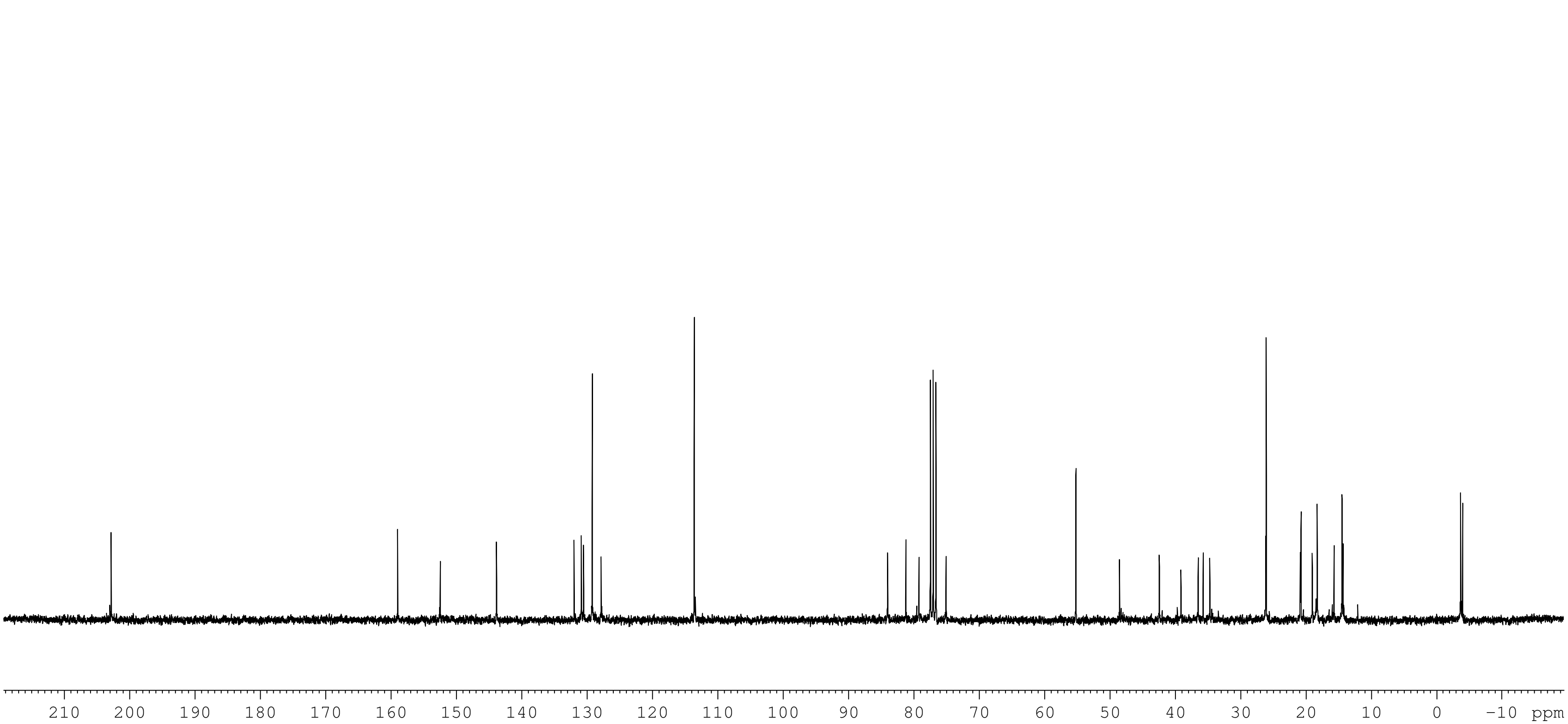
202.86
159.00
152.46
143.85
131.98
130.90
130.52
129.19
127.84
113.59
83.99
81.18
79.18
77.43
77.01
76.58
75.04
55.16
48.49
42.39
39.08
36.44
35.67
34.65
26.09
26.03
20.81
20.70
18.97
18.25
18.18
15.64
14.42
14.24
-3.71
-4.04
210 200 190 180 170 160 150 140 130 120 110 100 90 80 70 60 50 40 30 20 10 0 -10 ppm

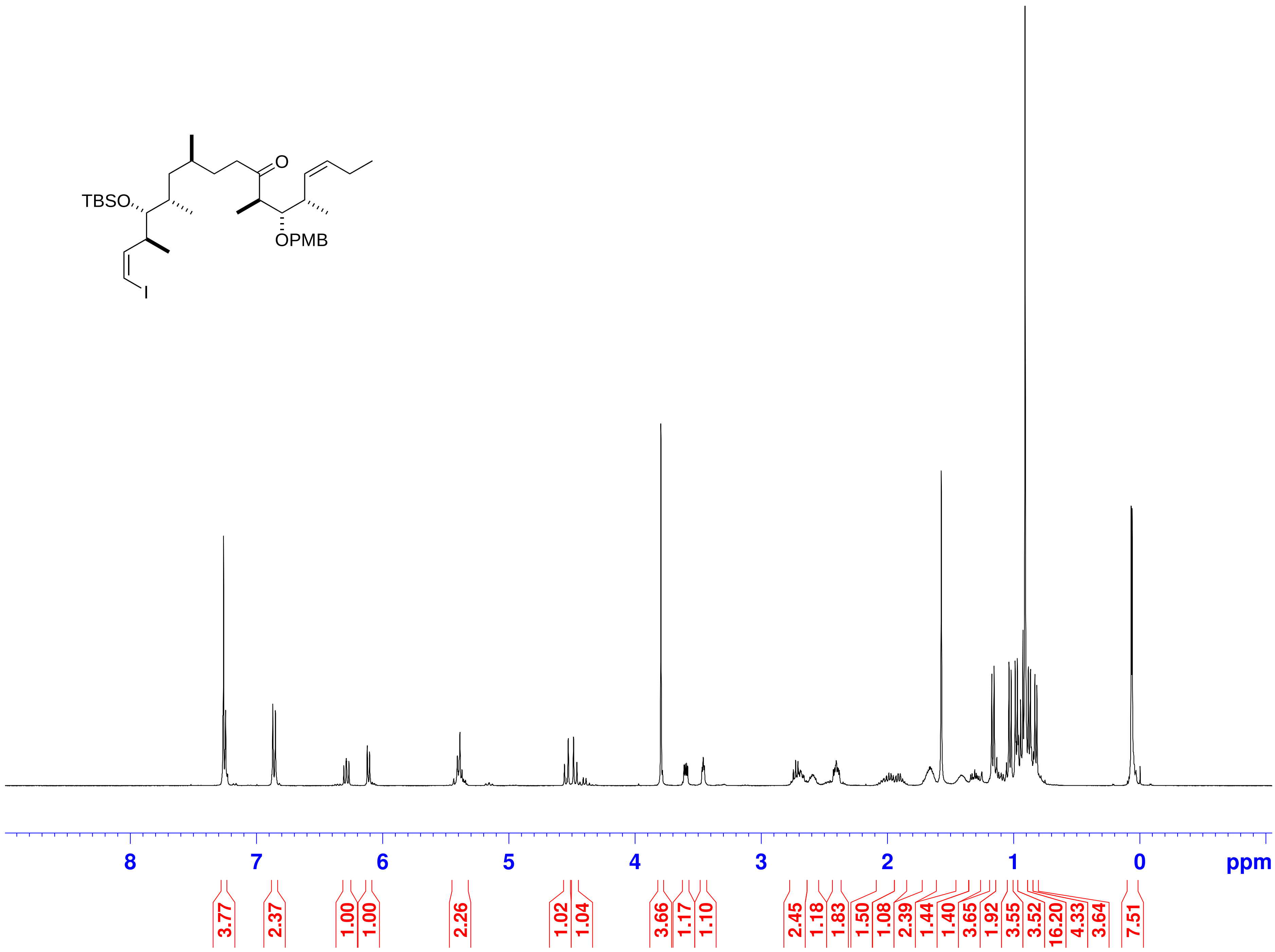
TBSO
O
OPMB
I
8
7
6
5
4
3
2
1
0
ppm
3.77
2.37
1.00
1.00
2.26
1.02
1.04
3.66
1.17
1.10
2.45
1.18
1.83
1.50
1.08
2.39
1.44
1.40
3.65
1.92
3.55
3.52
16.20
4.33
3.64
7.51

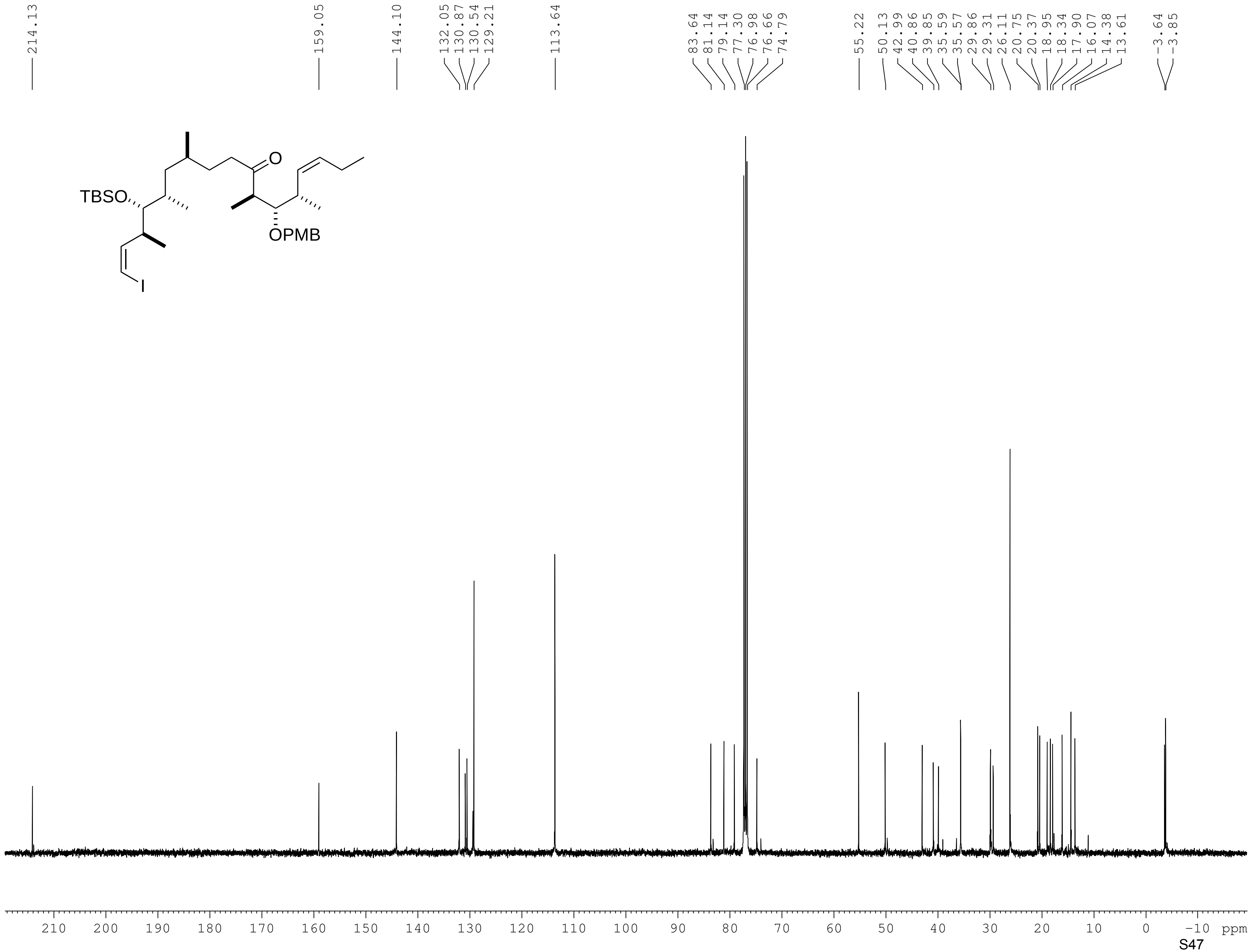
214.13
159.05
144.10
132.05
130.87
130.54
129.21
113.64
83.64
81.14
79.14
77.30
76.98
76.66
74.79
55.22
50.13
42.99
40.86
39.85
35.59
35.57
29.86
29.31
26.11
20.75
20.37
18.95
18.34
17.90
16.07
14.38
13.61
-3.64
-3.85
TBSO
O
OPMB
I
210 200 190 180 170 160 150 140 130 120 110 100 90 80 70 60 50 40 30 20 10 0 -10 ppm

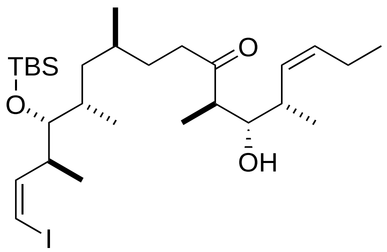

23

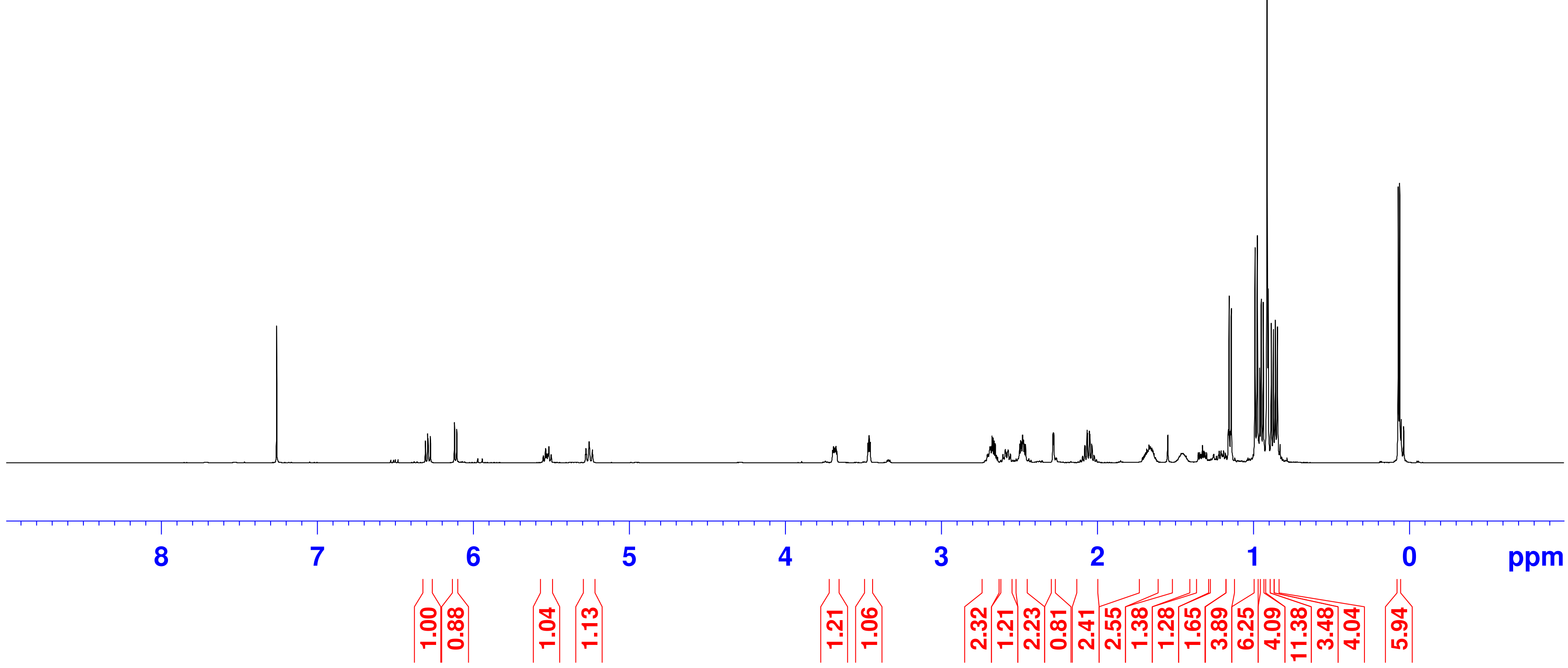

8
7
6
5
4
3
2
1
0
ppm
1.00
0.88
1.04
1.13
1.21
1.06
2.32
1.21
2.23
0.81
2.41
2.55
1.38
1.28
1.65
3.89
6.25
4.09
11.38
3.48
4.04
5.94

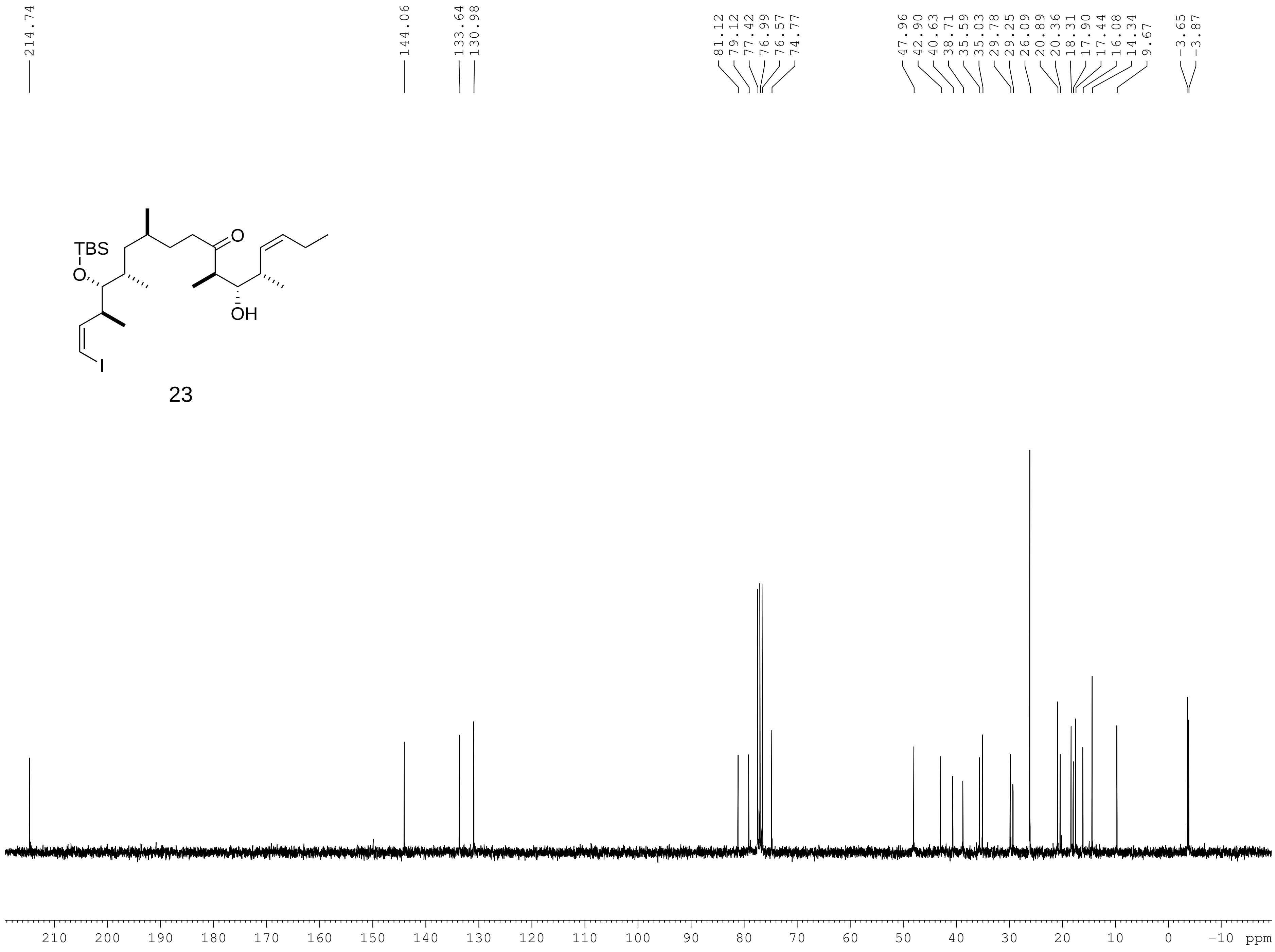
214.74
144.06
133.64
130.98
81.12
79.12
77.42
76.99
76.57
74.77
47.96
42.90
40.63
38.71
35.59
35.03
29.78
29.25
26.09
20.89
20.36
18.31
17.90
17.44
16.08
14.34
9.67
-3.65
-3.87
TBS
O
OH
O
I
23
210 200 190 180 170 160 150 140 130 120 110 100 90 80 70 60 50 40 30 20 10 0 -10 ppm

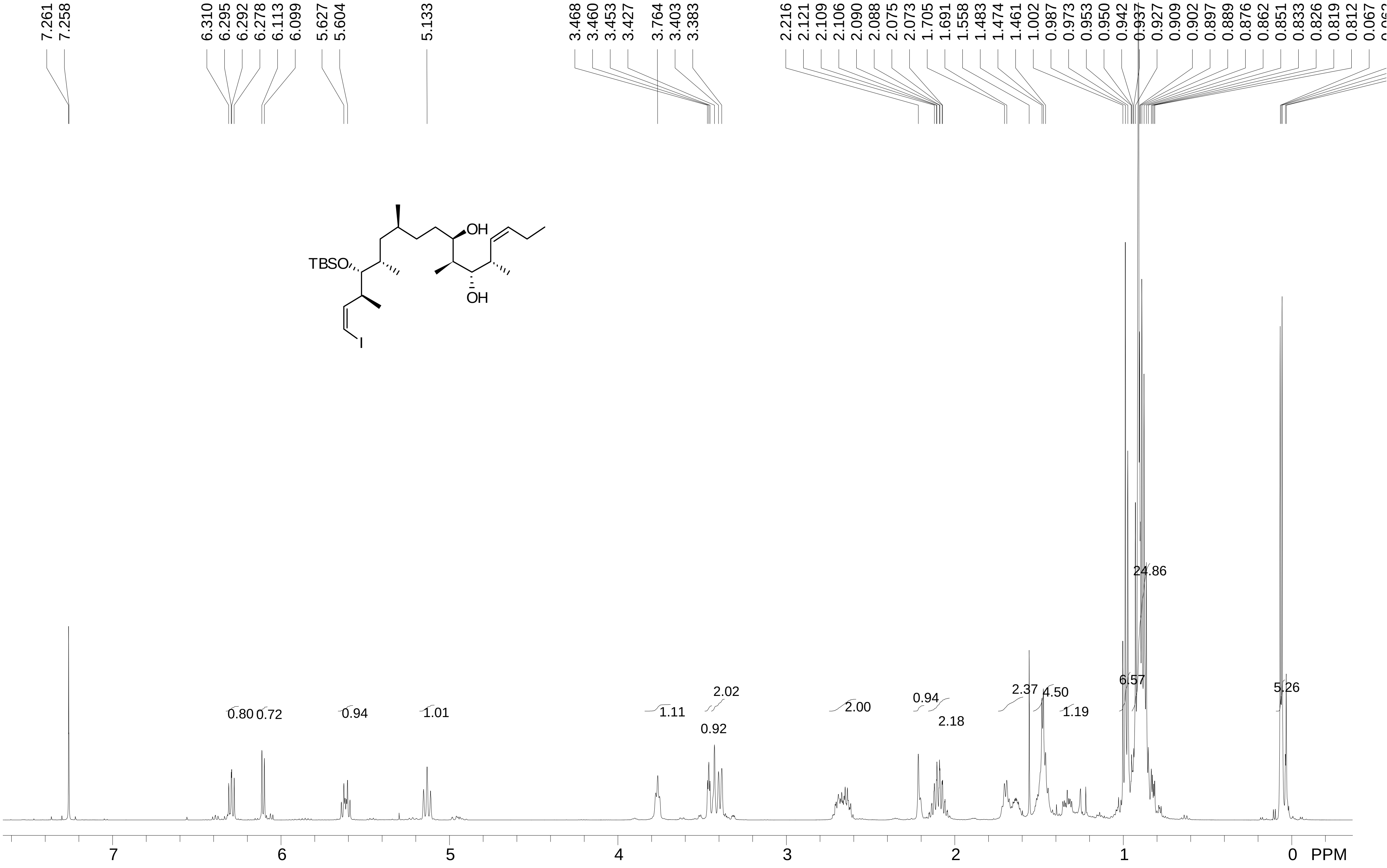
7.261
7.258
6.310
6.295
6.292
6.278
6.113
6.099
5.627
5.604
5.133
3.468
3.460
3.453
3.427
3.764
3.403
3.383
2.216
2.121
2.109
2.106
2.090
2.088
2.075
2.073
1.705
1.691
1.558
1.483
1.474
1.461
1.002
0.987
0.973
0.953
0.950
0.942
0.937
0.927
0.909
0.902
0.897
0.889
0.876
0.862
0.851
0.833
0.826
0.819
0.812
0.067
TBSO
OH
OH
I
0.80
0.72
0.94
1.01
1.11
2.02
0.92
2.00
0.94
2.18
2.37
4.50
1.19
6.57
24.86
5.26
7
6
5
4
3
2
1
0
PPM

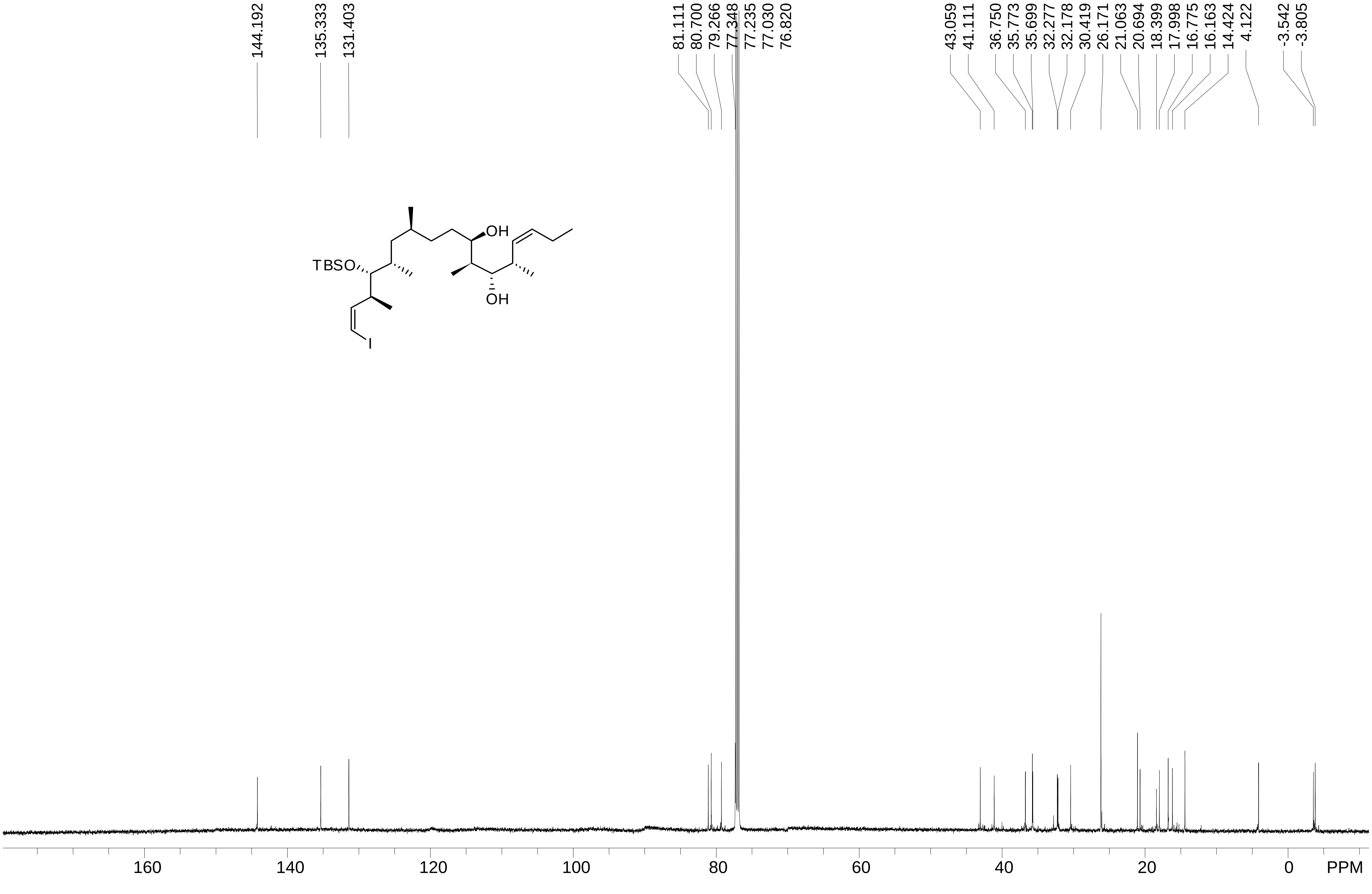
144.192
135.333
131.403
81.111
80.700
79.266
77.348
77.235
77.030
76.820
43.059
41.111
36.750
35.773
35.699
32.277
32.178
30.419
26.171
21.063
20.694
18.399
17.998
16.775
16.163
14.424
4.122
-3.542
-3.805
TBSO
OH
OH
I
160
140
120
100
80
60
40
20
0
PPM

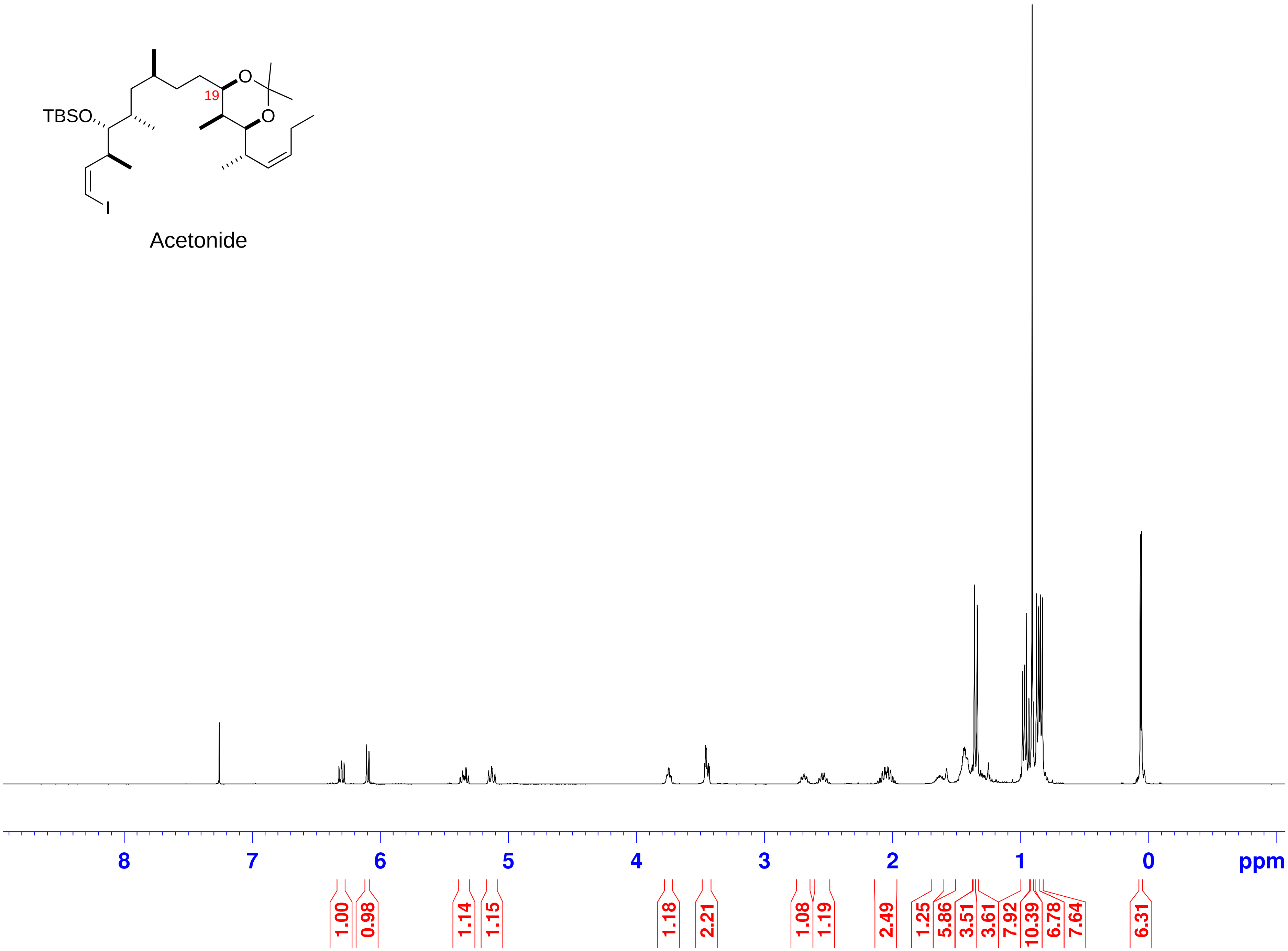

TBSO
19
O
O
I
Acetonide
8
7
6
5
4
3
2
1
0
ppm
1.00
0.98
1.14
1.15
1.18
2.21
1.08
1.19
2.49
1.25
5.86
3.51
3.61
7.92
10.39
6.78
7.64
6.31

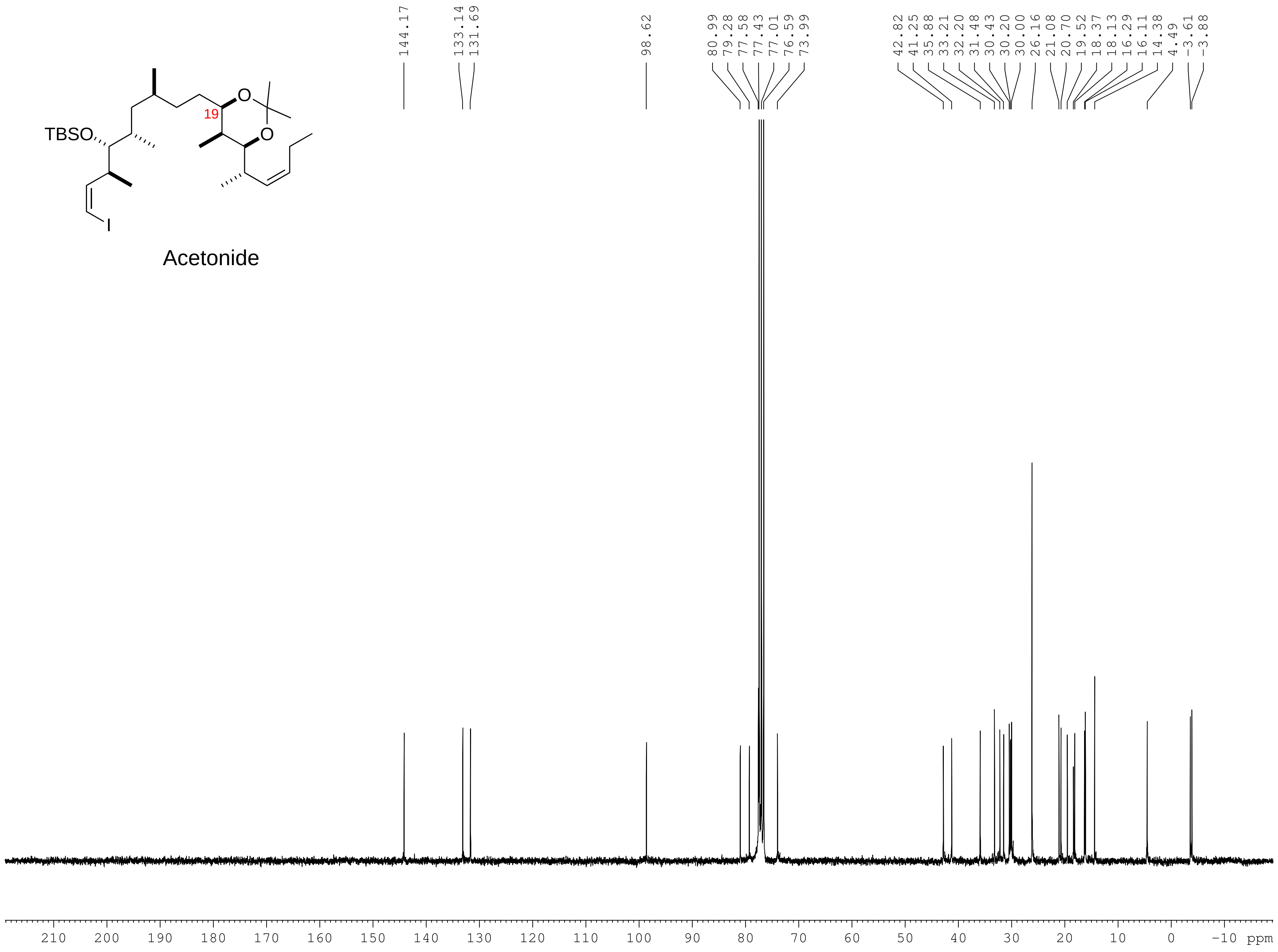
144.17
133.14
131.69
98.62
80.99
79.28
77.58
77.43
77.01
76.59
73.99
42.82
41.25
35.88
33.21
32.20
31.48
30.43
30.20
30.00
26.16
21.08
20.70
19.52
18.37
18.13
16.29
16.11
14.38
4.49
-3.61
-3.88
TBSO
O
O
19
I
Acetonide
210 200 190 180 170 160 150 140 130 120 110 100 90 80 70 60 50 40 30 20 10 0 -10 ppm

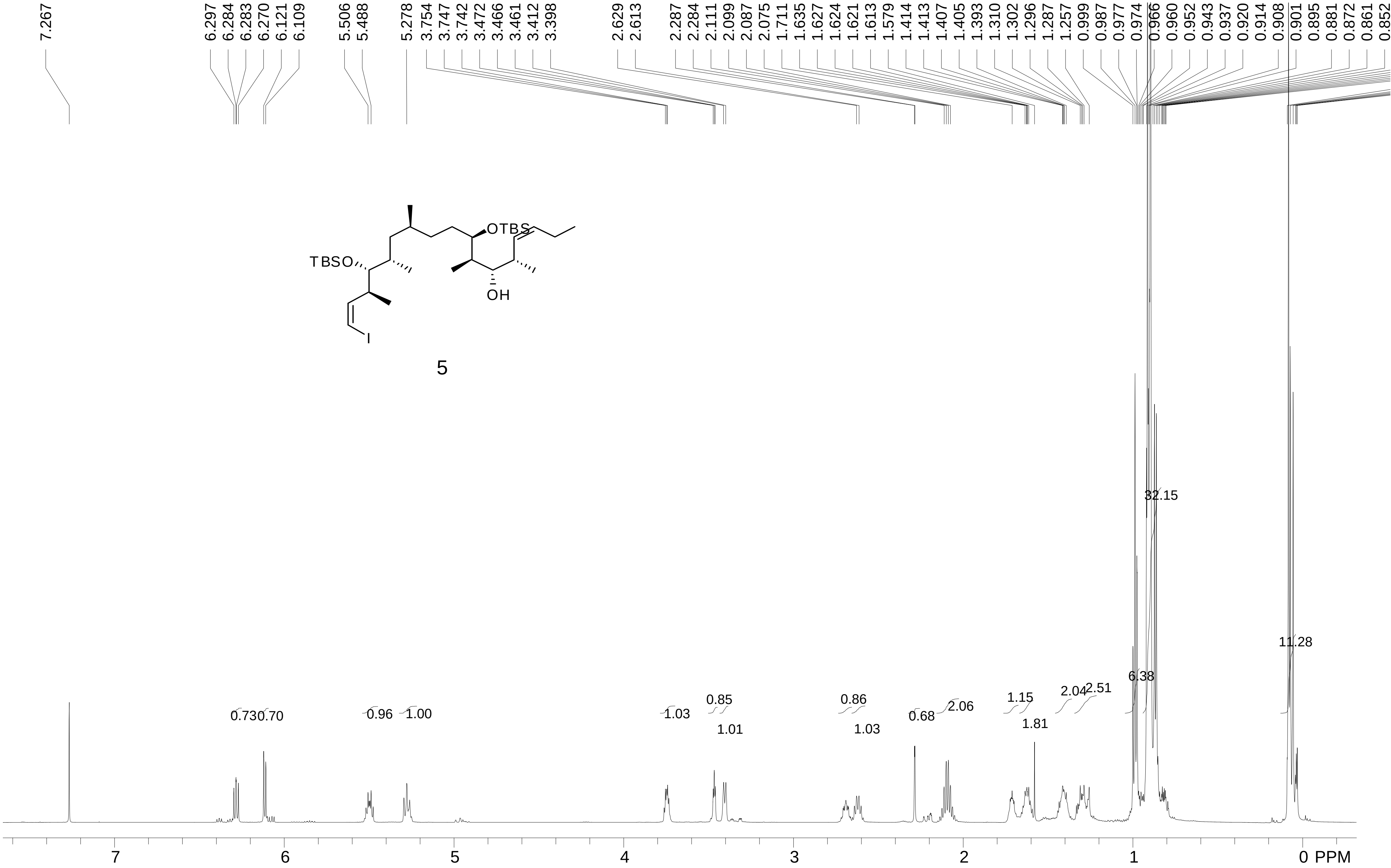
7.267
6.297 6.284 6.283 6.270 6.121 6.109
5.506 5.488
5.278 3.754 3.747 3.742 3.472 3.466 3.461 3.412 3.398
2.629 2.613
2.287 2.284 2.111 2.099 2.087 2.075 1.711 1.635 1.627 1.624 1.621 1.613 1.579 1.414 1.413 1.407 1.405 1.393 1.310 1.302 1.296 1.287 1.257 0.999 0.987 0.977 0.974 0.966 0.960 0.952 0.943 0.937 0.920 0.914 0.908 0.901 0.895 0.881 0.872 0.861 0.852
TBSO
OTBS
OH
I
5
0.73 0.70
0.96
1.00
1.03
0.85
1.01
0.86
1.03
0.68
2.06
1.15
1.81
2.04
2.51
6.38
32.15
11.28
7
6
5
4
3
2
1
0 PPM

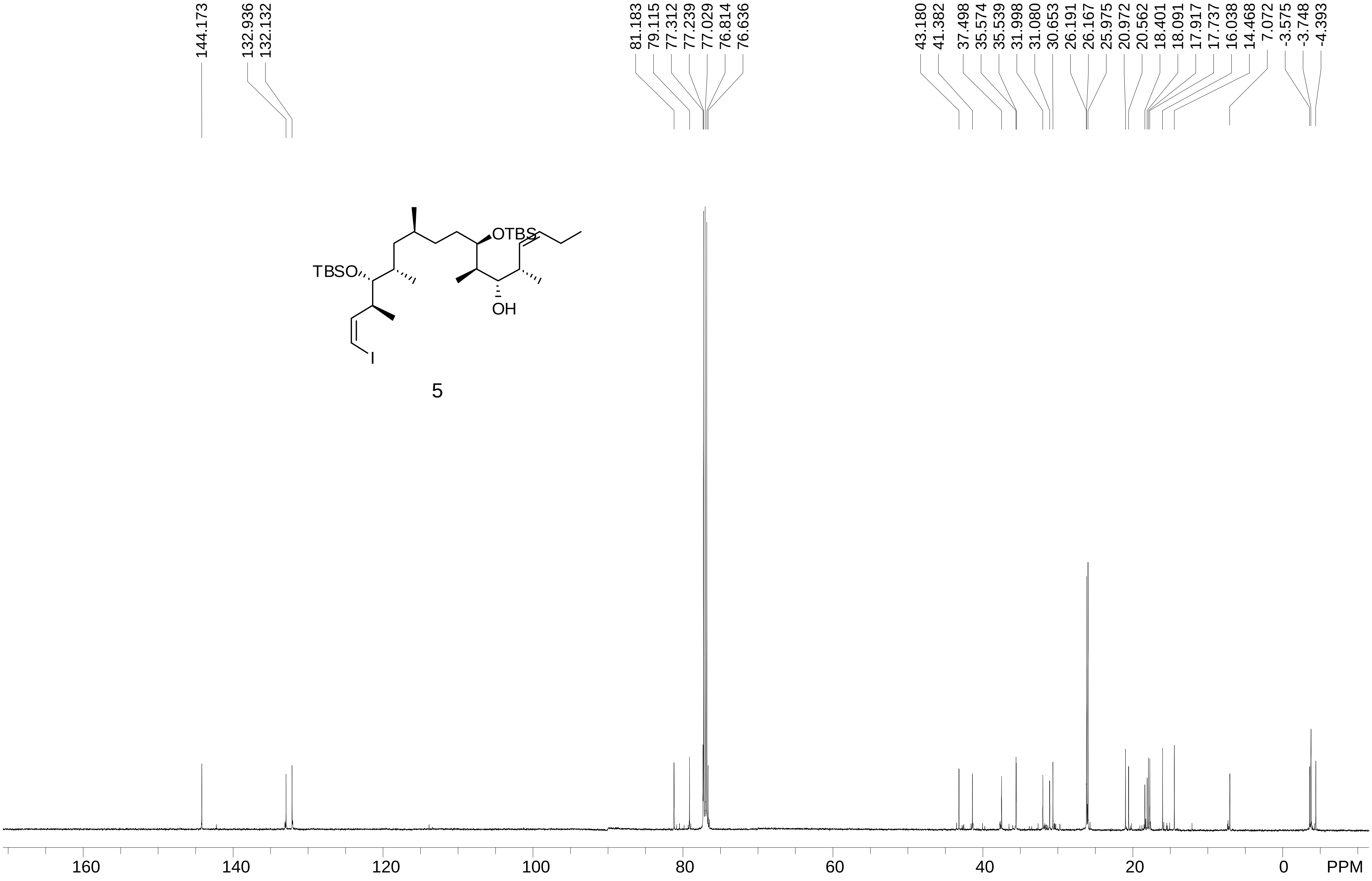
144.173
132.936
132.132
81.183
79.115
77.312
77.239
77.029
76.814
76.636
43.180
41.382
37.498
35.574
35.539
31.998
31.080
30.653
26.191
26.167
25.975
20.972
20.562
18.401
18.091
17.917
17.737
16.038
14.468
7.072
-3.575
-3.748
-4.393
TBSO
OTBS
OH
I
5
160
140
120
100
80
60
40
20
0
PPM

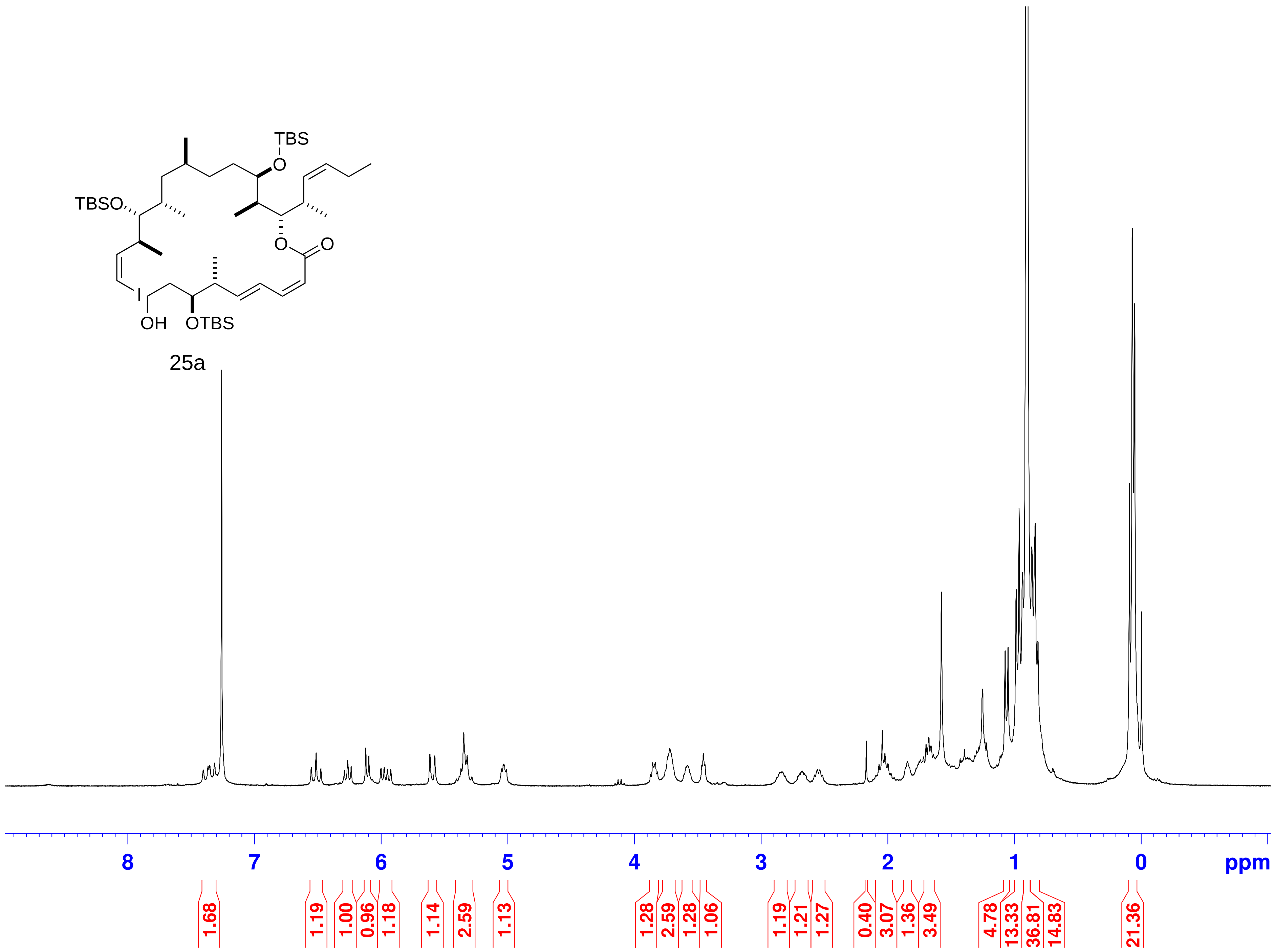
TBS
O
TBSO
OH
OTBS
I
O
O
25a
8
7
6
5
4
3
2
1
0
ppm
1.68
1.19
1.00
0.96
1.18
1.14
2.59
1.13
1.28
2.59
1.28
1.06
1.19
1.21
1.27
0.40
3.07
1.36
3.49
4.78
13.33
36.81
14.83
21.36

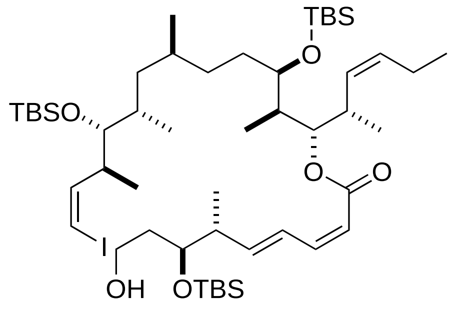
TBS
O
TBSO
O
O
I
OH
OTBS

25a

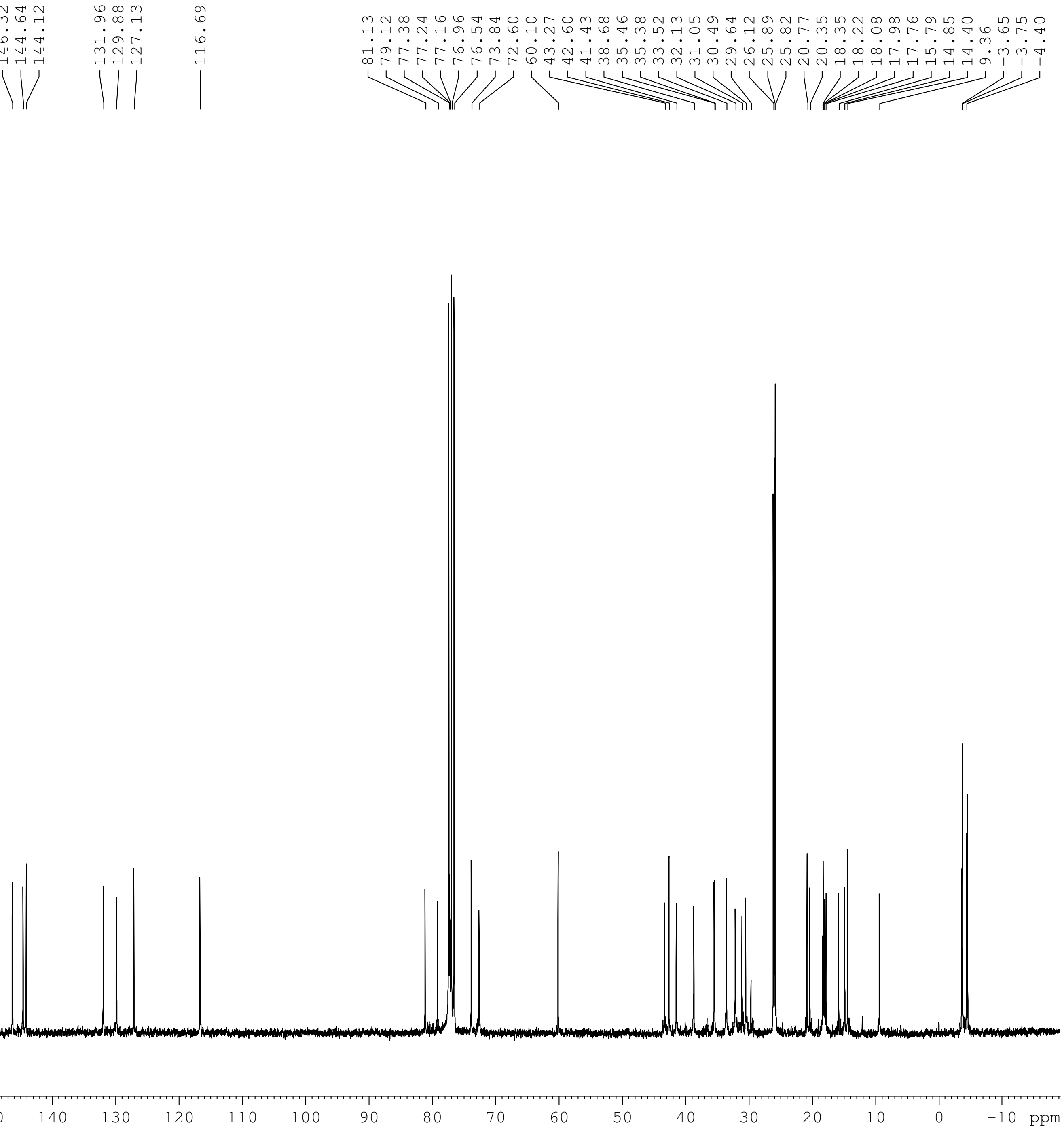
166.08
146.32
144.64
144.12
131.96
129.88
127.13
116.69
81.13
79.12
77.38
77.24
77.16
76.96
76.54
73.84
72.60
60.10
43.27
42.60
41.43
38.68
35.46
35.38
33.52
32.13
31.05
30.49
29.64
26.12
25.89
25.82
20.77
20.35
18.35
18.22
18.08
17.98
17.76
15.79
14.85
14.40
9.36
-3.65
-3.75
-4.40
210 200 190 180 170 160 150 140 130 120 110 100 90 80 70 60 50 40 30 20 10 0 -10 ppm

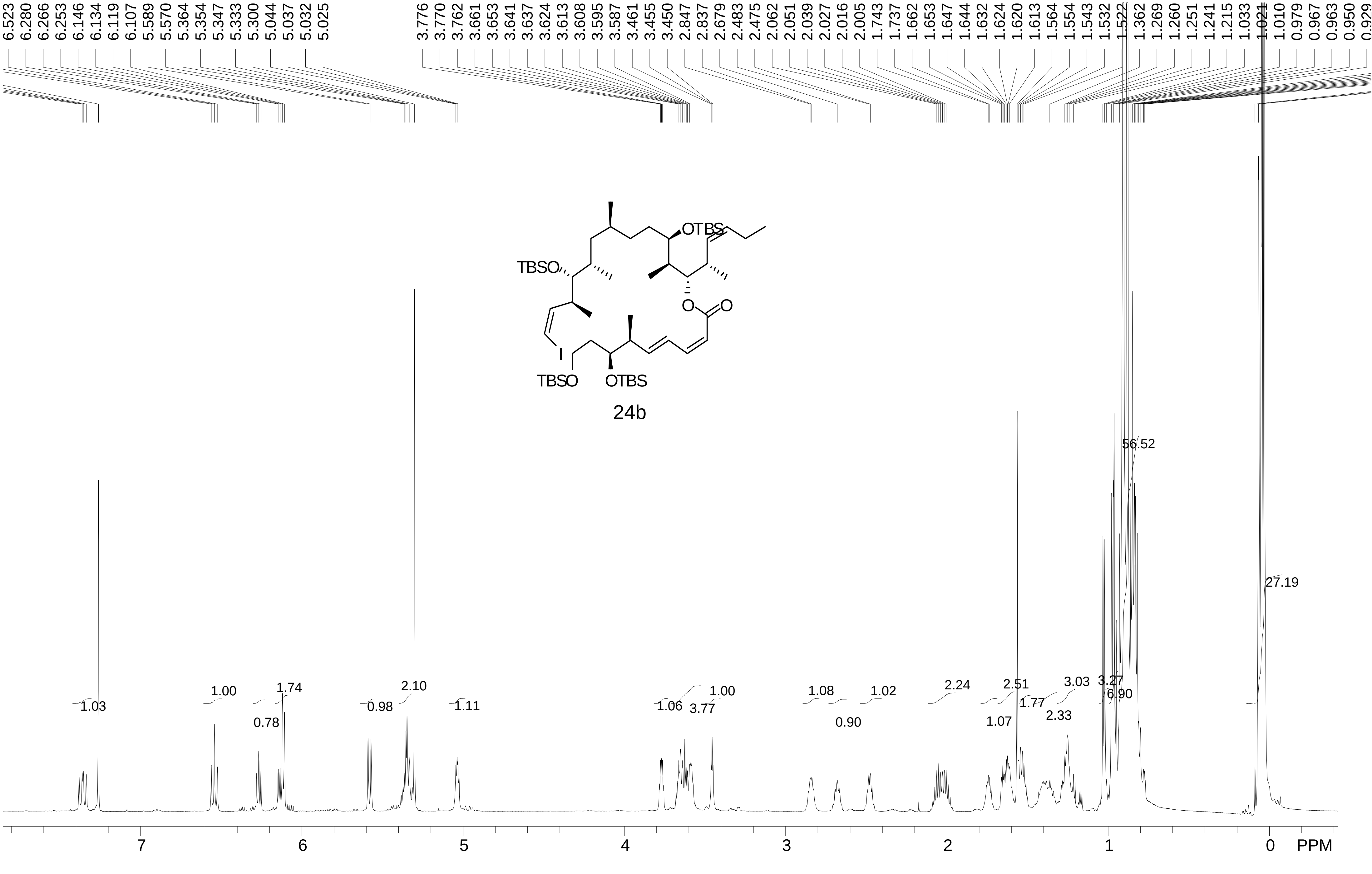

6.523 6.280 6.266 6.253 6.146 6.134 6.119 6.107 5.589 5.570 5.364 5.354 5.347 5.333 5.300 5.044 5.037 5.032 5.025
3.776 3.770 3.762 3.661 3.653 3.641 3.637 3.624 3.613 3.608 3.595 3.587 3.461 3.455 3.450 2.847 2.837 2.679 2.483 2.475 2.062 2.051 2.039 2.027 2.016 2.005 1.743 1.737 1.662 1.653 1.647 1.644 1.632 1.624 1.620 1.613 1.564 1.554 1.543 1.532 1.522 1.362 1.269 1.260 1.251 1.241 1.215 1.033 1.021 1.010 0.979 0.967 0.963 0.950 0.929
OTBS
TBSO
I
O
O
TBSO
OTBS
24b
56.52
27.19
1.03
1.00
0.78
1.74
0.98
2.10
1.11
1.06
3.77
1.00
1.08
0.90
1.02
2.24
1.07
2.51
1.77
3.03
2.33
3.27
6.90
7
6
5
4
3
2
1
0
PPM

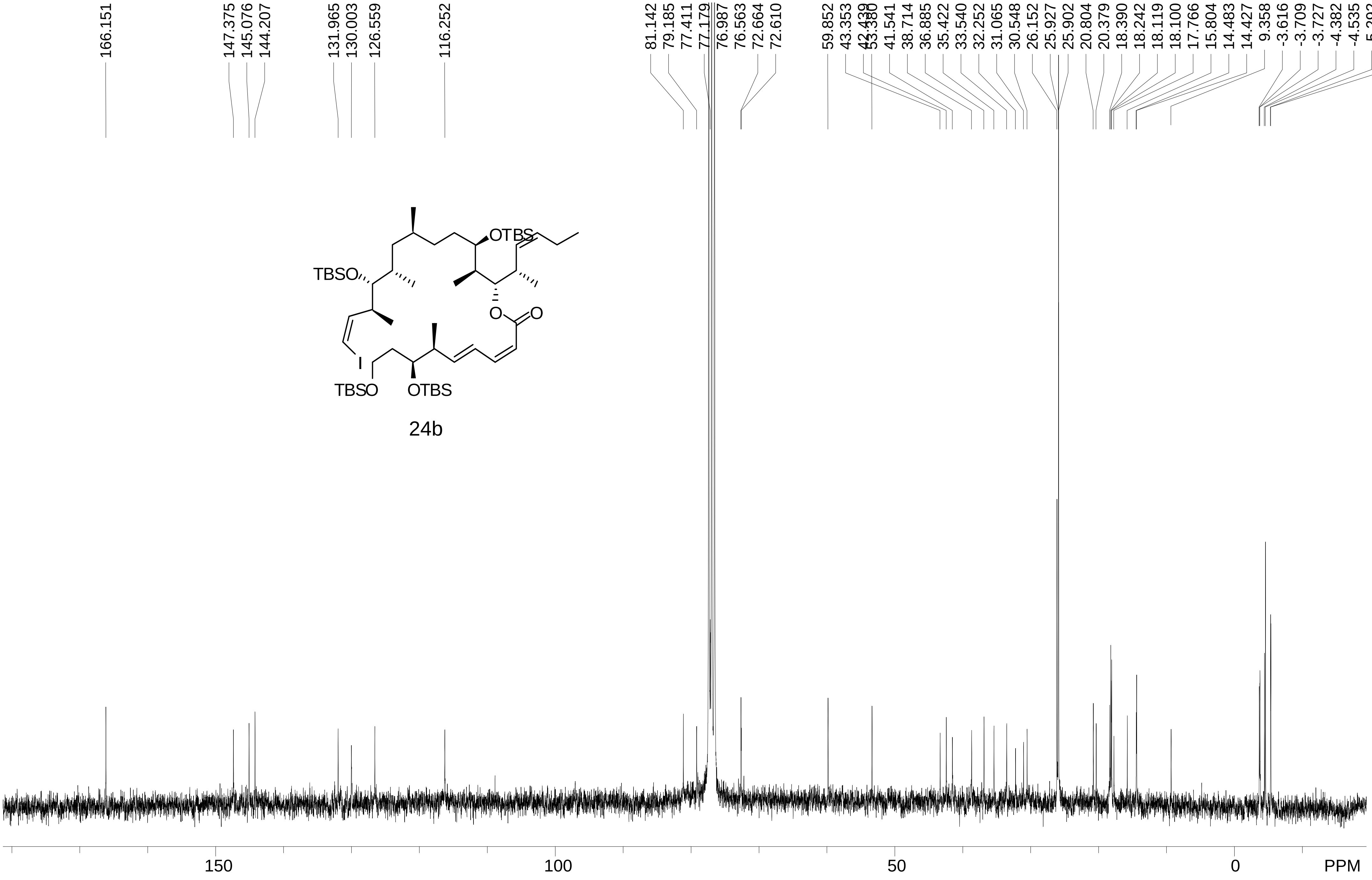
166.151
147.375
145.076
144.207
131.965
130.003
126.559
116.252
81.142
79.185
77.411
77.179
76.987
76.563
72.664
72.610
59.852
43.353
42.439
53.380
41.541
38.714
36.885
35.422
33.540
32.252
31.065
30.548
26.152
25.927
25.902
20.804
20.379
18.390
18.242
18.119
18.100
17.766
15.804
14.483
14.427
9.358
-3.616
-3.709
-3.727
-4.382
-4.535
-5.292
-5.328
OTBS
TBSO
I
TBSO
OTBS
O
O
24b
150
100
50
0
PPM

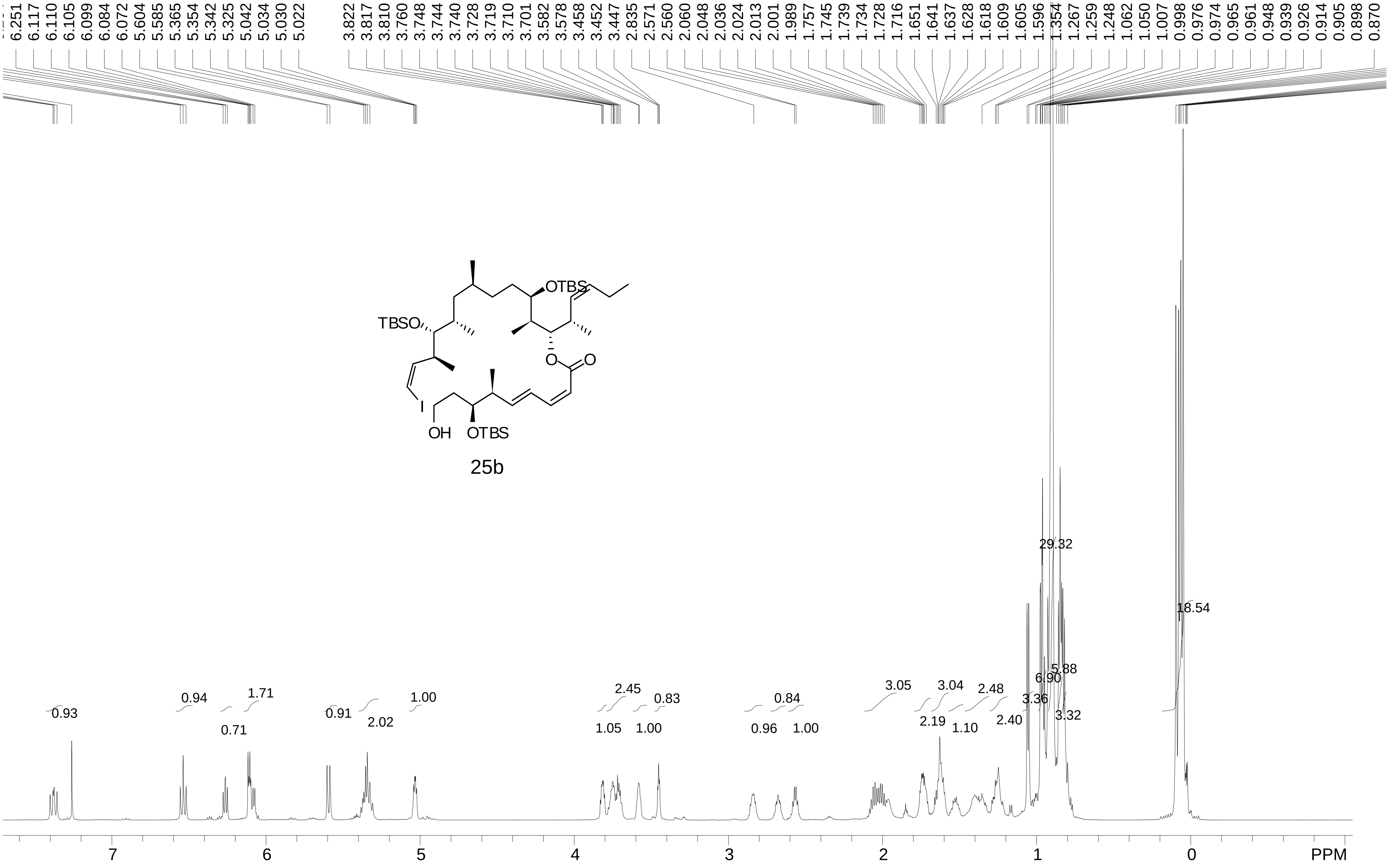
6.251
6.117
6.110
6.105
6.099
6.084
6.072
5.604
5.585
5.365
5.354
5.342
5.325
5.042
5.034
5.030
5.022
3.822
3.817
3.810
3.760
3.748
3.744
3.740
3.728
3.719
3.710
3.701
3.582
3.578
3.458
3.452
3.447
2.835
2.571
2.560
2.060
2.048
2.036
2.024
2.013
2.001
1.989
1.757
1.745
1.739
1.734
1.728
1.716
1.651
1.641
1.637
1.628
1.618
1.609
1.605
1.596
1.354
1.267
1.259
1.248
1.062
1.050
1.007
0.998
0.976
0.974
0.965
0.961
0.948
0.939
0.926
0.914
0.905
0.898
0.870
TBSO
OTBS
O
O
I
OH
OTBS
25b
0.93
0.94
0.71
1.71
0.91
2.02
1.00
2.45
1.05
1.00
0.83
0.96
0.84
1.00
3.05
2.19
3.04
1.10
2.48
2.40
3.36
6.90
29.32
5.88
3.32
18.54
7
6
5
4
3
2
1
0
PPM

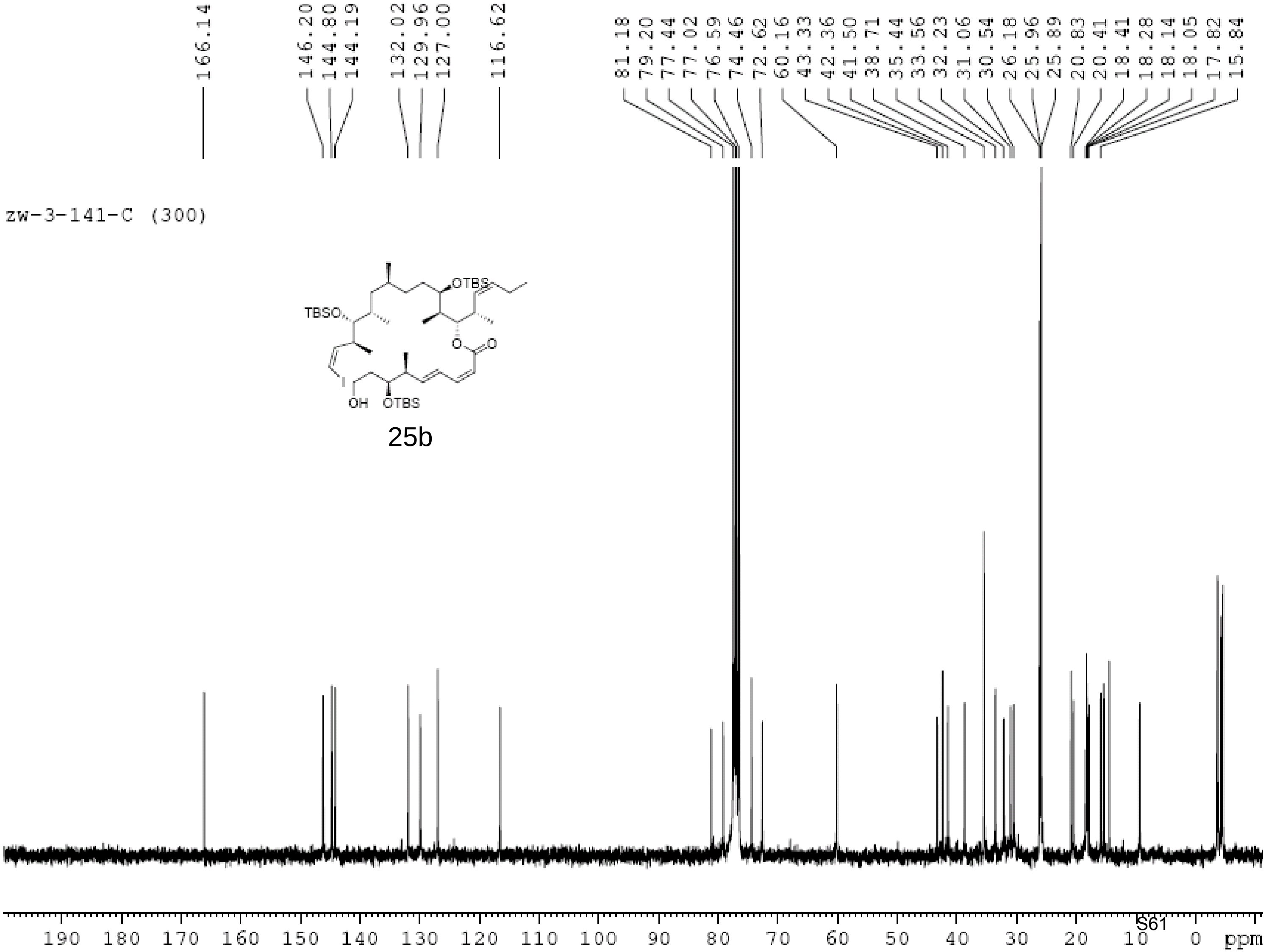
166.14
146.20
144.80
144.19
132.02
129.96
127.00
116.62
81.18
79.20
77.44
77.02
76.59
74.46
72.62
60.16
43.33
42.36
41.50
38.71
35.44
33.56
32.23
31.06
30.54
26.18
25.96
25.89
20.83
20.41
18.41
18.28
18.14
18.05
17.82
15.84
zw-3-141-C (300)
OTBS
TBSO
O
O
I
OH
OTBS
25b
190 180 170 160 150 140 130 120 110 100 90 80 70 60 50 40 30 20 10 0 ppm

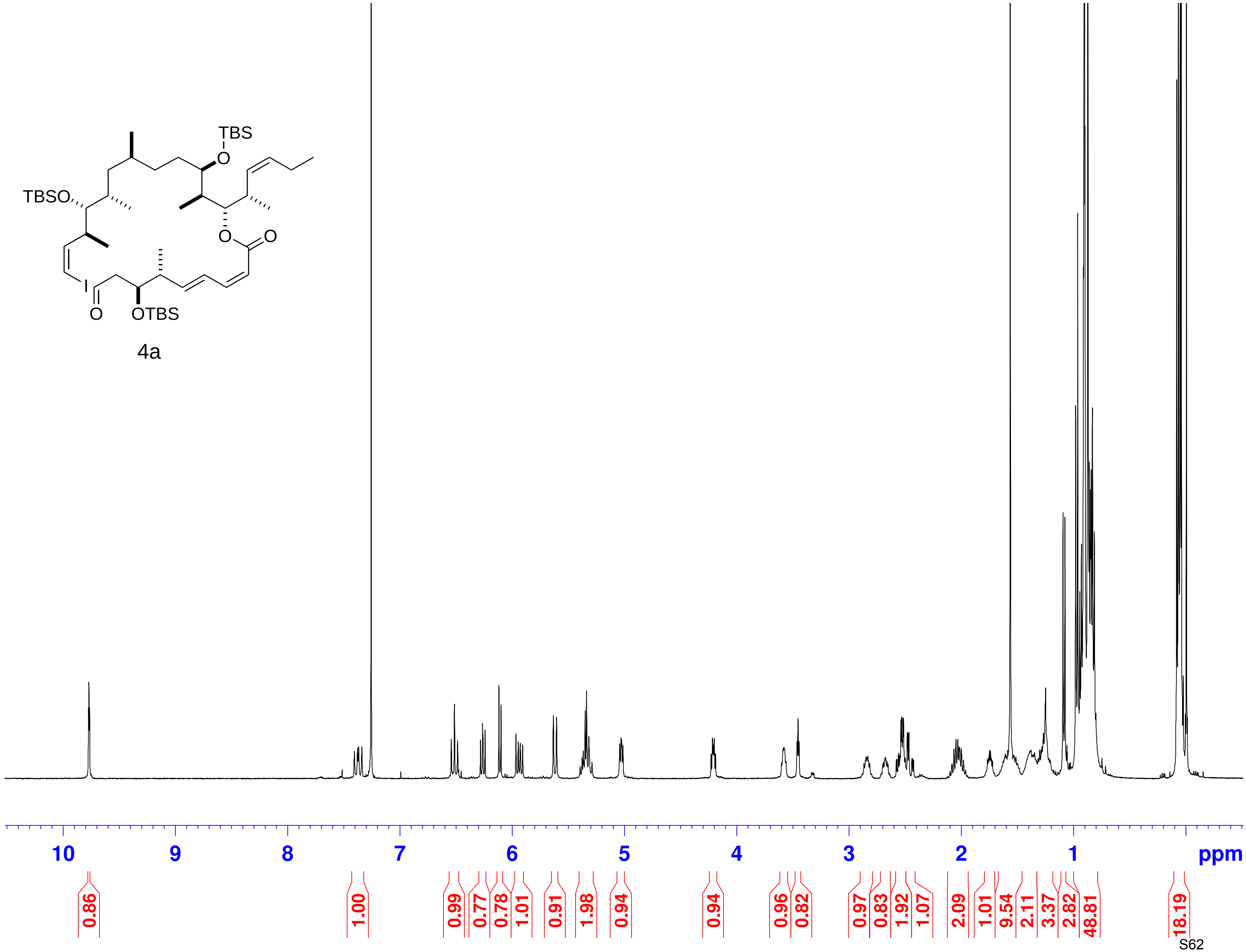
TBS
O
TBSO
O
O
I
O
OTBS
4a
10
9
8
7
6
5
4
3
2
1
ppm
0.86
1.00
0.99
0.77
0.78
1.01
0.91
1.98
0.94
0.94
0.96
0.82
0.97
0.83
1.92
1.07
2.09
1.01
9.54
2.11
3.37
2.82
48.81
18.19

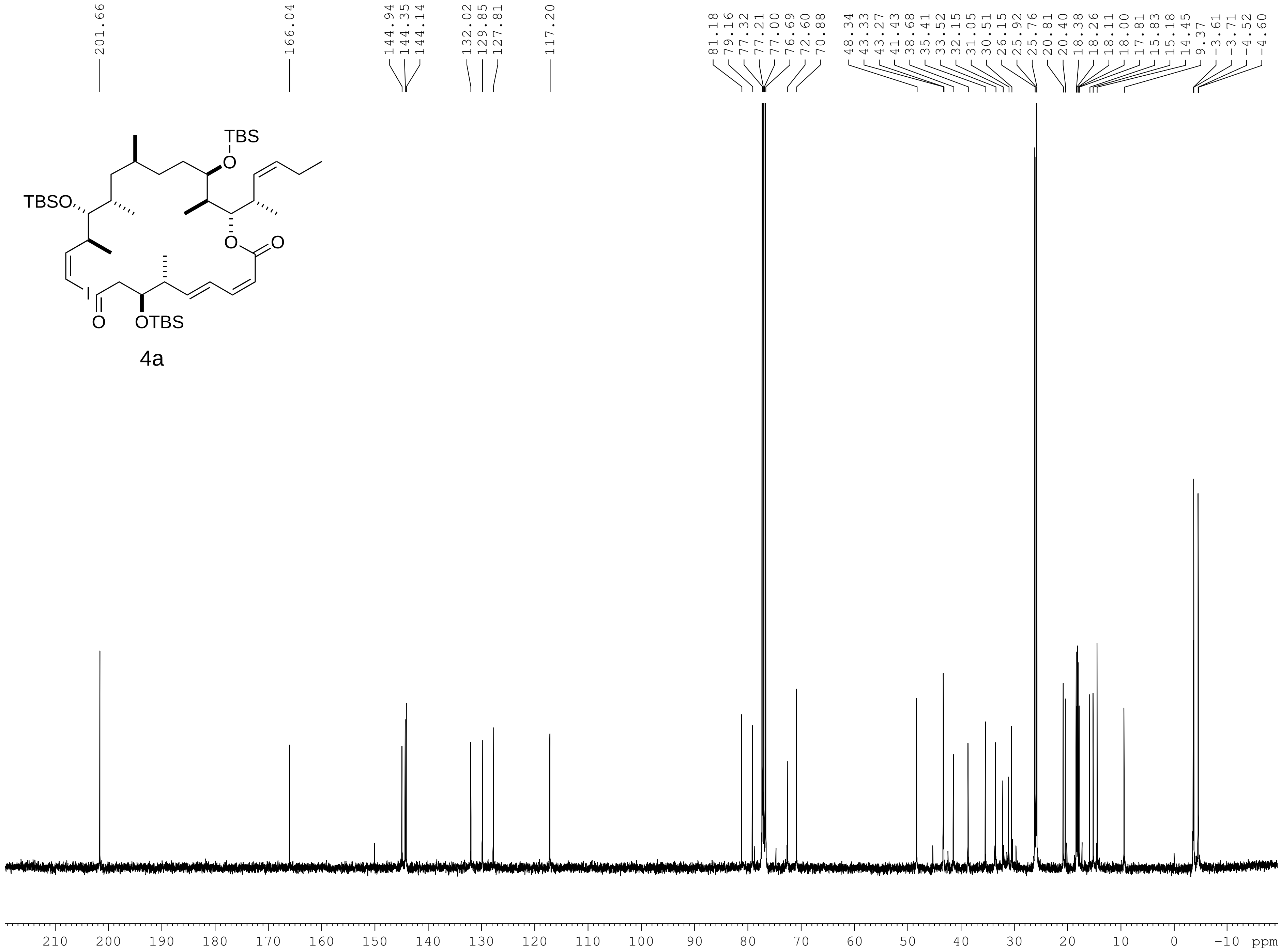
201.66
166.04
144.94
144.35
144.14
132.02
129.85
127.81
117.20
81.18
79.16
77.32
77.21
77.00
76.69
72.60
70.88
48.34
43.33
43.27
41.43
38.68
35.41
33.52
32.15
31.05
30.51
26.15
25.92
25.76
20.81
20.40
18.38
18.26
18.11
18.00
17.81
15.83
15.18
14.45
9.37
-3.61
-3.71
-4.52
-4.60
TBS
O
TBSO
O
O
I
O
OTBS
4a
210 200 190 180 170 160 150 140 130 120 110 100 90 80 70 60 50 40 30 20 10 0 -10 ppm

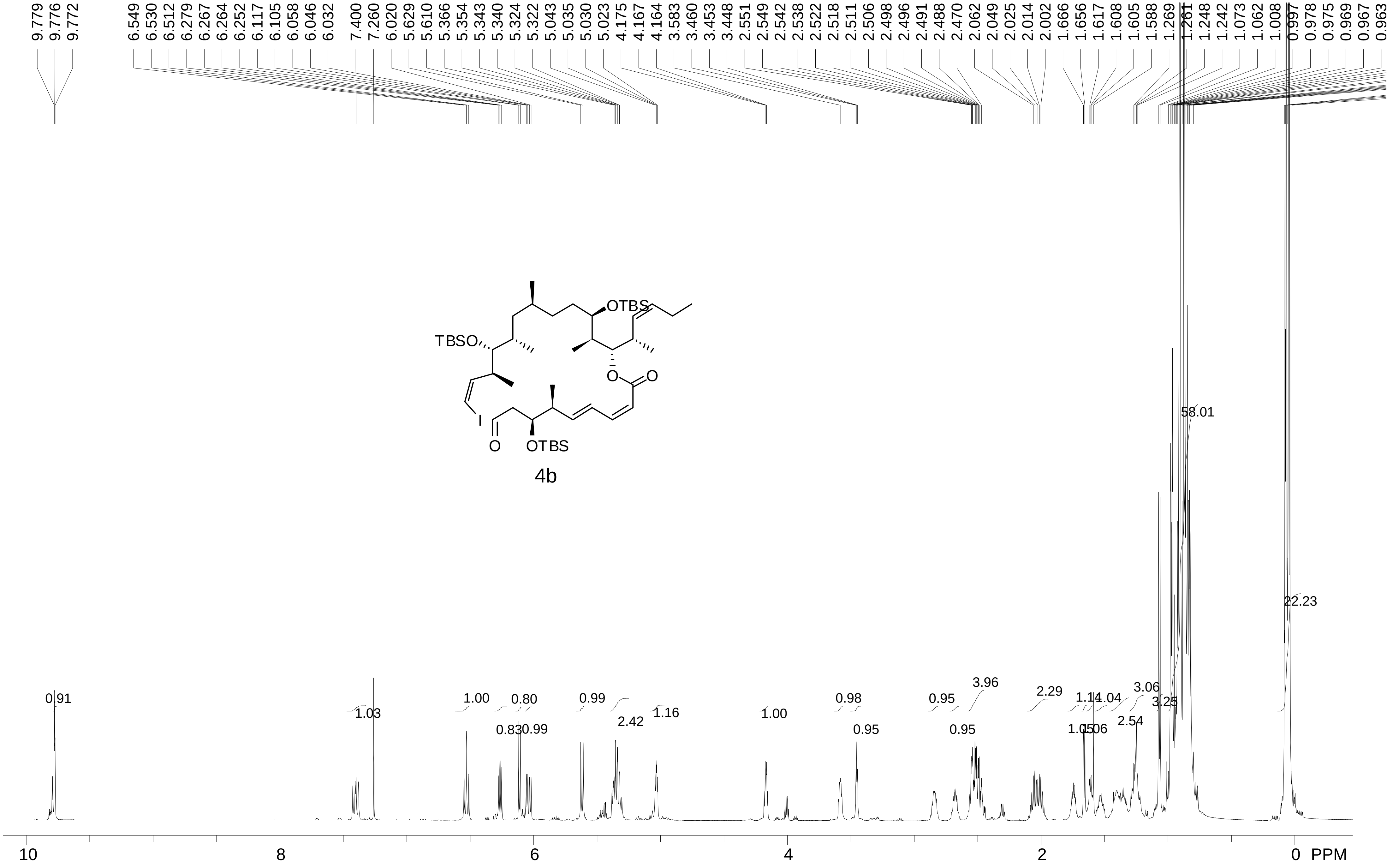
9.779 9.776 9.772 6.549 6.530 6.512 6.279 6.267 6.264 6.252 6.117 6.105 6.058 6.046 6.032 7.400 7.260 6.020 5.629 5.610 5.366 5.354 5.343 5.340 5.324 5.322 5.043 5.035 5.030 5.023 4.175 4.167 4.164 3.583 3.460 3.453 3.448 2.551 2.549 2.542 2.538 2.522 2.518 2.511 2.506 2.498 2.496 2.491 2.488 2.470 2.062 2.049 2.025 2.014 2.002 1.666 1.656 1.617 1.608 1.605 1.588 1.269 1.261 1.248 1.242 1.073 1.062 1.008 0.997 0.978 0.975 0.969 0.967 0.963
TBSO
OTBS
O
O
I
O
OTBS
4b
58.01
22.23
0.91
1.03
1.00
0.80
0.83
0.99
0.99
2.42
1.16
1.00
0.98
0.95
0.95
0.95
3.96
2.29
1.04
3.06
3.25
2.54
10
8
6
4
2
0 PPM

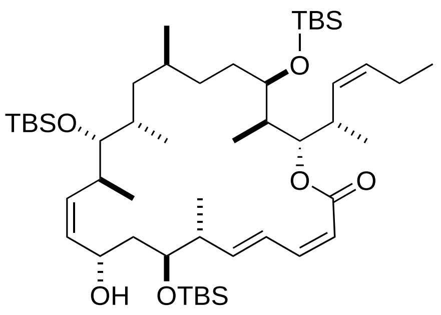
TBS
O
TBSO
O
O
OH
OTBS

26a

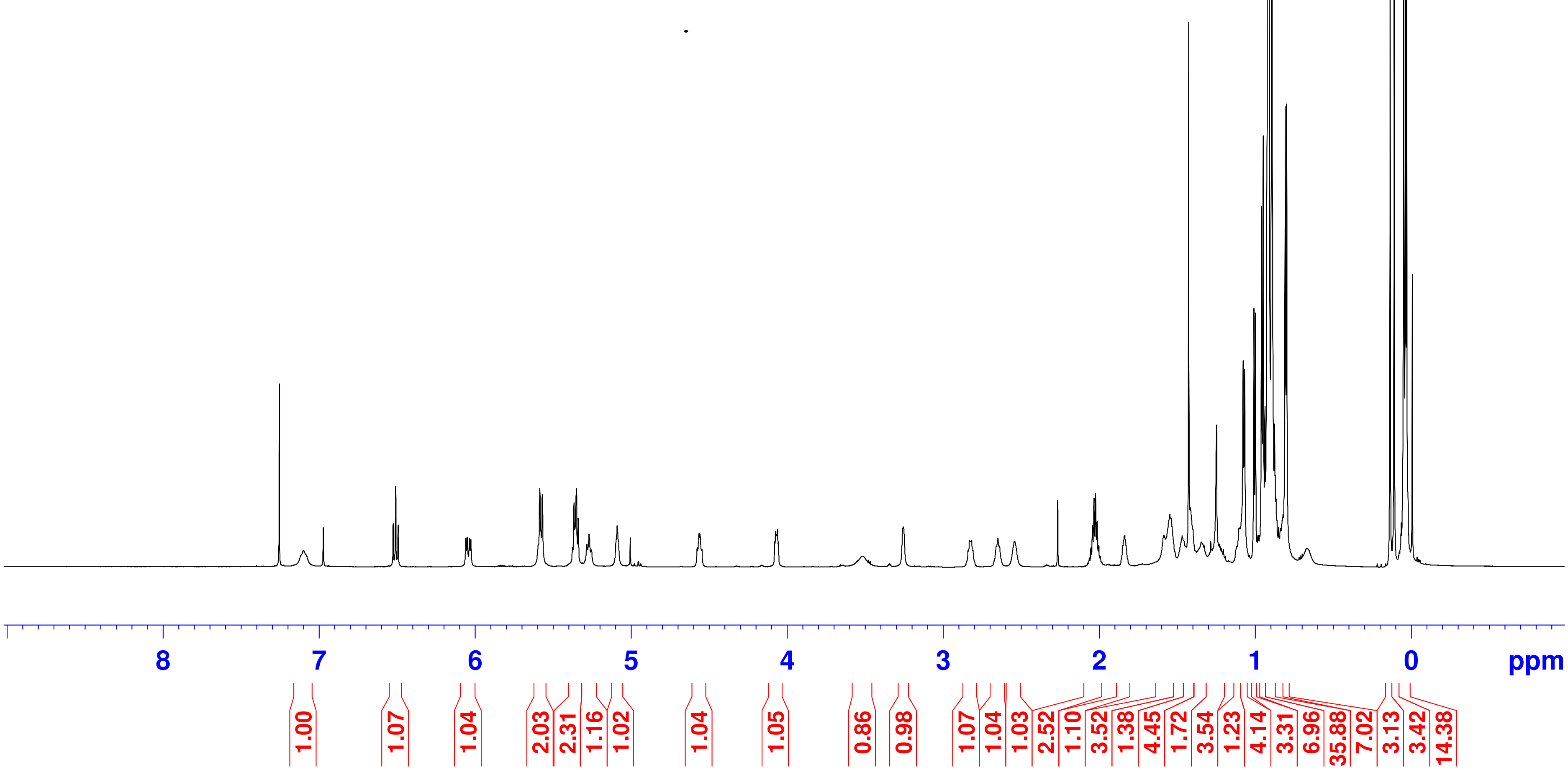
8
7
6
5
4
3
2
1
0
ppm
1.00
1.07
1.04
2.03
2.31
1.16
1.02
1.04
1.05
0.86
0.98
1.07
1.04
1.03
2.52
1.10
3.52
1.38
4.45
1.72
3.54
1.23
4.14
3.31
6.96
35.88
7.02
3.13
3.42
14.38

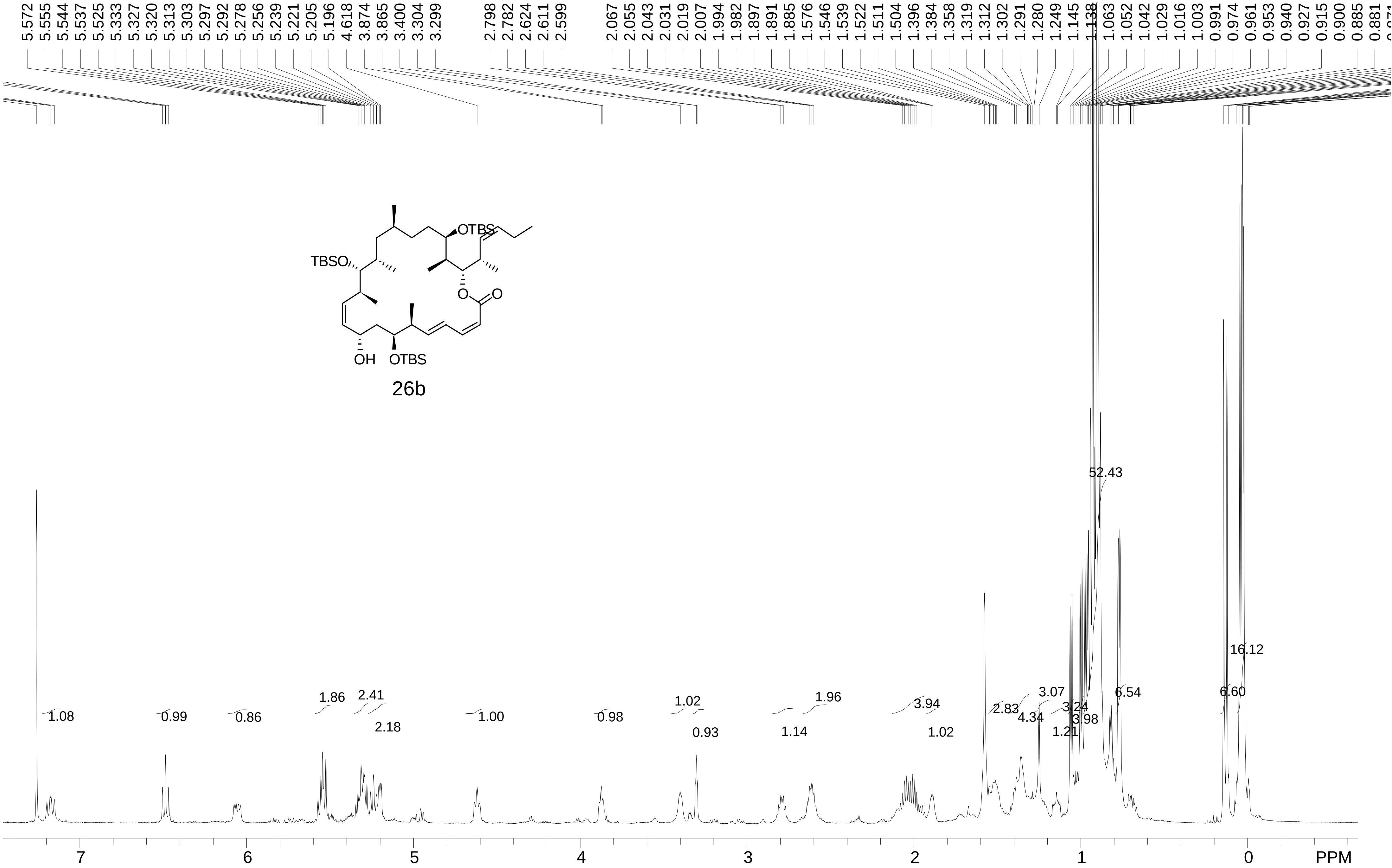
5.572
5.555
5.544
5.537
5.525
5.333
5.327
5.320
5.313
5.303
5.297
5.292
5.278
5.256
5.239
5.221
5.205
5.196
4.618
3.874
3.865
3.400
3.304
3.299
2.798
2.782
2.624
2.611
2.599
2.067
2.055
2.043
2.031
2.019
2.007
1.994
1.982
1.897
1.891
1.885
1.576
1.546
1.539
1.522
1.511
1.504
1.396
1.384
1.358
1.319
1.312
1.302
1.291
1.280
1.249
1.145
1.138
1.063
1.052
1.042
1.029
1.016
1.003
0.991
0.974
0.961
0.953
0.940
0.927
0.915
0.900
0.885
0.881
TBSO
OTBS
OH
OTBS
O
O
26b
52.43
16.12
1.08
0.99
0.86
1.86
2.41
2.18
1.00
0.98
1.02
0.93
1.14
1.96
3.94
1.02
2.83
4.34
3.07
3.24
3.98
1.21
6.54
6.60
7
6
5
4
3
2
1
0
PPM

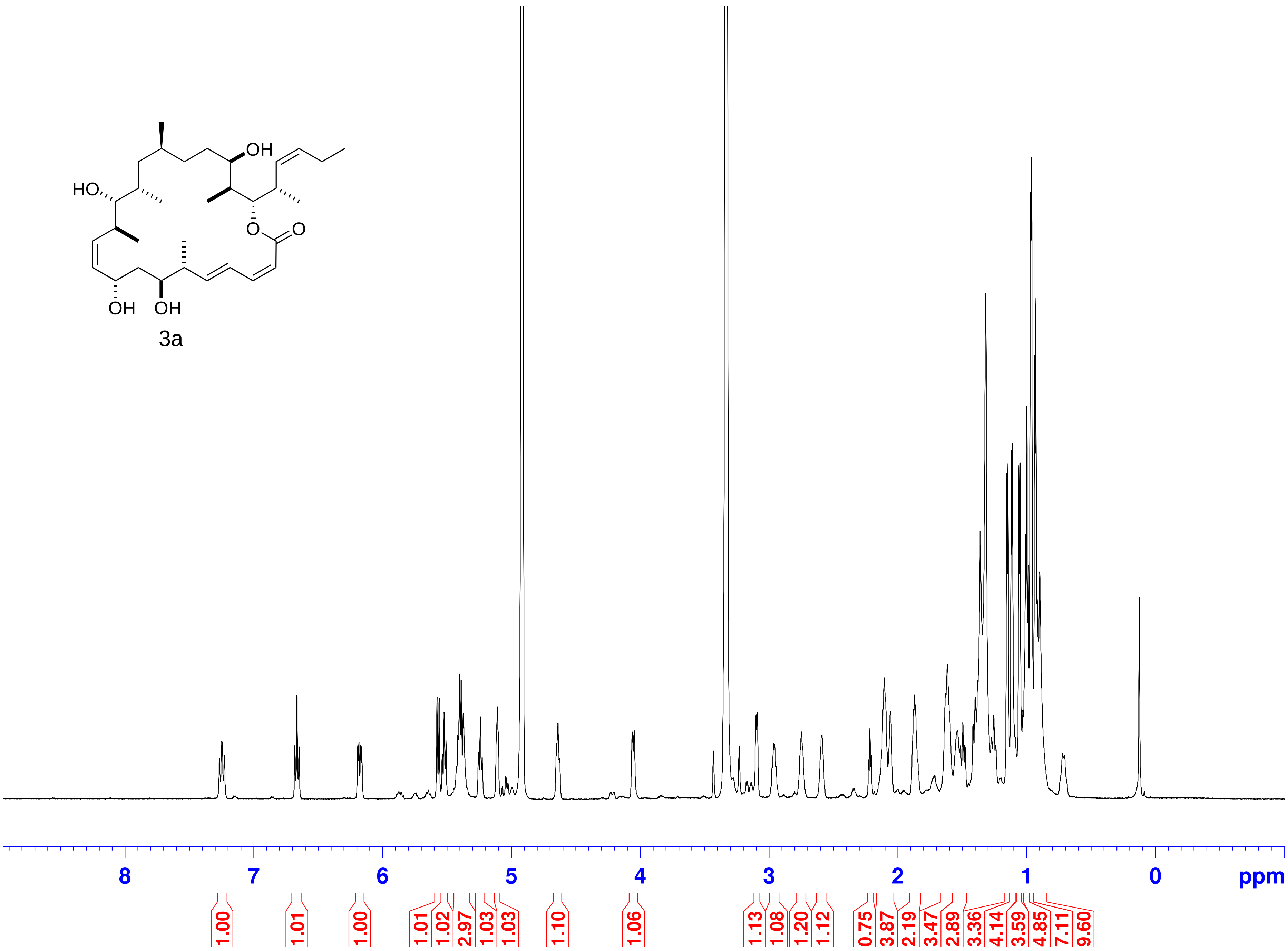
OH
HO
O
O
OH OH
3a
8
7
6
5
4
3
2
1
0
ppm
1.00
1.01
1.00
1.01
1.02
2.97
1.03
1.03
1.10
1.06
1.13
1.08
1.20
1.12
0.75
3.87
2.19
3.47
2.89
3.36
4.14
3.59
4.85
7.11
9.60

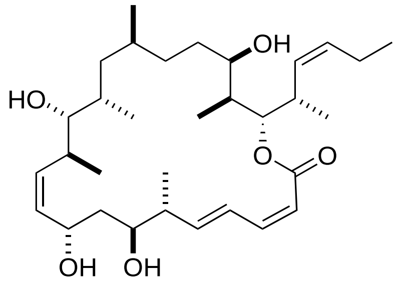
HO
OH
O
O
OH
OH

3a

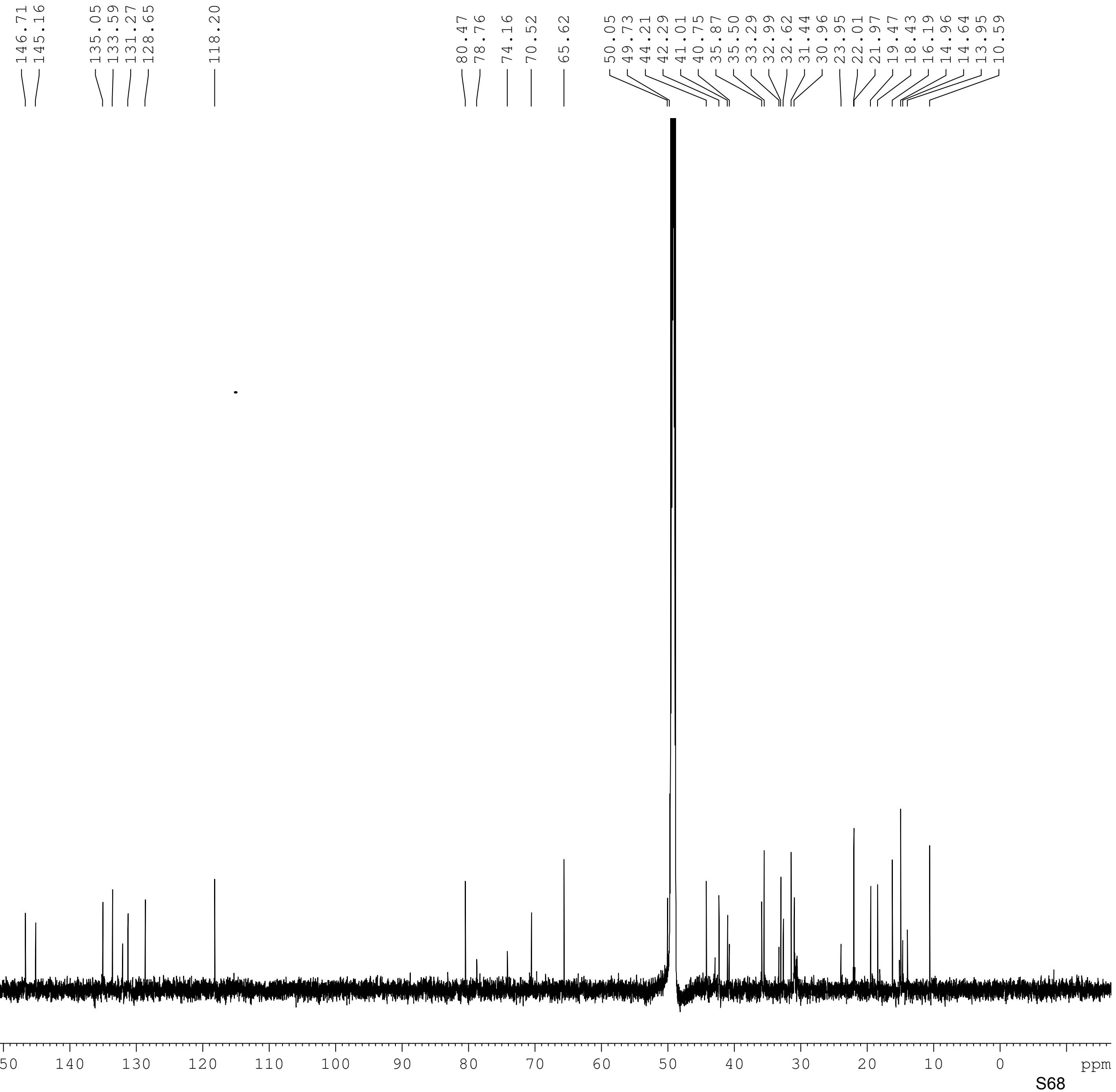
168.24
146.71
145.16
135.05
133.59
131.27
128.65
118.20
80.47
78.76
74.16
70.52
65.62
50.05
49.73
44.21
42.29
41.01
40.75
35.87
35.50
33.29
32.99
32.62
31.44
30.96
23.95
22.01
21.97
19.47
18.43
16.19
14.96
14.64
13.95
10.59
210 200 190 180 170 160 150 140 130 120 110 100 90 80 70 60 50 40 30 20 10 0 ppm

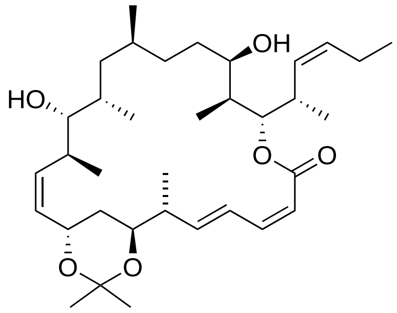
HO
OH
O
O
O
O

Acetonide

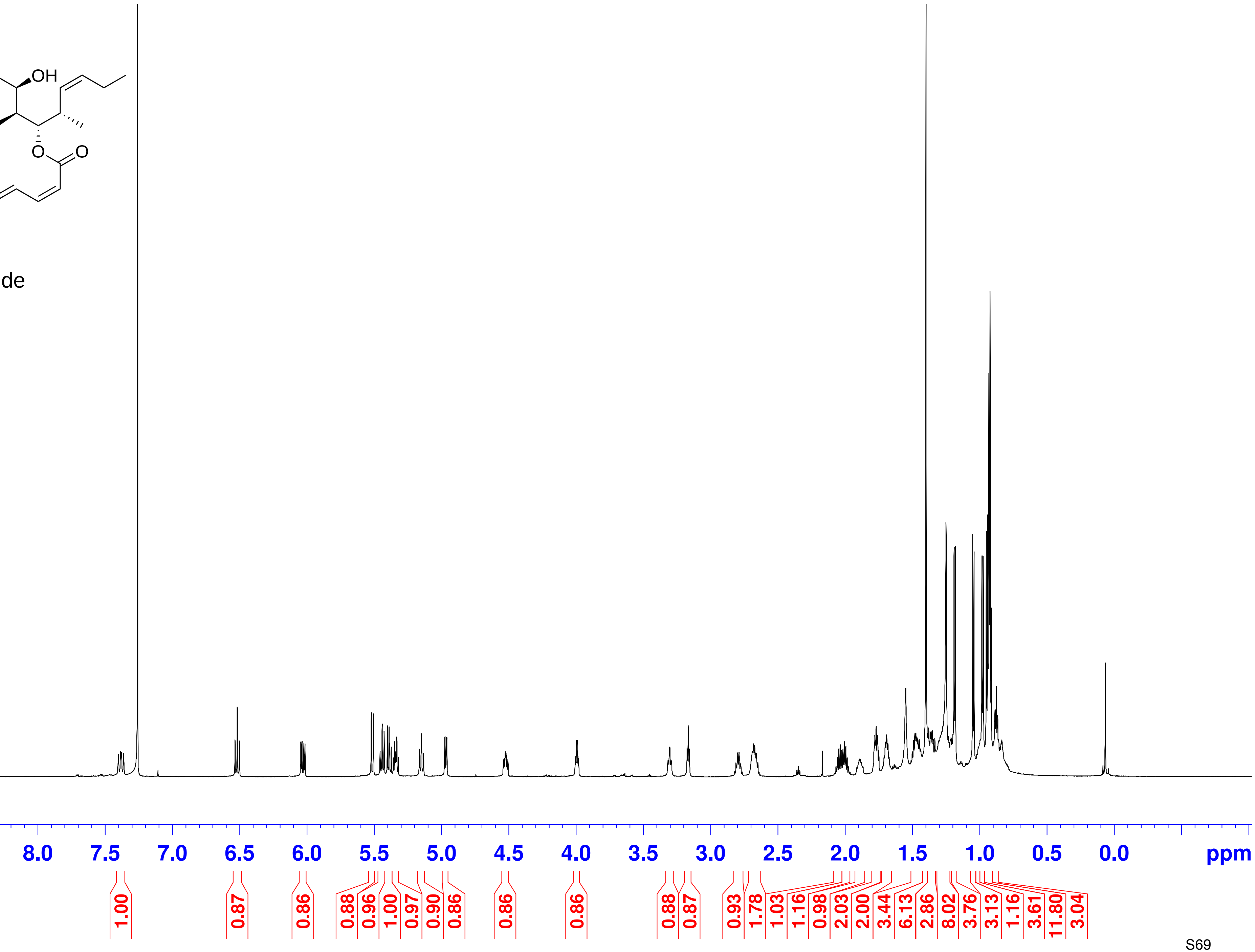
8.5
8.0
7.5
7.0
6.5
6.0
5.5
5.0
4.5
4.0
3.5
3.0
2.5
2.0
1.5
1.0
0.5
0.0
ppm
1.00
0.87
0.86
0.88
0.96
1.00
0.97
0.90
0.86
0.86
0.86
0.88
0.87
0.93
1.78
1.03
1.16
0.98
2.03
2.00
3.44
6.13
2.86
8.02
3.76
3.13
1.16
3.61
11.80
3.04

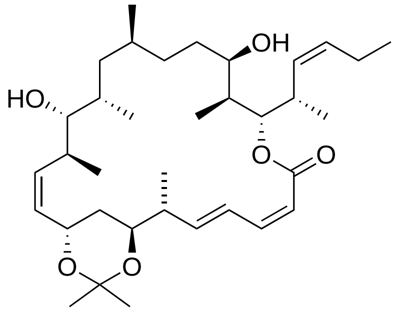
HO
OH
O
O
O
O

Acetonide

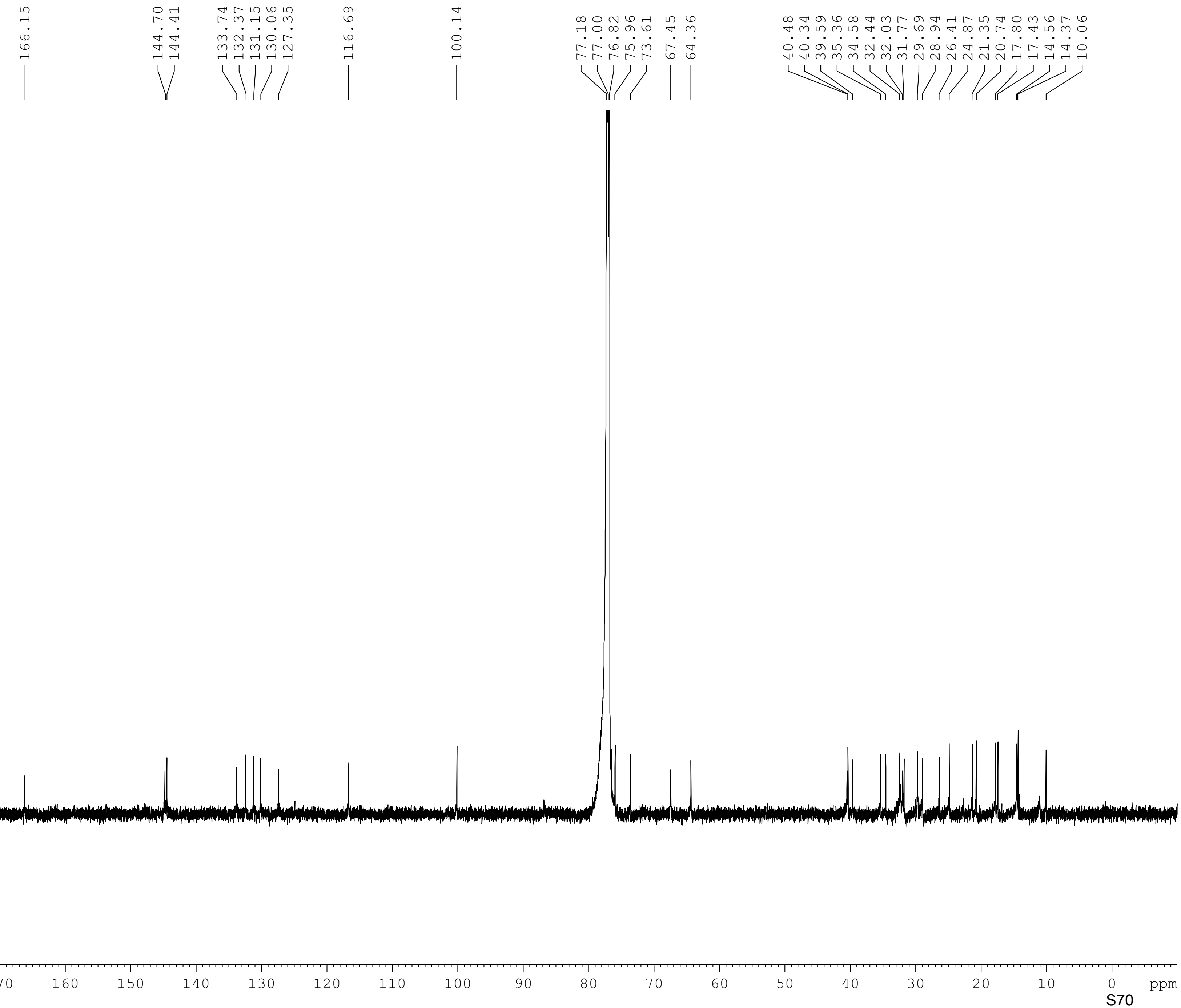
166.15
144.70
144.41
133.74
132.37
131.15
130.06
127.35
116.69
100.14
77.18
77.00
76.82
75.96
73.61
67.45
64.36
40.48
40.34
39.59
35.36
34.58
32.44
32.03
31.77
29.69
28.94
26.41
24.87
21.35
20.74
17.80
17.43
14.56
14.37
10.06
200 190 180 170 160 150 140 130 120 110 100 90 80 70 60 50 40 30 20 10 0 ppm

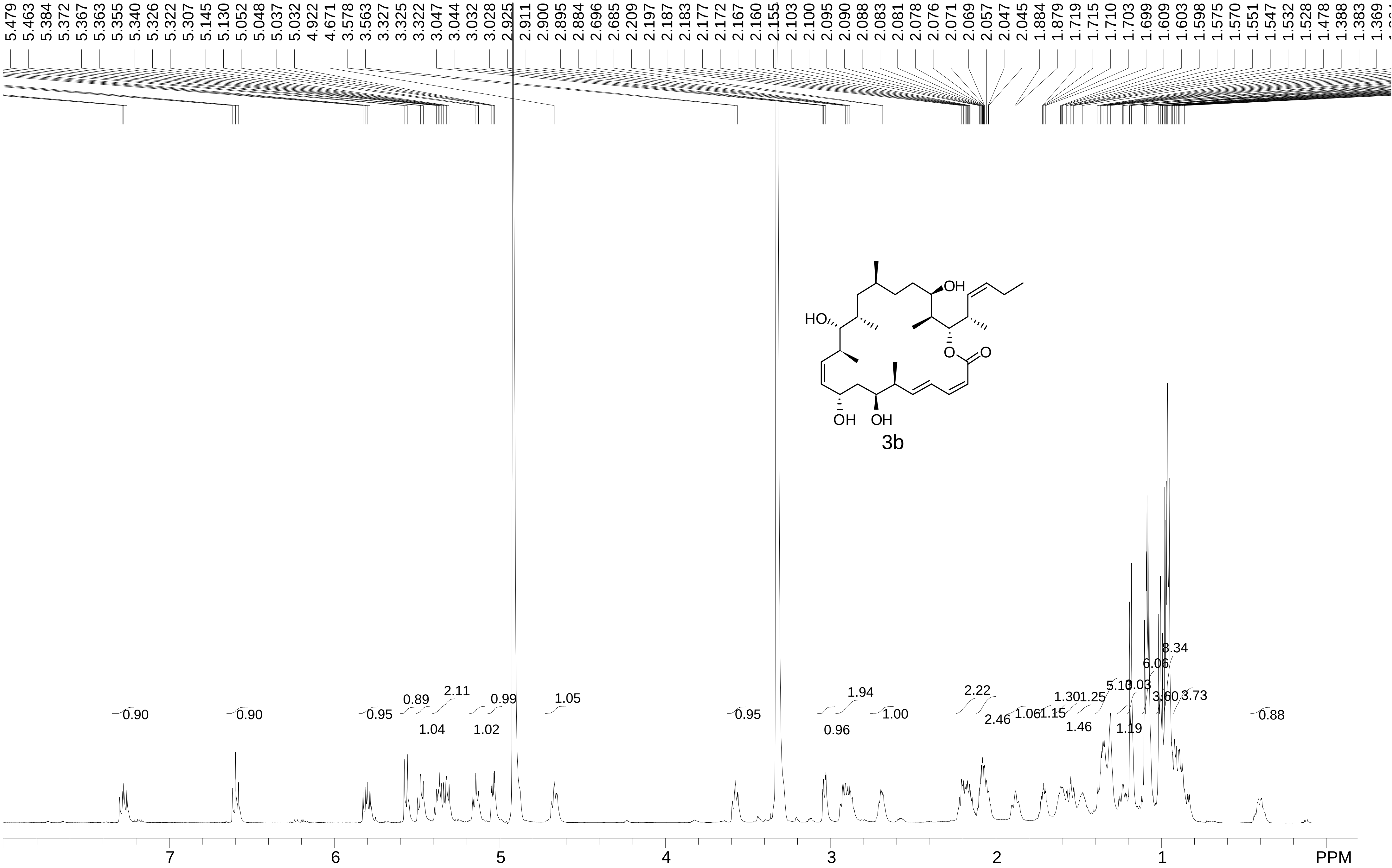
5.479
5.463
5.384
5.372
5.367
5.363
5.355
5.340
5.326
5.322
5.307
5.145
5.130
5.052
5.048
5.037
5.032
4.922
4.671
3.578
3.563
3.327
3.325
3.322
3.047
3.044
3.032
3.028
2.925
2.911
2.900
2.895
2.884
2.696
2.685
2.209
2.197
2.187
2.183
2.177
2.172
2.167
2.160
2.155
2.103
2.100
2.095
2.090
2.088
2.083
2.081
2.078
2.076
2.071
2.069
2.057
2.047
2.045
1.884
1.879
1.719
1.715
1.710
1.703
1.699
1.609
1.603
1.598
1.575
1.570
1.551
1.547
1.532
1.528
1.478
1.388
1.383
1.369
HO
OH
O
O
OH
OH
3b
0.90
0.90
0.95
0.89
1.04
2.11
1.02
0.99
1.05
0.95
0.96
1.94
1.00
2.22
2.46
1.06
1.15
1.30
1.46
1.25
5.10
3.03
1.19
6.06
8.34
3.60
3.73
0.88
7
6
5
4
3
2
1
PPM

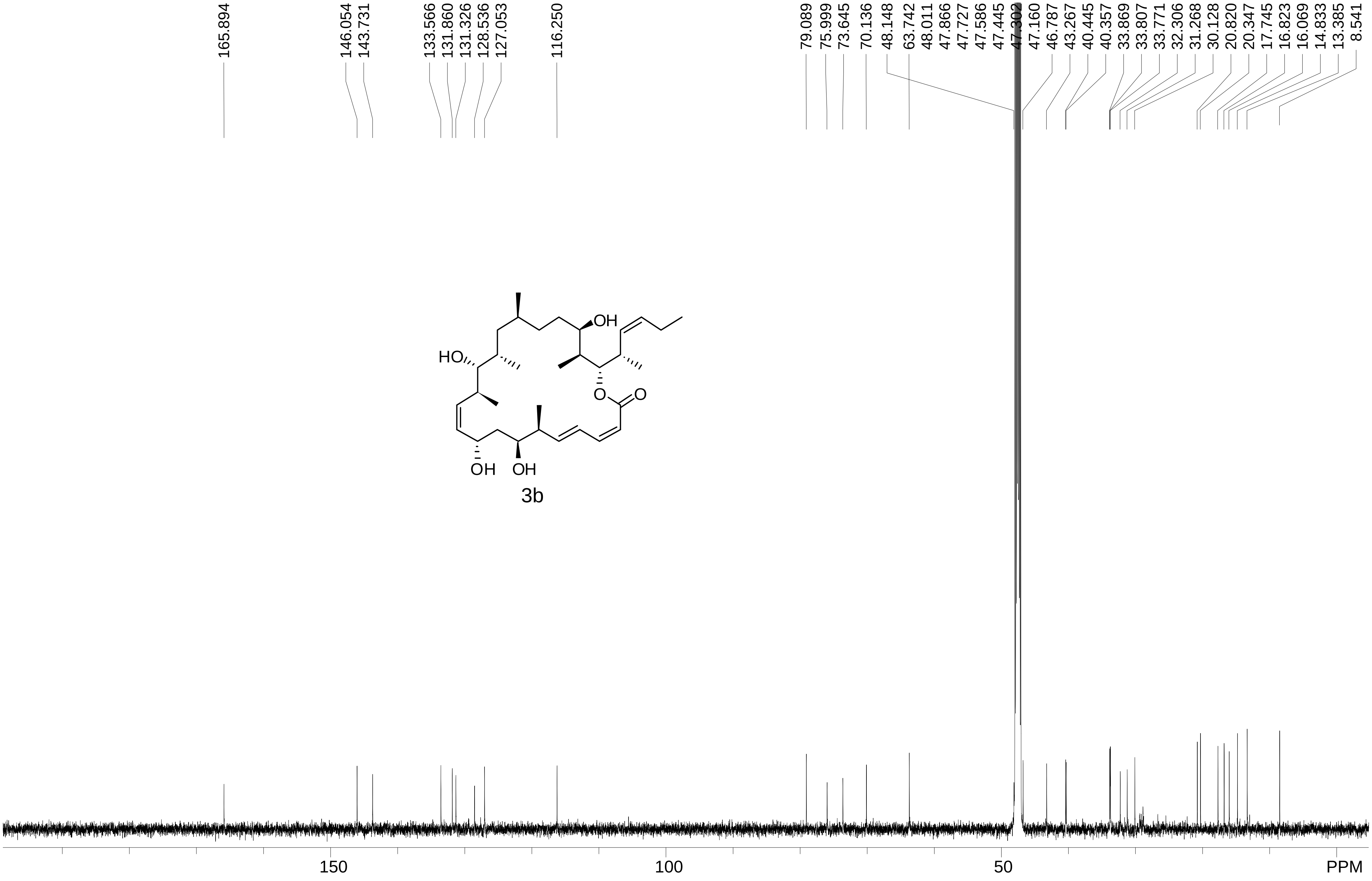
165.894
146.054
143.731
133.566
131.860
131.326
128.536
127.053
116.250
79.089
75.999
73.645
70.136
48.148
63.742
48.011
47.866
47.727
47.586
47.445
47.302
47.160
46.787
43.267
40.445
40.357
33.869
33.807
33.771
32.306
31.268
30.128
20.820
20.347
17.745
16.823
16.069
14.833
13.385
8.541
OH
HO
O
O
OH
OH
3b
150
100
50
PPM
